# BIND&MODIFY: a long-range method for single-molecule mapping of chromatin modifications in eukaryotes

**DOI:** 10.1186/s13059-023-02896-y

**Published:** 2023-03-29

**Authors:** Zhe Weng, Fengying Ruan, Weitian Chen, Zhichao Chen, Yeming Xie, Meng Luo, Zhe Xie, Chen Zhang, Juan Wang, Yuxin Sun, Yitong Fang, Mei Guo, Chen Tan, Wenfang Chen, Yiqin Tong, Yaning Li, Hongqi Wang, Chong Tang

**Affiliations:** 1 BGI Genomics, BGI-Shenzhen, Shenzhen, 518083 China; 2 College of Life Sciences, University of Chinese Academy of Sciences, Beijing, 100049 China; 3 Department of Biology, Cell Biology and Physiology, University of Copenhagen 13, 2100 Copenhagen, Denmark

**Keywords:** Epigenetics, Histone modification, CpG methylation, H3K27me3, CTCF, m^6^A, Methyltransferase

## Abstract

Epigenetic modifications of histones are associated with development and pathogenesis of disease. Existing approaches cannot provide insights into long-range interactions and represent the average chromatin state. Here we describe BIND&MODIFY, a method using long-read sequencing for profiling histone modifications and transcription factors on individual DNA fibers. We use recombinant fused protein A-M.EcoGII to tether methyltransferase M.EcoGII to protein binding sites to label neighboring regions by methylation. Aggregated BIND&MODIFY signal matches bulk ChIP-seq and CUT&TAG. BIND&MODIFY can simultaneously measure histone modification status, transcription factor binding, and CpG 5mC methylation at single-molecule resolution and also quantifies correlation between local and distal elements.

**Supplementary Information:**

The online version contains supplementary material available at 10.1186/s13059-023-02896-y.

## Background

The genome in each cell of a multicellular organism is identical and remains reasonably static, whereas the epigenome is dynamic [1]. The epigenome varies in different cell types and plays significant roles in various biological processes, including those related to stem cell differentiation [2], the nervous system [3], cell aging, and disease development [4]. However, the current methods used to study the epigenome demonstrate unclear results, with epigenome-related information often being shadowed by that of the genome, which can be directly sequenced. The proposed solutions are based on extracting the epigenome signal using enzymatic or chemical approaches and revealing accessible as well as protected locations.

Therefore, current interest is centered on collecting genomes and comparing genome-wide chromatin modification to identify the epigenetic changes that accompany cell differentiation, environmental signaling, and disease development [5]. To determine if a transcription factor, as a “protein of interest,” binds to a DNA sequence, or to locate histone modifications, chromatin immunoprecipitation is performed followed by sequencing (ChIP-seq) [6]. Large-scale ChIP-seq is based on antibody-mediated precipitation of DNA sequences crosslinked with the targeted proteins, followed by direct ultra-high-throughput DNA sequencing [7]. ChIP-seq facilitated the identification of protein-binding motifs and revealed non-canonical protein-binding motifs. Moreover, these motifs have been extensively investigated to elucidate the biological functions of histone acetylation and methylation, transcription factors, and other molecules involved in epigenome modulation [8–14]. Currently, the need for a high amount of DNA input is a major factor restricting the use of ChIP-seq. A variety of analytical methods, such as ChIPmentation [15], CUT&RUN [16], and CUT&TAG [17], requiring low amounts of DNA input have been proposed. In the CUT&RUN protocol, MNase is fused to protein A (pA-MNase) to guide the cleavage of chromatin, which acts as the binding site for proteins targeted by specific antibodies across the genome [17]. A conceptually similar study has also been performed using the CUT&TAG protocol, in which transposons were used instead of MNase. Many of the techniques derived from the aforementioned protocols have been improved to enable analysis using single-cell DNA input [17–20]. However, the complex relationship between DNA methylation, chromatin modification, and genome structure variation is often difficult to determine using a single omics tool. Most solutions to this complication involve bisulfite treatment of the immunoprecipitated DNA fragments, which provides information related to DNA methylation as well as histones [21–23].

Most ChIP-seq technologies use short-read sequences prepared using NGS, and the downstream analysis is based on the peak calling algorithm with statistical analysis of populated fragments [24, 25]. Hence, when researchers remove the linkage between distal segments, they cannot determine whether the individual chromatin fibers would exhibit the same long-range epigenomic organization. Moreover, owing to the use of short-read sequences, the understanding of methylation and structural variation is limited to the immunoprecipitated genomic region. The limitations of methods using these short-read sequences lie in their lack of labeling methods not requiring cleavage and long-read sequences to preserve necessary epigenetic information in individual chromatin. Therefore, we aimed to develop a new approach named BIND&MODIFY to study the histone methylation and DNA methylation in cancer cells as well as specific transcription factor footprinting. We aimed to examine whether this single-molecule method directly assayed the protein-binding regions, DNA methylation sites, and complex genome structure within a single chromatin fiber at a multi-kilobase scale. The results of our study will provide new insights regarding the regulatory status of genomes across various experimental systems and sequencing platforms.

## Results

### Overview of the BIND&MODIFY method and in vitro validation

**Fig. 1:**
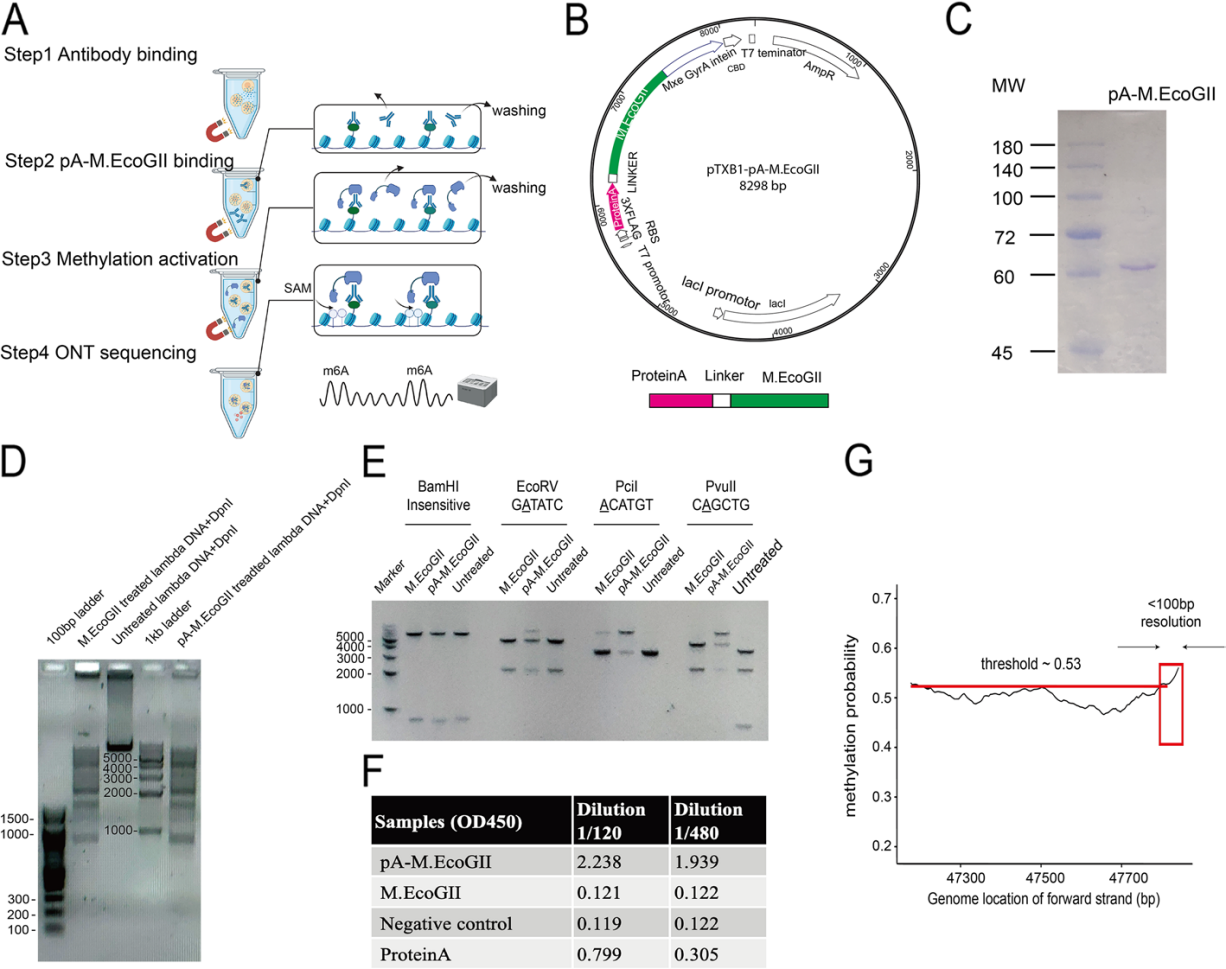
**The experiment concept and in vitro validation of the recombinant protein pA-M.EcoGII in BIND&MODIFY**.** A** The experiment outlines of BIND&MODIFY. After light fixation and permeabilization, the cells were tethered to Concanavalin A magnetic beads (which bind specifically to mannosyl- and glucosyl-containing extracellular glycoproteins on the cell membrane) for purification in the following steps. Step1 Antibody binding; Step 2 pA-M.EcoGII binding; Step 3 Methylation activation; Step 4 ONT sequencing. Firstly, recombinant protein pA-M.EcoGII was tethered to desired sites under antibody guidance. Then the M.EcoGII m6A methylation activity was locally activated with the addition of S-adenosylmethionine to modify the nearby regions to label the genomic DNA with targeted binding proteins. Finally genomic DNA was extracted to prepare the library for ONT nanopore sequencing. After sequencing, the data was processed as genome alignment and m6A base calling (refer to detail protocol). **B** The upper panel showed the plasmid map of the pA-M.EcoGII. The fused pA-M.EcoGII was cloned into pTXB1 plasmid and purified with compatible IMPACT protein purification system. The lower panel showed the expressed fusion protein structure: Protein A-linker-M.EcoGII-intein-CBD. **C** The Coomassie blue gel stain showed the purity of the purified pA-M.EcoGII. **D** Methylation of linear lambda DNA by pA-M.EcoGII activates m6A-site-dependent DpnI restriction endonuclease digestion. The PCR amplified unmethylated lambda DNA was treated with commercial M.EcoGII, no enzyme, and pA-M.EcoGII. The GATC m6A methylation-dependent restriction endonuclease DpnI digestion suggested the comparable methyltransferase activity of the commercial M.EcoGII and our recombinant proteins. DNA marker: 100 bp ladder, 100–1510 bp(left); 1 kb ladder, 250–10,000 bp(right). **E** Methylation of linear dsDNA by pA-M.EcoGII inhibits multiple site-specific methylation-sensitive restriction endonucleases. The unmethylated DNA template was a 7-kb linear dsDNA, which was PCR amplified from pTXB1 plasmid. The DNA template was treated with commercial M.EcoGII, pA-M.EcoGII, and no enzyme. These treated DNA templates were each incubated with four restriction endonucleases (BamHI, EcoRV, PciI, PvuII). The BamHI is the m6A methylation insensitive enzyme, and the EcoRV, PciI, and PvuII are the m6A methylation-sensitive enzyme, with which the digestion could be blocked by corresponding m6A site. Our pA-M.EcoGII recombinant protein showed digestion inhibition on EcoRV, PciI, and PvuII digested samples, better than commercial M.EcoGII, as compared to untreated DNA template. DNA marker, 1 kb ladder, 250–10,000 bp. **F** The antibody affinity assay showed the recombinant pA-M.EcoGII had the affinity to the secondary antibody in two different dilutions (1/120, 1/480, 10 mg/ml). **G** In vitro validation of BIND&MODIFY resolution with single base 5mC containing lambda DNA. A fragment of 700 bp lambda DNA was amplified by PCR, and 5mC was introduced near the end of forward strand only by the modified primers with the precise 5mC site. The 5mC labeled DNA was bound by 5mC antibody and was subsequently treated by BIND&MODIFY method. The m^6^A probability (Megalodon calling probability) of forward strand was calculated and plotted with loci. When the methylation probability cut-off was set at 0.53, the high methylation probability region was observed in 3′ end, overlapping with the expected the 5mC site

The BIND&MODIFY method is based on the indirect labeling of DNA regions bound to the protein of interest using an engineered recombinant fusion protein, protein A-M.EcoGII (pA-M.EcoGII), whose methyltransferase activity can be locally controlled (Fig. 1a). The recently characterized adenosine methyltransferase M.EcoGII can methylate a broader range of genomic DNA in a non-specific sequence manner [26] unlike DamID [27], which methylates only rare GATC motifs in the genome (Additional file 1: Fig. S2a). With the BIND&MODIFY approach, we aimed to leverage the power of an engineered recombinant protein, pA-M.EcoGII, which tethered the methyltransferase close to the specific antibody-bound protein of interest. As a result, the adenines in the DNA sequences adjacent to the protein of interest were methylated in an m^6^A non-specific manner upon activation (Fig. 1a). As there are very few m^6^A modifications in the background of the native mammalian chromosome [28, 29], the artificial m^6^A modification indicating the protein-binding motif could be directly detected by the nanopore [30, 31].

Briefly, the recombinant pA-M.EcoGII was designed by cloning two immunoglobin-binding domains of staphylococcal protein A fused N-terminally with M.EcoGII (Fig. 1b). The amino acid sequence of the linker region between proteins A and M. EcoGII was DDDKEF. The recombinant pA-M.EcoGII was expressed in the *E. coli* system and had a molecular weight of 61 kDa (Fig. 1c). To assess the function of purified recombinant pA-M.EcoGII, the m^6^A-dependent restriction enzyme, DpnI, was used to test the methylation activity of pA-M.EcoGII on lambda DNAs. We found that pA-M.EcoGII demonstrated m^6^A methylation specificity similar to that of commercially available M.EcoGII (NEB) (Fig. 1d). To further evaluate the effectiveness of purified recombinant pA-M.EcoGII, m^6^A-sensitive restriction enzymes (EcoRV, PciI, and PvuII) were used on pTXB1 plasmid DNA treated with pA-M.EcoGII and M.EcoGII. Both pA-M.EcoGII and the commercially available M.EcoGII successfully introduced m^6^A methylation into the plasmid, which inhibited the digestion activity of the m^6^A-sensitive restriction enzymes (Fig. 1e). We also performed ELISA to test the IgG domain binding assessment of pA-M.EcoGII (Fig. 1f). Subsequently, we assessed the m^6^A base calling via nanopore sequencing.

To assess the base calling accuracy in our bioinformatics pipeline, we first tested our m^6^A methylation base calling algorithm on negative control (unmodified DNA). We also used the positive control of pA-M.EcoGII-treated sample that was mostly methylated DNA. We plotted the density map of the m^6^A methylation base calling probability of the negative control and pA-M.EcoGII-treated mostly methylated DNA samples (Additional file 1: Fig. S1a-b). In the pA-M.EcoGII mostly methylated sample, the sharp peak represented the non-modified sites, and the wide peak represented the truly modified sites (Additional file 1: Fig. S 1b). Based on our mathematical model, the m^6^A methylation base calling probability of 0.53 was selected as the cut-off value (Additional file 1: Fig. S1b-g).

To further evaluate the precision and specificity of m^6^A methylation base calling probability cut-off value of 0.53, different treatments of lambda DNA were evaluated: (1) M.EcoGII treatment, (2) pA-M.EcoGII treatment, (3) untreated negative control, (4) Dam treatment. The commercial M. EcoGII and pA-M.EcoGII showed identical m^6^A methylation signals (Additional file 1: Fig. S1c-d); approximately 90% adenines were identified as m^6^A based on the m^6^A methylation probability cut-off of 0.53, which was identical to the labeling efficiency of methyltransferase M.EcoGII [32]. Non-treated lambda DNA showed very little m^6^A methylation signal, and the true negative rate of untreated lambda DNA was 0.78 (Additional file 1: Fig. S1e). As the methyltransferase Dam only methylates adenine in the GATC motif, we identified a specific m^6^A methylation GATC pattern in Dam-treated lambda DNA, which corresponded to approximately 85% of all GATC motifs (Additional file 1: Fig. S1f). Based on Dam-treated lambda DNA data, assuming 100% of Dam methyltransferase labeling efficiency, the precision and specificity of our m^6^A methylation base calling algorithm probability with a cut-off of 0.53 were 0.77 and 0.75. For precise quantification of high confident m^6^A base calling in single-molecular application, we also set m^6^A probability cut-off value to 0.80, and the precision and specificity of our m^6^A methylation base calling algorithm were 0.92 and 0.96, respectively. After assessing the accuracy, precision, and specificity of m^6^A methylation base calling discerned by BIND&MODIFY, we further validated the labeling of the BIND&MODIFY method in vitro.

The frequency of the presence of adenosine is one in every 3 bp in various genomes [32], which is higher than that of the GC/GATC motifs (Additional file 1: Fig. S2). To examine the labeling efficiency of BIND&MODIFY in vitro, we used lambda DNA as a template and a specially designed PCR primer containing a single base 5mC nucleotide to introduce the 5mC modification at a specific location of the PCR product. The 5mC antibody was first applied, followed by pA-M.EcoGII modification of the BIND&MODIFY method. As shown in Fig. 1g, the m^6^A probability (Megalodon calling probability) was plotted, with genomic locations as indicators of the bound pA-M.EcoGII. Regions with a high methylation probability (> 0.53) corresponded to the presence of bound pA-M.EcoGII in the neighboring areas, where the tethered M. EcoGII methylated the DNA sites in a close range (Fig. 1g).

Taken together, the results demonstrate that based on our base calling algorithm, the m^6^A cut-off was accurate, background noise was minimal in vitro, and pA-M.EcoGII and M. EcoGII methyltransferases showed similar methylation efficiency. Overall, we established a bioinformatics pipeline with a highly active pA-M.EcoGII enzyme, which enabled the use of BIND&MODIFY for further in situ applications.

### Validation of BIND&MODIFYin situand a comparison of conventional methods and BIND&MODIFY

We further developed the BIND&MODIFY protocol in situ (Fig. 1a). To demonstrate the in situ efficiency of BIND&MODIFY, we explored the H3K27me3/CTCF status in the breast cancer cell lines MCF-7 and 4T1 using BIND&MODIFY and cross-validated it with ChIP-seq.

**Fig. 2:**
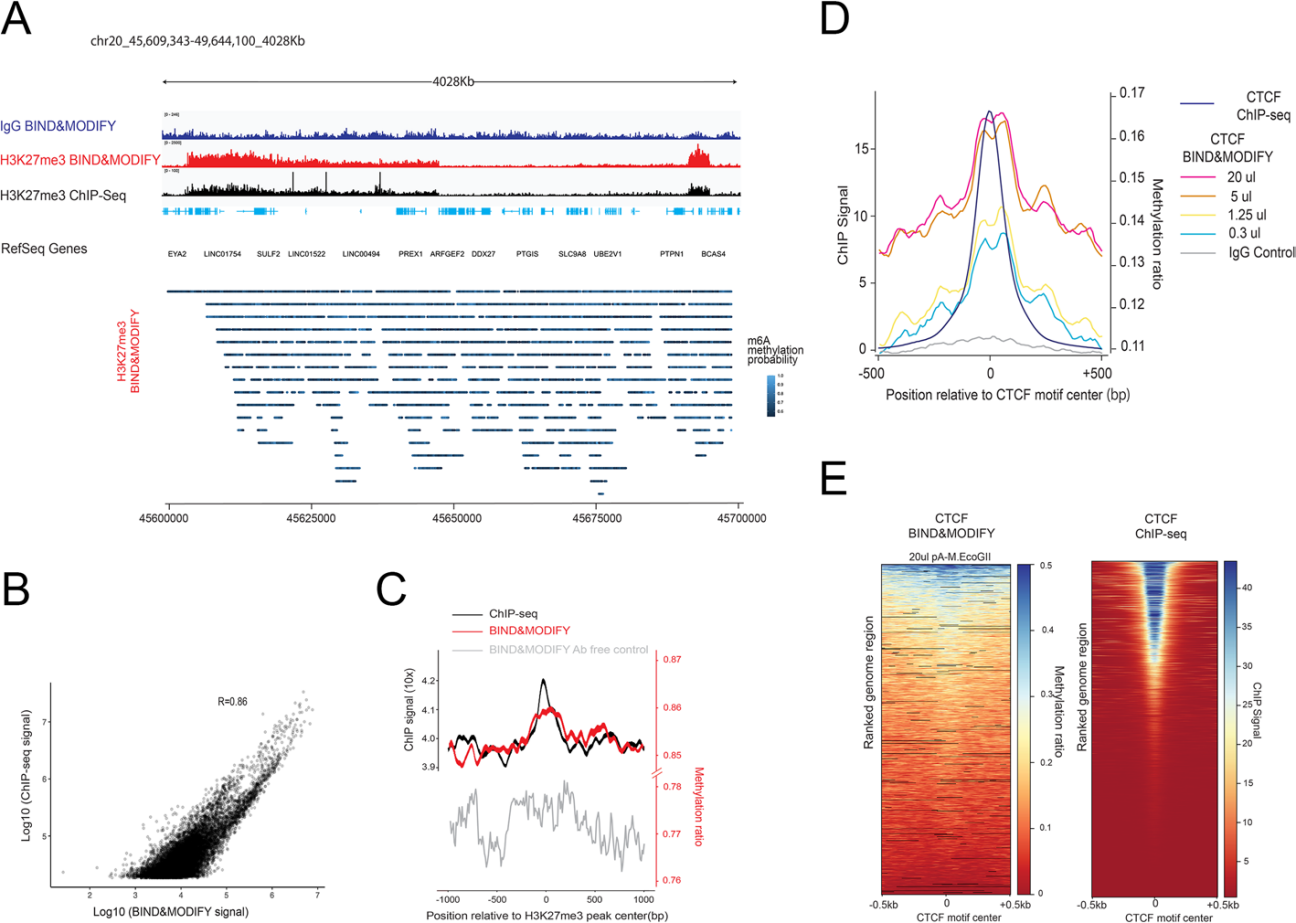
**The consistency of H3K27me3 pattern between ChIP-seq and BIND&MODIFY in situ**.** A** The nanopore data were aligned to the reference genome (mouse: MM10, human: HG19), and the methylation sites were called by our house pipeline based on the m^6^A methylation probability cut-off 0.53. Top panel: The H3K27me3 signal (BIND&MODIFY signal on the *y*-axis: the number of called m6A methylation sites in 100 bp bin; ChIP-seq signal on the *y*-axis: the read counts in 100 bp bin), by BIND&MODIFY with H3K27me3 /IgG antibody and ChIP-seq in genome scale view. Bottom panel: single-molecule reads with m6A probability. Each point on reads represented the adenosine and the color indicated the methylation probability. **B** The scatter plot of BIND&MODIFY signal (m6A counts) and ChIP-seq signal (read counts) in peak regions (the signal was normalized based on region length). **C** The H3K27me3 peak regions were called by MACS in ChIP-seq. Then the middles of all peak regions were centered at 0. The upstream/downstream 1000bps were plotted around the peak center 0 with sliding 100-bp bins. BIND&MODIFY *y*-axis indicated the mean methylation ratio in 100-bp bins, and ChIP-seq signal indicated the mean read counts. The antibody free control with untethered pA-M.EcoGII marked the chromatin accessibility. The signal of IgG control was low (not shown). **D** The effect of pA-M.EcoGII doses on CTCF patterns of BIND& MODIFY. Four doses of pA-M.EcoGII, (0.3, 1.25, 5, and 20 µl) were applied on 4T1 cell lines followed by BIND&MODIFY method, and mean m^6^A methylation ratio was plotted by + 500 bp/ − 500 bp of CTCF motif center (similar to panel **C**). The signal of IgG control was the lowest. **E** The genome region ranking of m^6^A methylation ratio and ChIP signal for CTCF motif center on each genome region of panel **D**. Mean m6A methylation ratios and ChIP signal (sliding 100-bp bins) were plotted around + 500 bp/ − 500 bp of CTCF motif center for each gene of 20 µl dose of pA-M.EcoGII and ChIP-seq. Color bar indicated mean methylation ratio. Each row indicated one genome region with CTCF motif center of one certain gene

For the initial QC, the average read length of the Nanopore outputs was approximately 2 kb (Additional file 1: Fig. S3a), and the correlation of the experimental replicates was 0.90 (Additional file 1: Fig. S3b). Both the conventional method (ChIP-seq) and the proposed BIND&MODIFY method showed similar H3K27me3 signals across various genome scales (Fig. 2a, Additional file 1: Fig. S4). Analysis of the regional signal strength revealed that the signal strength of m^6^A counts observed with BIND&MODIFY correlated with the ChIP-seq signal intensity (Fig. 2b). 60 ~ 80% of the position signals observed in ChIP-seq were also observed with BIND&MODIFY (Additional file 1: Fig. S3cd). To profile the enrichment of H3K27me3 within genomic regions, enrichment data were visualized using H3K27me3 peak-centered signal plots. Overall, the BIND&MODIFY results were consistent with those of ChIP-seq in H3K27me3 (Fig. 2c). Due to the H3K27me3 broad signal, we also selected another active histone marker H3K4me3 as example, which showed the sharp peaks in ChIP-seq. Similarly, the BIND&MODIFY showed the comparable signal on the peak regions of H3K4me3 (Additional file 1: Fig. S11ab). Collectively, the BIND&MODIFY results were comparable to those of conventional ChIP-seq on histone markers.

As a critical regulator of genome organization, the CCCTC-binding factor (CTCF) is a DNA-binding protein that plays an essential role in maintaining the topological structure of chromatin and induction of gene expression [33]. To determine whether our current protocol may exert dose-specific effects on probing the DNA-binding protein CTCF, we assessed the dose-specific effects of BIND&MODIFY. CTCF antibody was first applied to a 5 × 10^5^ mouse mammary gland 4T1 cell line, and different doses of pA-M.EcoGII (0.3 µl, 1.25 µl, 5.0 µl, 20 µl) were used for tethering with individual samples, followed by the performance of BIND&MODIFY. We found that the doses of 20.0 and 5.0 µl showed similar CTCF motif-centered analysis, though lower doses of 1.25 and 0.3 µl showed a lower m^6^A ratio (Fig. 2d, Additional file 1: Fig. S12). The CTCF motif-centered gene analysis showed a signal identical to that of conventional ChIP-seq (Fig. 2d,e). Our results indicated that the BIND&MODIFY protocol (20 µl pA-M.EcoGII) is optimal for probing sensitive DNA-binding protein transcription factors, such as CTCF. We further tested the sequencing depth effect on the BIND&MODIFY detection. Over 2 × sequencing depth could generate the reliable CTCF signals (Additional file 1: Fig. S13a-k). BIND&MODIFY, identifying the heterogeneity of the CTCF binding, showed the 5 different binding pattern of CTCF (Additional file 1: Fig. S13l).

We tested multiple experimental variables to optimize the best protocol of CTCF targeting BIND&MODIFY (Additional file 1: Fig. S19). Firstly, we introduced the spermidine to 0.05 mM in the activation buffer for all the conditions. Then we tested adding DNA crowing reagents Glycogen 1.5 µg/µl or PEG-6000 0.83% in the activation buffer, and they showed no significantly better CTCF peak distribution. Further, we increased the incubation time from 30 to 60 min and 120 min and found 120 min showed the best signal–noise ratio for CTCF peak distribution. Also, we added the primer-annealed dsDNA (1, 10, and 100 ng/µl) to absorb the free-floating pA-M.EcoGII, and found dsDNA 1 ng/µl was sufficient to give the best signal–noise ratio while maintaining minimal influence on the IgG background peak elevation. The sequencing depth for this experiment was about 1.0X for all the conditions tested. In this case, we found the best condition for the BIND&MODIFY reaction targeting CTCF, referred as Protocol V2. Some helped improve the methylation efficiency, but none fundamentally changed the results and conclusions from previous experiments by Protocol V1. We have published the most up-to-date version of the detailed experimental protocol, as Protocol V2, and can be accessed at Protocol.io with the following doi: (https://dx.doi.org/10.17504/protocols.io.3byl4j7m8lo5/v1).

We carried out additional experiments to access the pA-M.EcoGII’s preferences for tagging DNA-binding protein CTCF at open chromatin and histone modification H3K9me3/H3K27me3 at heterochromatin non-accessible regions. Inspired by classical in situ Hi-C protocol [34, 35], we suppose SDS could open heterochromatin and make the pA-EcoGII to the non-accessible regions. We found that the 0.1% SDS could denature the heterochromatin and make them accessible for M.EcoGII (Additional file 1: Fig. S20). Then we added the 0.1% SDS to the BIND&MODIFY experiment protocol prior to antibody binding. We found the SDS did not affect the CTCF labeling efficiency (data not shown) due to the prior fixation. However, the SDS increased the methylation peak value of heterochromatin labeling by about 10% (data not shown). Both results supported that the SDS denaturing process could decondense heterochromatin.

In conclusion, the BIND&MODIFY method showed comparable ability as conventional methods to map histone modifications and DNA-binding proteins.

### Long-range sequencing resolves epigenetic modification in complex genomic regions and phases the epigenome

**Fig. 3:**
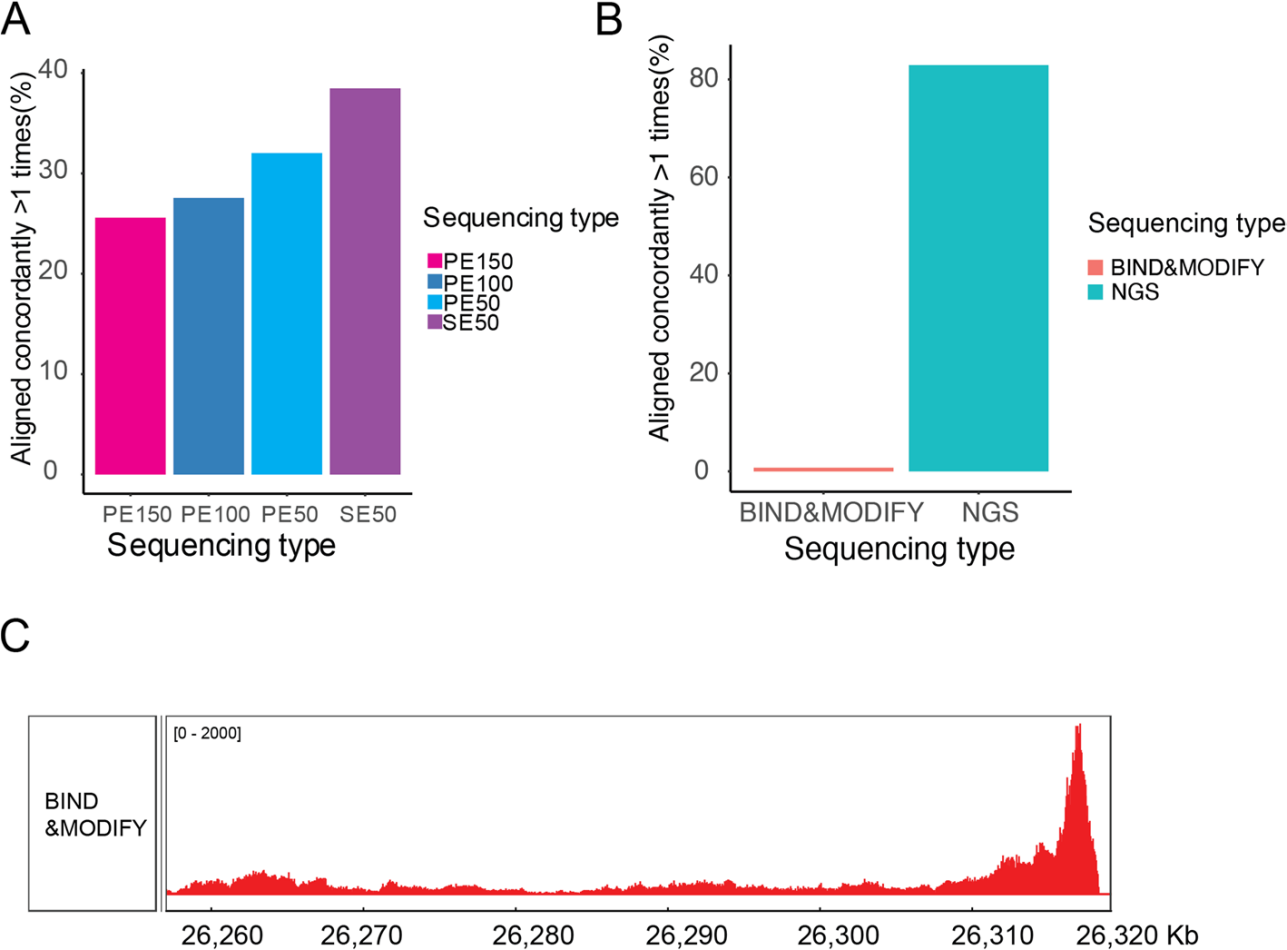
**The BIND&MODIFY disclose the H3K27me3 distribution around pericentromeres**.** A** We simulated the reads with different length based on the pericentromeric regions (PE50-paired end sequencing 50 bp, SE50-single end sequencing 50 bp) (Methods). The pericentromeric region is defined as the boundary regions of the centromeres which could be labeled by H3K27me3 (The center areas of centromeres cannot be labeled; reference genome: CHM13). The multiple alignment rates decreased with the increasing read length. The PE150 still have 25% multiple alignments in the pericentromeric regions. **B** In the real sequencing data, the SE50 demonstrated higher multiple alignment rates than the simulated SE50, due to the randomness size distribution. The BIND&MODIFY have the much lower multiple alignment rate, which could offer the more accurate epigenomic locations. **C** The H3K27me3 signal distribution on the pericentromeric regions (Chr20:26,260,000 ~ 26,320,000). The H3K27me3 signal increased to the highest on the boundary region of the centromere

The centromere and pericentromere are typically established on highly repetitive DNA arrays [36]. In our sequencing simulation of the pericentromere, the NGS showed 20–40% multiple alignments on average, and the longer reads (PE150) generated 25% multiple alignments (Fig. 3a). Multiple alignments might disturb the ChIP-seq signal and bias the detection accuracy. In the real sequencing data, we found that the nanopore long-range sequencing (length > 2 kb) showed a 40 × lower multiple alignment frequency (2%) than the NGS SE50 (80%) (Fig. 3b). Accurate alignment could help identify the histone modification H3K27me3 on the pericentromere. The results of BIND&MODIFY showed that H3K27me3, which is a gene suppressing marker that facilitates heterochromatin formation, was present along the boundary of the centromere with the missing H3K27me3 in the centromere core (Fig. 3c). This finding might help explain the transition from euchromatin to heterochromatin around the pericentromeric regions. Retrotransposons are other regions composed of repeated sequences. We visualized the H3K27me3 status on LTRs, SINEs, and LINEs using retrotransposon-centered plots by including a 300-bp region upstream/downstream of similarly sized retrotransposons (Additional file 1: Fig. S6). BIND&MODIFY also improved the signal of these complex retrotransposon regions.

Another advantage of long-range sequencing, in addition to alignment on complex genomic regions, is the ability to phase the genome, which offers the opportunity to study the differences in the epigenetic regulations of paternal and maternal genomes. Thirty percent of these reads were phased into two genomes. Over 50% of single-nucleotide variants (SNVs) could be phased in this study (Additional file 1: Fig. S7). By tracing the phased SNVs, the H1/H2 genomes showed a different pattern of H3K27me3 distribution and coverage bias. However, owing to insufficient sequencing depth and the lack of completed paternal and maternal genomes, further detailed studies are required to draw conclusions.

In summary, BIND&MODIFY could precisely map histone modifications and protein interactions in challenging complex genome regions and phase the genome.

### Epigenomic status at single-molecule resolution

Recent single-molecule and single-cell measurements of histone accessibility suggest that ATAC-seq of cell populations generates an ensemble average of distinct nucleosome states [37]. An essential attribute of BIND&MODIFY involves the measurement of histone modification at single-molecule resolution by taking advantage of this slight variance and the cumulative probability in segments (Additional file 1: Fig. S5).

**Fig. 4:**
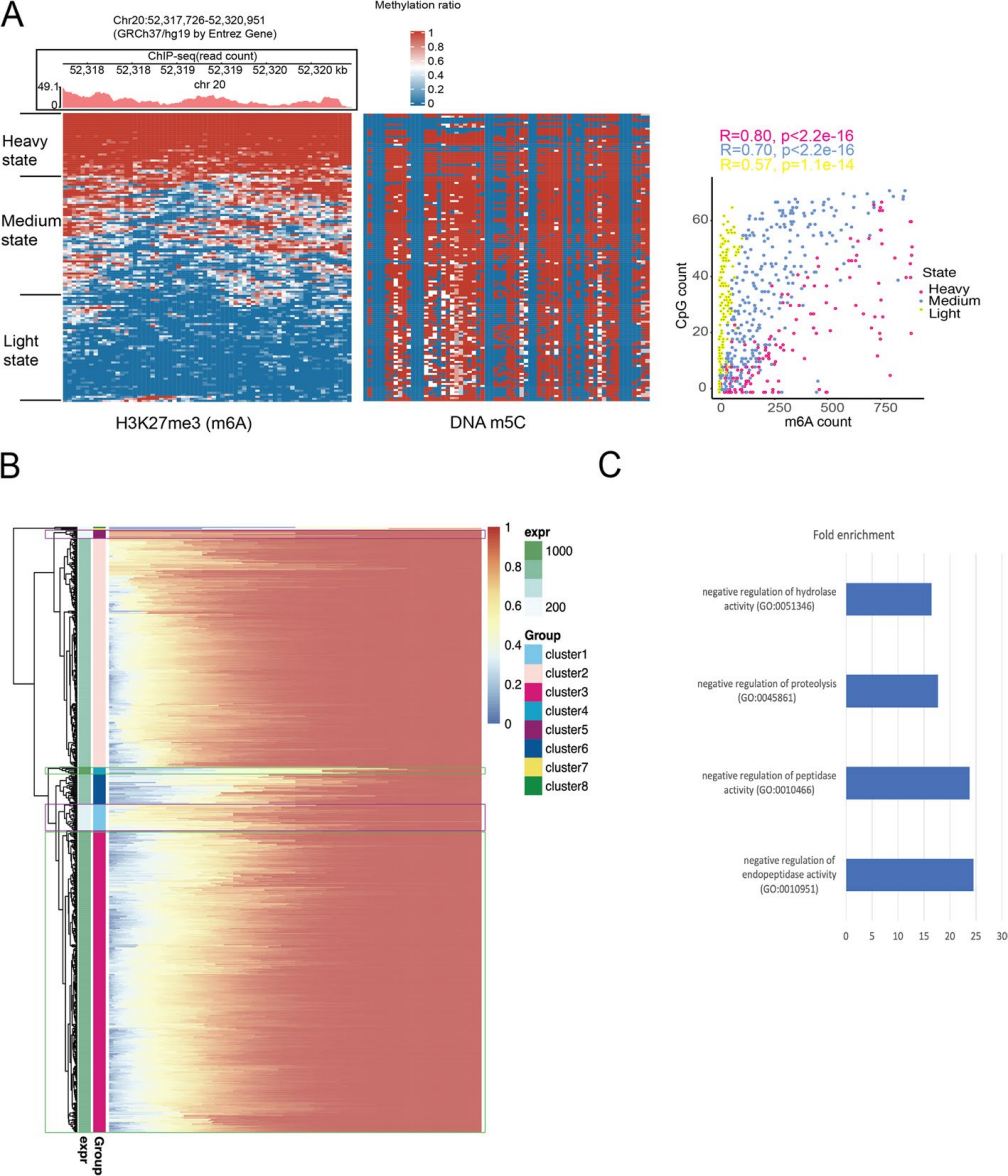
**The BIND&MODIFY showed the heterogeneity of H3K27me3 regulation**.** A** The read counts of H3K27me3 in ChIP-seq (Chr20:52,317,726–52,320,951) was shown in upper panel. The single-molecular resolution of Chr20:52,317,726–52,320,951visualize each molecular methylation statues of H3K27me3. The rows in heatmap represented the different DNA molecules. The color indicated the methylation ratio (non-sliding 50-bp bins), which represented the possible H3K27me3 signal. The DNA molecules could be classified into three states: heavy state (methyl) (We used the m.^6^A methylation probability cut-off 0.80 in the single-molecular analysis; the methylated bins were defined as the methylation ratio > 0.5 in bins and at least 2 methylation sites; heavy states were defined as the methylation bin density > 75%), medium state (defined as the methylation bin density 25 ~ 75%), and light state (defined as the methylation bin density < 25%). The right heatmap showed the CpG methylation distribution on the corresponding DNA molecules in the left H3K27me3 heatmap (one-to-one matching). The H3K27me3 signal decreased with the decreasing CpG methylation (scatter plot). **B** H3K27me3 heterogeneity pattern of genetic promoters in Chr20 (method in Fig. S13). Each pixel on each row corresponds to methylation bin density of each individual DNA molecule, and each row corresponds to each genetic promoter (2 kb upstream of the transcription start site included most of the promoter regions). The methylation bin density was ranked from low to high (left to right). The blue, white-yellow, red corresponded matched the heavy, medium, light state. The left green bar (expr) showed the average expression value (expected count) of the corresponding gene clusters. Cluster 3, Cluster 4 with high RNA expression level were highlighted by green box. Cluster 1, Cluster 5 with low RNA expression level were highlighted by purple box. **C** The bar plot showed the GO enrichment analysis of the Cluster 1. Details of H3K27me3 heterogeneity calculation methods can be found in Fig. S18

Next, we aimed to determine whether BIND&MODIFY could reveal the differences in the H3K27me3 status by investigating the Chr20: 52317726–52320951 loci. The Chr20:52317726–52320951 loci are located with the long intergenic non-protein coding RNA LINC01524, a prognostic biomarker and an enhanced factor of cancer [38, 39]. Conventional ChIP-seq enriched the H3K27me3-bound motif by antibody-guided amplification, which can lead to overrepresentation (Fig. 4a). In contrast, BIND&MODIFY presented a comprehensive picture of H3K27me3 distribution in this region. Based on methylation bin density of individual DNA fiber, we classified single-molecule DNA into three distinct epigenetic states (Fig. 4a): the heavy state with methylation bin density > 75%, medium state with methylation bin density 25–75%, and light state with methylation bin density < 25%. To further explore whether the observed epigenomic states could be attributed to the labeling efficiency of BIND&MODIFY, we examined whether the H3K27me3 states were in accordance with DNA CpG methylation, which was reported to correlate with H3K27me3 for the silencing of gene expression [40]. As expected, in most of the molecules, H3K27me3 and CpG methylation changed concordantly (Fig. 4a, Additional file 1: Fig. S8). The artificially labeled m^6^A did not significantly affect the detection efficiency of 5mC, and the correlation of 5mC with bisulfite sequencing was 0.8 (Additional file 1: Fig. S10). The signal peaks (chr20: 52225000–52225500) in ChIP-seq summed up the H3K27me3 loci in heavy and medium states. Furthermore, we presented the mean methylation bin density of each sequencing read (each DNA molecule in the promoter region) as a point on the heatmap (Fig. 4b), and the heterogeneous states of the promoter regions were visualized with the color gradient horizontal lines in the heatmap (Fig. 4b). We then obtained a “big-picture” view of global promoters in Chr20, summarizing a more comprehensive perspective with specific examples and details. A previous study showed that H3K27me3 typically suppressed gene expression [41]. The BIND&MODIFY also showed a similar pattern, with fewer heavy/medium (red/yellow) H3K27me3 states leading to higher gene expression (Cluster 3, Cluster 4, highlighted in green box). The promoter regions with more heavy/medium H3K27me3-modified molecules always showed low gene expression (Cluster 1, Cluster 5, highlighted in purple box). Through gene ontology analysis, we investigated H3K27me3-suppressed gene regions (Cluster 1). These genes function as negative regulators of hydrolase, proteolysis, peptidase, and endopeptidase activity and play key roles in cell proliferation, angiogenesis, and tumor invasiveness by regulating extracellular matrix degradation, cell signaling, and recognition [42, 43] (Fig. 4c). Another active histone marker H3K4me3 might show the close relationship with the gene expression. The H3K4me3 BIND&MODIFY signal showed similar peak distribution compared to ChIP-seq (Additional file 1: Fig. S11a-b). In contrast to H3K27me3, the cluster with the highest H3K4me3 frequency leads to the highest gene expression (Additional file 1: Fig. S11c-d). The gene ontology analysis showed that these genes were involved in the biological process of protein secretion, G-protein coupled pathways, vesicle transport, and cell migration (Additional file 1: Fig. S11e).

We also investigated the single-molecular conditions of CTCF (Additional file 1: Fig. S15). By further studying the CTCF activity in the promoter regions, we found that the extreme CTCF binding status (extremely heavy Cluster 2 or extremely light Cluster 6) resulted in lower gene expression. In contrast, the medium CTCF binding state promoted gene expression (Cluster 1).

### BIND&MODIFY revealed the long-distance correlation between regulators

**Fig. 5:**
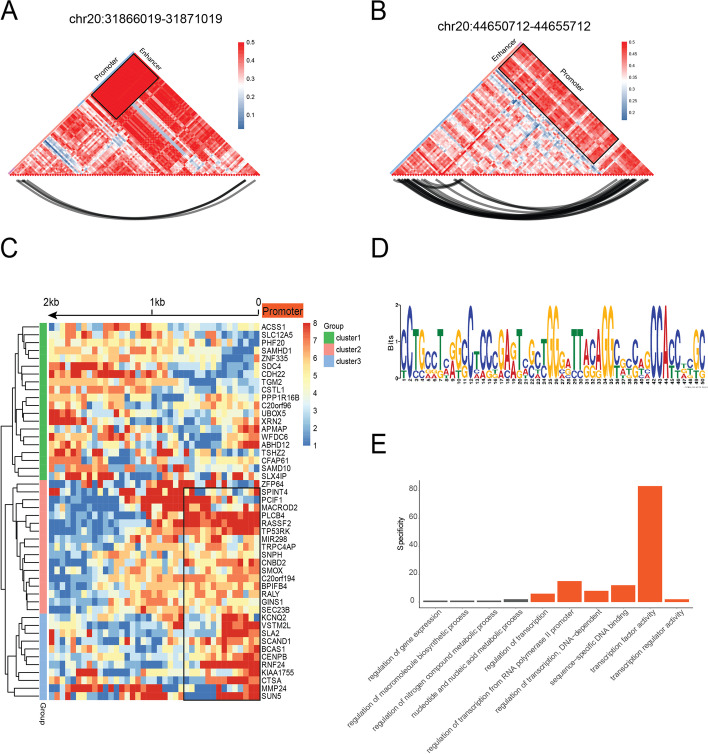
**The long-distance H3K27me3 correlation between cis-regulators and promoters**.** A**, **B** We selected two genomic regions (chr20:31,866,019 ~ 31,871,019 gene *Dsup15*, chr20:44,650,712 ~ 44,655,712 gene *Ada*), which included the documented promoters and enhancers. Each pixel in the heatmap means the 50 bp length. The correlation index calculated based on method. The higher correlation index (red) between two regions suggested the accordant changes. We could observe the high correlation (black square) between the enhancers and promoters. The lower black curves indicated the interaction signals in Hi-C data. The physically interacted regions (Hi-C) also supported the high H3K27me3 correlation. **C** We further analyzed the promoter upstream 2-kb regions to see whether there were some strongly correlated genomic areas, the potential cis-regulators for the promoters. The calculation details could be found on the method. Each pixel in the heatmap was 50 bp length. Some prompters strongly correlated with their proximal regions (black box), suggesting that these proximal regions synchronized with the corresponding promoters in the H3K27me3 regulation. **D**, **E** By further analyzing the binding motif of these proximal regions, we found these regions have the binding motifs for the transcription regulators, modulating the transcription activity

During the transition from the heavy state to the light state of H3K27me3, we found that regional H3K27me3 maintained the same status (methylated/unmethylated) in most molecules. The rhythmic manner may disclose the synergistic effects between cis-regulators on the genome. For instance, the enhancer and promoter were looped by CTCF and cohesin, and the close loci might share rhythmic histone regulation. We developed a modified co-labeling coefficient (CC) metric that assessed the degree of H3K27me3 methylation correlation between genomic regions (Fig. 5a,b). To study interactions at a considerable range, we inspected several promoters and enhancers with sufficiently covered reads. In the chr20: 31866019–31871019 region, the H3K27me3 methylation on the enhancer was synchronized with the corresponding promoters, suggesting the presence of a synergistic regulator switch between enhancer and promoters (Fig. 5a). The other three sets of promoters and enhancers also exhibited a similar synergetic phenomenon (Fig. 5b). We also detected the physical interactions between promoters and enhancers in Hi-C data, further supporting the rationality of the rhythmic manner of the cis-regulators (Fig. 5b). Overall, the CC analysis of gene elements supported the co-regulation between the promoters and these upstream enhancers. Considering the hypothesis of cis-regulating synergy, we might find more potential cis-regulators that synchronize with the promoters.

To explore the potential cis-regulators globally, we summarized the promoter-related correlations in the upstream 2 kb genomic region (Fig. 5c). The horizontal locations of the heatmap indicated that these genomic loci were located upstream of the promoters, and the color indicated the correlation intensity. We then plotted the CC index of all regions 2 kb upstream of the promoters on Chr20 (Fig. 5c). We observed 48 genetic regions with strong correlations with their promoters. These potential cis-regulators were grouped into two patterns: proximal cis-regulators [27] and distant cis-regulators [21]. The motif analysis of the proximal cis-regulators showed the binding sites of the transcription regulators, such as MED1, SOX10, and EDF1 (Fig. 5d,e). In contrast, motif analysis of the distant cis-regulators showed the binding sites for splicing factors, such as SRSF3 and U2AF2 (Additional file 1: Fig. S9).

We also found a CTCF correlation between the enhancers and promoters in genomic regions (chr20:23,030,309–23,035,309, chr20:32,274,191–32,279,191). CTCF and cohesin recruit enhancers and promoters to promote transcription [44]. Therefore, the high correlation between CTCF and cis-regulators might be attributed to the CTCF-mediated adhesion. We used BIND&MODIFY to find more potential cis-regulators (Additional file 1: Fig. S16). We found that 40% of potential cis-regulators (cis-regulators for PHF20, CDH22, APMAP, SMOX, SEC23B, and RNF24) were also detected in the H3K27me3 correlation analysis. These results further supported the synergetic regulation among these cis-regulators.

Overall, the BIND&MODIFY method successfully demonstrated long-range or short-range synchronized interactions of the modified histones between cis-regulators.

## Discussion

ChIP-seq and CUT&TAG have been used as essential epigenetic study tools for many years, especially for analyzing histone modification and transcription factor binding, among other processes. The conventional CUT&TAG and ChIP-seq methods are based on the calculation of the peak of the enriched reads in a specific region [45]. ChIP-seq, CUT&RUN, and CUT&TAG protocols can detect protein–DNA binding events and chemical modifications of histone proteins, respectively [38–40]. However, a limitation of these methods is that their signals represent collective immunoprecipitation-enriched DNA fragments without considering the heterogeneity of DNA molecules. The use of short-read sequences in these methods also prevents the analysis of long-range interactions observed in multi-omics epigenome data. We designed a new method, BIND&MODIFY, using protein A-M.EcoGII methyltransferase recombinant proteins for non-fragmented labeling of local DNA regions. Using this method, we simultaneously profiled the multi-epigenome information on a truly unbiased genome-wide scale, measured the distribution of histone modification at a single-molecule resolution, and identified the loci exhibiting a significant correlation. However, we would like to discuss several technical drawbacks of this novel technology, which could be addressed in the future.

Base calling is the first dimension that needs improvement in nanopore sequencing. PacBio sequencing with high accuracy and detection sensitivity could help this base calling issue further, but the sequencing throughput is lower than that of the nanopore. In this study, using 50–100-bp sliding windows to add enough m^6^A to the signal, we could increase the accuracy by compromising the resolution. If a higher resolution is required, the base calling accuracy needs to be improved. In this study, we also attempted to call native 5mC and artificially labeled m^6^A simultaneously to analyze the relationship between histone modification and DNA methylation. The simultaneous calling of 5mC and abundant m^6^A has also been reported previously [32, 46]. Alternatively, PacBio, with little interference from the neighboring nucleotide signal, can also be used. Overall, the simultaneous detection of 5mC and the regulator position at the single-molecule level is possible using BIND&MODIFY.

Endogenous methylation in mammalian genomes is a potential confounding factor in our analysis. On examining non-treated original genomic DNA data, we found that the quantity of m^6^A in the original DNA was more than a thousand-fold lower than that in the treated samples, suggesting a minimal effect of endogenous methylation. However, in some species, m^6^A occurs endogenously and is strongly correlated with the binding motif of the modified histone. Modifications such as 5mC, 4mC, cytidine deamination, and 5-glucomethyaltion are potential alternatives for such cases. In addition, some artificial SAMs that could introduce biotin to specific sites may have avoided the endogenously confounding signals.

The second shortcoming of BIND&MODIFY was the 2 kb average read length, which is slightly shorter than that of conventional nanopore genome sequencing. This issue was also observed with another M. EcoGII-labeling technology, SMAC-seq [32]. We hypothesized that the sucrose and spermidine in the reaction improve the EcoGII activity and that sucrose decreases the genomic DNA solubility, thereby affecting DNA extraction. We believe that the reaction buffer needs further adjustment to balance the M.EcoGII activity and genome solubility and obtain better sequencing results.

The BIND&MODIFY method identified correlated epigenetic loci with the widely used ChIP-seq. Despite the general similarity between the two methods, relatively small signals were observed in terms of the variable peak signal strength. This could be attributed to the differences in the fundamental principles and throughput between BIND&MODIFY and conventional ChIP-seq. The ChIP-seq method amplifies the loci signals that are common across most DNA molecules by immunoprecipitation enrichment but neglects the non-signal loci in individual DNA molecules. The BIND&MODIFY method enables unbiased profiling of the DNA molecule without immunoprecipitation enrichment and reflects the true nature of the epigenome, thereby requiring a much higher sequencing depth. Fortunately, the nanopore sequencing throughput is increasing rapidly, and selective target enrichment methods are also becoming available for nanopore sequencing [45, 47, 48]. However, the further bioinformatics were needed to develop the signal identification algorithms, including background noise reduction and peak calling.

The BIND&MODIFY uses an unbiased un-amplified approach to target DNA–protein interaction at specific loci, which may result in differences in peak size and signal-to-noise ratio when targeting different epigenomic markers. For the histone modification marker H3K27me3, which usually formed continuous broad blocks ranging from 1 to 10 kb, the BIND&MODIFY showed relatively broad peaks. This may be due to H3K27me3 flanking region from adjacent ultra-wide blocks. For the histone modification marker H3K4me3 and transcription factor CTCF, the BIND&MODIFY showed much sharper peaks and resulted in better signal-to-noise ratio.

Due to the highly packed heterochromatin which is not very accessible for the enzyme, we supposed the BIND&MODIFY could be biased to more accessible euchromatin. Originally, M.EcoGII was discovered to be a novel method of soft labeling chromatin accessibility at a single molecule level [32, 49, 50]. The ability to label accessible chromatin is the crucial feature of M.EcoGII. We suppose that the M.EcoGII and other enzymes, such as Tn5, have more chances of attacking the accessible region, which is biochemically reasonable. Similar bias towards accessible chromatin regions was found in DiMeLo-seq [51] and Cut&Tag [52]. Our results of 0.01% SDS treatment before pA-M.EcoGII also could denature the heterochromatin and make them accessible for M.EcoGII but not affect the CTCF labeling efficiency. But SDS treatment did not solve all the problems. Based on the experience from other labs, the optimized permeabilization and fixation may also help to resolve this problem. Also, the BIND&MODIFY, not incorporating the PCR amplification, required the high DNA input amount (5 × 10^5^cells and > 1ug DNA).

Owing to this new technology, we could only conservatively interpret the biological meaning of heterogeneity and long-range correlation. Surprisingly, genetic regions with high CTCF heterogeneity were associated with high RNA expression. The heterogeneity possibly suggested a highly dynamic nature of these genome regions and might support the “pulsing” transcription model, in which transcription fluctuates between on/off modes. The high dynamics might suggest a high frequency of the transcription on/off switch. Another possibility is that the high dynamics may be due to the cell cycles. In contrast, the range correlation could suggest the synergetic regulations among cis-regulatory elements (CREs) or the close spatial distance. The two cis-regulators with close spatial distance (3D for distant loci or 2D for close loci) could be labeled by the same BIND&MODIFY enzyme in the same spatial location. However, we investigated this possibility and found the enzyme did not efficiently label the spatially close loci (Additional file 1: Fig. S17). In another possibility, the close CREs may be under the same biological regulations, sharing the same epigenetic markers. However, in the single-molecular resolution analysis, we could not reasonably exclude the possibility of the different labeling efficiency in each molecule. Therefore, the further studies were needed to confirm our results.

Finally, we believe that the integration of BIND&MODIFY into a single-molecule multi-omics assay is likely to be an effective intervention in the near future. Using this method, we can simultaneously label two or more proteins bound to the same single DNA molecule using different modification labels. Then, the protein interaction distance on the genome can be measured, which may provide a breakthrough in understanding protein–protein interactions at the whole genome scale. In principle, similar approaches can also be applied to individual RNA molecules to study RNA-binding proteins. BIND&MODIFY also allows the footprinting of other transcription factors. We expect BIND&MODIFY to provide new insights into the regulatory dynamics of genomes across various experimental systems and sequencing platforms in the future. We also expect this technology to become a part of a new class of tools for multi-epigenomics.

## Conclusions

In summary, BIND&MODIFY is a powerful long-range method for investigating histone modifications and DNA-binding proteins at the single molecule level. Our approach shows comparable ability as conventional bulk ChIP-seq and CUT&TAG, extends the DNA–protein interaction loci profiling to multiple kilobases in length even in challenging complex genome regions, and detects the histone modification and endogenous 5mC methylation with the haplotype-specific pattern on the single long DNA fiber. Notably, no DNA amplification is needed for BIND&MODIFY, single-molecular heterogeneity and long-range interactions of histone modification, DNA–protein interaction, and 5mC methylation can be jointly measured on the same single-molecule DNA. The ability to measure the absolute frequency of epigenetic modification and DNA–protein binding at specific loci in the whole genome level makes our current implementation of BIND&MODIFY an attractive single-molecular genomics-based strategy for broad range of developmental and pathogenesis of disease studies.

## Methods

### Cell culture and antibodies

Human mammary gland carcinoma cell line MCF-7, mouse mammary gland carcinoma cell line 4T1, and Human cervix adenocarcinoma cell line HeLa were obtained from ATCC. MCF-7 were grown in DMEM (Gibco,11995065) supplemented with 10% FBS (Gibco,10099141), 0.01 mg/ml insulin (HY-P1156, MedChemExpress), and 1% penicillin–streptomycin (Gibco, 15140122). 4T1 were grown in RPMI1640 (Gibco, 11875093) supplemented with 10% FBS (Gibco,10099141), and 1% penicillin–streptomycin (Gibco, 15140122). HeLa were grown in DMEM (Gibco,11995065) supplemented with 10% FBS (Gibco,10099141) and 1% penicillin–streptomycin (Gibco, 15140122). Cell lines were regularly checked for mycoplasma infection (Yeasen, 40612ES25) and confirmed negative. We used the following antibodies: H3K27me3 (CST, 9733, Lot19), CTCF (Millipore, 07–729-25UL), H3K4me3 (GenScript A2357), 5mC (Abcam, ab214727), Protein A Antibody, pAb, Chicken (A00729-40, GenScript), Guinea Pig anti-Rabbit IgG (Heavy & Light Chain) antibody (Antibody online, ABIN101961).

## Preparation of recombinant protein pA-M.EcoGII

pTXB1 vector (NEB, N6707S) was used as the protein expression backbone. Downstream of the lac operator, a ribosome binding site and three FLAG epitope tags were introduced, followed by two IgG-binding domains of staphylococcal protein A encoding sequence, which was synthesized based on the previous work (Addgene, 124,601). The M.EcoGII encoding sequence (Addgene, 122,082) was also synthesized based on its original construct. The amino acid linker sequence between the C-terminus of protein A and N-terminus of M.EcoGII is DDDKEF. The sequenced plasmid was transformed into C3013 competent cells (NEB) following the manufacturer’s protocol. Each colony tested was inoculated into a 1-mL LB medium, and growth was continued at 37 °C for 2 h. That culture was used to start a 50-mL culture in 100 μg/mL carbenicillin containing LB medium and incubated on a shaker until the cell density reached an A600 of 0.6, whereupon it was chilled on ice for 30 min. Fresh IPTG was added to 0.25 mM to induce expression. Then the culture was incubated at 21 °C on a shaker for 16 h. The culture was then collected by centrifugation at 10,000 rpm, 4 °C for 30 min, and the supernatant was discarded. The bacterial pellet was frozen in a dry ice-ethanol bath and stored at − 80 °C. The frozen pellet was resuspended in 20 mL chilled HEGX Buffer (20 mM HEPES–KOH at pH 7.2, 0.8 M NaCl, 1 mM EDTA, 10% glycerol, 0.2% Triton X-100) including 1 × Roche Complete EDTA-free protease inhibitor tablets and lysed using a high-pressure cell disrupter (JNBIO, China). Cell debris was removed by centrifugation at 10,000 rpm for 30 min at 4 °C, and the supernatant was loaded onto a column equipped with chitin slurry resin (NEB, S6651S), then incubated the column on a rotator at 4 °C overnight. The unbound soluble fraction was drained, and the columns were rinsed with 20 mL HEGX containing Roche Complete EDTA-free protease inhibitor tablets. The chitin slurry was transferred to a 15-mL conical tube and resuspended in Elution buffer (10 mL HEGX with 100 mM DTT). The tube was placed on the rotator at 4 °C for ~ 48 h. The eluate was collected and concentrated using an Amicon Ultra-4 Centrifugal Filter Units 10 K (Millipore, UFC801096), and sterile glycerol was added to make a final 50% glycerol stock of the purified protein. The recombinant protein was stored at − 80 °C. pA-M.EcoGII expression plasmid is available from Addgene (pTXB1-pA-M.EcoGII; Addgene,192873).

### Size characterization of recombinant protein pA-M.EcoGII

The size of purified pA-M.EcoGII recombinant protein was characterized by Coomassie blue staining by resolving the protein in 7.5% SDS-PAGE gel.

### ELISA of recombinant protein pA-M.EcoGII

To verify the binding efficiency of pA-M.EcoGII, we performed an ELISA assay in vitro. The purified recombinant protein or commercial M.EcoGII (NEB, M0603S) was diluted with coating buffer (0.05 M NaHCO_3_ buffer, pH 9.2). The 96-well high binding plate (Greiner Bio-one, 655,061) was coated with 100 μL pA-M.EcoGII or commercial M.EcoGII (NEB,M0603S) (1:120, 1:480 of stock 10 mg/ml), negative control, commercial Protein A (Thermo Fisher, 21,181) (1:120, 1:480 of stock 10 mg/ml) in coating buffer per well for 4 h at room temperature. Further, 200 μL SuperBlock™ (TBS) Blocking Buffer Blotting (Invitrogen, 37,537) was added to each well and incubated for 2 h at room temperature. Then each well was washed 5 × with 380 μL washing buffer (0.14 M NaCl; 0.01 M PO_4_;0.05% Tween 20; pH 7.4). After that, 95 μL Secondary Antibody (1:10,000 in PBSB) was added to each well and incubated for 1 h at room temperature. The plate was washed 4 × with 380 μL washing buffer (0.14 M NaCl; 0.01 M PO_4_;0.05% Tween 20; pH 7.4). Ninety microliters TMB substrate solution was added and incubated for 15 min at room temperature in the dark. Finally, 90 μL stop buffer (1.8N H_2_SO_4_) was added to stop the color development and read immediately at 450 nm using FLUOstar® Omega Plate Reader by BMG LABTECH.

### In vitro methtransferase activity of pA-M.EcoGII recombinant protein

To access the in vitro methylation efficiency of pA-M.EcoGII recombinant protein, m^6^A methylation-sensitive restriction enzyme DpnI was used to probe the adenine methylation at GATC motif of 7-kb unmethylated dsDNA. The 7-kb dsDNA substrate was PCR amplified from lambda DNA. For the methyltransferase reactions, each 50 μl reaction volume was assembled on ice and contained 1 μg 7 kb unmethylated dsDNA, 1X Cutsmart buffer, 640 μM SAM, and 4 μL of pA-M.EcoGII recombinant protein or commercial M. EcoGII, then the mixture was incubated at 37 °C for 1 h. The methylated product was purified using 0.6X Ampure XP (BECKMAN COULTER, A63882). One microliter of DpnI (NEB) was added to the reaction mixture to further incubate at 37° for 10 min. DpnI cutting efficiency was examined by 1% agarose gel electrophoresis.

To assess the specificity of pA-M.EcoGII recombinant protein methylation and its effectiveness at inhibiting restriction endonucleases, we carried out restriction analyses using an unmethylated 7-kb linear dsDNA template, which was PCR amplified from pTXB1. One enzyme known to be insensitive to dA methylation (BamHI) and three enzymes (EcoRV, PciI, PvuII) that cleave different base-pair sequences, the activities of which are known to be blocked by adenine methylation. The commercial M.EcoGII and pA-M.EcoGII recombinant protein methyltransferase reactions on the 7-kb linear dsDNA were carried out the same as described above. All restriction endonucleases used in this study were purchased from NEB. For the restriction enzyme digest reaction, each 30 μL reaction volume contained 500 ng methylated dsDNA, the appropriate digestion buffer, time, and amount of enzyme following the manufacturer’s protocol of NEB. The enzymes were inactivated at 80 °C for 20 min. All samples were loaded to 1% agarose gel for analysis.

### In vitro pA-Tn5 transposome preparation

The pA-Tn5 was purchased from Vazyme (Vazyme, S603). To generate the pA-Tn5 adapter transposon, 7 μL of a 50 μM equimolar mixture of pre-annealed Tn5MEDS-A and Tn5MEDS-B oligonucleotides, 40 μL of 7.5 μM pA-Tn5 fusion protein, and 28 μL coupling buffer were mixed. The mixture was incubated for 1 h on a Thermocycler at room temperature and then stored at − 20 °C.

### CUT&TAG for bench-top application

Gently resuspend and withdraw enough of such that there will be 10 μL for each final sample. Place the tube on a magnet stand to clear (30 s to 2 min). Withdraw the liquid, and remove it from the magnet stand. Add 1.5 mL Binding buffer (20 mM HEPES pH 7.5, 10 mM KCl, 1 mM CaCl_2,_ and 1 mM MnCl_2_), mix by inversion or gentle pipetting, remove liquid from the cap and side with a quick pulse on a microcentrifuge. Resuspend in a volume of Binding buffer equal to the volume of bead slurry (10 μL per final sample). Ten microliters of activated beads was added per sample and incubated at room temperature for 15 min. Cells were harvested, counted, and centrifuged for 3 min at 600 × *g* at room temperature. Then, 500,000 cells were washed 2 × in 1.5 mL Wash Buffer (20 mM HEPES pH 7.5, 150 mM NaCl, 0.5 mM Spermidine, 1 × Protease inhibitor cocktail), after that the cells were resuspended in 1.5 mL Wash Buffer by gentle pipetting in a 2-mL tube. The unbound supernatant was removed by placing the tube on the magnet stand to clear and pulling off all of the liquid. The bead-bound cells were resuspended with 50 μL Dig-Wash Buffer (20 mM HEPES pH 7.5, 150 mM NaCl, 0.5 mM Spermidine, 1 × Protease inhibitor cocktail, 0.05% Digitonin) containing 2 mM EDTA and a 1:100 dilution of the appropriate primary antibody. The primary antibody was incubated on a rotating platform overnight at 4 °C. The primary antibody was removed by placing the tube on the magnet stand to clear and pulling off all of the liquid. The secondary antibody was diluted 1:100 in 100 μL of Dig-Wash buffer, and cells were incubated at room temperature for 1 h. Cells were washed using the magnet stand twice for 5 min in 1 mL Dig-Wash buffer to remove unbound antibodies. 0.04 μM of pA-Tn5 was prepared in 150 μL Dig-Med Buffer (0.05% Digitonin, 20 mM HEPES, pH 7.5, 300 mM NaCl, 0.5 mM Spermidine,1 × Protease inhibitor cocktail). After removing the liquid on the magnet stand, 150 μL was added to the cells with gentle vortexing, which was incubated with pA-Tn5 at room temperature for 1 h. Cells were washed twice for 5 min in 1 mL Dig-Med Buffer to remove unbound pA-Tn5 protein. Next, cells were resuspended in 300 μL Tagmentation buffer (10 mM MgCl_2_ in Dig-Med Buffer) and incubated at 37 °C for 1 h. To stop tagmentation, 15 μL EDTA, 3 μL 10% SDS, and 2.5 μL 20 mg/ml proteinase K were added to 300 μL of the sample, which was incubated in a water bath overnight at 55 °C. Three hundred twenty microliters PCI was added to the tube and mixed by full-speed vortexing for 2 s. The upper phase was transferred to a phase-lock tube. Three hundred twenty microliters chloroform was added to the tube and inverted ~ 10 × to mix. The tube was centrifuged for 3 min at room temperature at 16,000 × *g*. The aqueous layer was transferred to a fresh 1.5-mL tube containing 750 μL 100% ethanol and mixed by pipetting. The tube was chilled on ice and centrifuged for at least 10 min at 4 °C 16,000 × *g*. The liquid was removed, and 1 mL 100% ethanol was added to the tube, then centrifuged 1 min at 4 °C 16,000 × *g*. The liquid was carefully poured off and air dry. When the tube is dry, 25 μL 10 mM Tris–HCl pH 8, 0.1 mM EDTA was added to the tube and vortexed on full of dissolving the genomics DNA.

### BIND&MODIFY for bench-top application

In total, 500,000 cells were used in each BIND&MODIFY assay. Cells were harvested, counted, and centrifuged for 3 min at 600 × *g* at room temperature. Cells were first lightly fixed by adding formaldehyde (Thermo Fisher, 28,906) to a final concentration of 0.1% in 1.5 ml PBS, and incubated at room temperature for 15 min. 2.5 M Glycine was added to final concentration of 0.125 M to quench the additional formaldehyde. Fixed cells were then washed twice in 1.5 mL Wash Buffer (20 mM HEPES pH 7.5, 150 mM NaCl, 0.5 mM Spermidine, 1 × Protease inhibitor cocktail) by gentle pipetting. Ten microliters of activated beads was added per sample and incubated at room temperature for 15 min. The unbound supernatant was removed, and bead-bound cells were resuspended in 100 μL Dig-Wash Buffer (20 mM HEPES pH 7.5, 150 mM NaCl, 0.5 mM Spermidine, 1 × Protease inhibitor cocktail, 0.02% Digitonin, 0.1% BSA) containing 2 mM EDTA and a 1:100 dilution of the appropriate primary antibody. Primary antibody incubation was performed on a rotating platform overnight at 4 °C. The primary antibody was removed by placing the tube on the magnet stand to clear and pulling off all of the liquid. No secondary antibody was used for BIND&MODIFY. Cells were washed three times using the magnet stand for 5 min in 1 mL Dig-Wash buffer. Twenty microliters of pA-M.EcoGII was prepared in 150 μL Dig-Wash Buffer. After removing the liquid on the magnet stand, 150 μL of pA-M.EcoGII containing Dig-Wash buffer was added to the cells with gentle vortexing, which was incubated at room temperature for 1 h. Cells were washed 3 × for 5 min in 1 mL Dig-Wash Buffer and 1 × for 5 min in 1 mL Low salt buffer (20 mM HEPES pH 7.5, 0.5 mM Spermidine, 1 × Protease inhibitor cocktail, 0.02% Digitonin) to remove unbound pA-M.EcoGII protein. Next, cells were resuspended in 300 μL Methylation buffer (1 × CutSmart Buffer pH 7.9, 1 × Protease inhibitor cocktail, 0.05% Digitonin, 0.1% BSA). The reaction was activated by adding 7.5 μL SAM of 32 mM at 37 °C in a thermomixer. Additional 5 μL of 32 mM SAM was added to the tube at 7.5 and 15 min. The reaction was stopped at 30 min by placing the tube on the magnet stand to clear and pulling off all of the liquid. The bead-bound cells was resuspended with 300 μL PCI Lysis Buffer (20 mM HEPES pH 7.5, 300 mM NaCl, 0.5 mM Spermidine, 1 × Protease inhibitor cocktail, 0.05% Digitonin, 16.7 mM EDTA, 0.1% SDS, 0.167 mg/mL proteinase K) or Serapure Beads Digestion Buffer (1% Polyvinylpyrrolidone 40, 1% sodium metabisulfite, 0.5 M sodium chloride, 100 mM TRIS, pH 8.0, 50 mM EDTA, 1.25% SDS, 0.167 mg/mL proteinase K), and incubated in water bath overnight at 55 °C. The method for genomic DNA extraction was by PCI or Serapure Beads.

We refer this BIND&MODIFY Protocol as Protocol v1 and can be accessed at Protocols.io with the following doi: (dx.doi.org/10.17504/protocols.io.b2ahqab6).

To increase the methylation efficiency, the following changes were applied and used for targeting CTCF for experiments. The major changes were as follows: (1) introduce Protein A block step before antibody binding; (2) reduce primary antibody binding time from overnight to 2 h at 4°; (3) add 0.5 mM spermidine in the methylation buffer; (4) increase methylation reaction time from 30 to 120 min, and replenish 32 mM SAM at 30, 60, and 90 min; (5) add 1 ng/µl dsDNA 1 ng/µl in the activation buffer (optional).

We refer this BIND&MODIFY protocol as Protocol v2 and can be accessed at Protocol.io with the following doi: (dx.doi.org/10.17504/protocols.io.3byl4j7m8lo5/v1)

### In vitro validation of BIND&MODIFY

To define the m^6^A methylation base calling probability cut-off of BIND&MODIFY, we used the IgG and no antibody treatment control on MCF-7 cell line followed by BIND&MODIFY reaction. The DNA from the reactions were purified, and library were prepared following the manufacturer’s protocol of SQK-LSK109 (Nanopore, SQK-LSK109). The library was sequenced in the ONT PromethION platform with R9.4.1 flow cell.

To define the m^6^A methylation base calling probability cut-off 0.53’s precision and specificity, lambda genome DNA was PCR amplified by 6 pairs of primers:

- Primer1
  - FW 5′-ACCTCGCGGGTTTTCGCTATTTATG-3′
  - RV 5′-ATATCGCGGATGAAGCAACGC-3′
- Primer2
  - FW 5′-GCGTTGCTTCATCCGCGATAT-3′
  - RV 5′-GATTTCAGTGTATGACGACCAGAGCG-3′
- Primer3
  - FW 5′-CGCTCTGGTCGTCATACACTGAAATC-3′
  - RV 5′-CCCGCACTATCGGAAGTTCAC-3′
- Primer4
  - FW 5′-GTGAACTTCCGATAGTGCGGG-3′
  - RV 5′-GGATAAAACATGGGATGACGAC-3′
- Primer5
  - FW 5′-GTCGTCATCCCATGTTTTATCCAG-3′
  - RV 5′-CATGACAGGAAGTTGTTTTACTGG-3′
- Primer6
  - FW 5′-CCAGTAAAACAACTTCCTGTCATG-3′
  - RV 5′-CGTAACCTGTCGGATCACCGGAAA-3′

The PCR products from 6 reactions were purified and pooled at equal molar ratio. The pooled lambda DNA were used as template followed by four conditions, (1) non treated; (2) Dam treated; (3) M.EcoGII treated; (4) pA-M.EcoGII treated. The input lambda DNA was 1.0 μg, and the reactions were setup as follows: non treated (10xCutSmart buffer 5 μl, 32 mM SAM 10 μl, H_2_O up to 50 μl, 37 °C for 2 h), Dam treated (dam methyltransferase reaction buffer 5 μl, dam methyltransferase 8 μl, 32 mM SAM 10 μl, H_2_O up to 50 μl, 37 °C for 2 h), M.EcoGII treated (M.EcoGII methyltransferase 8 μl, 10xCutSmart buffer 5 μl, 32 mM SAM 10 μl, H_2_O up to 50 μl, 37 °C for 2 h), and pA.M.EcoGII treated (pA-M.EcoGII 8 μl, 10xCutSmart buffer 5 μl, 32 mM SAM 10 μl, H_2_O up to 50 μl, 37 °C for 2 h). The M.EcoGII and pA-M.EcoGII-treated reactions were inactivated at 65 °C for 10 min, and the Dam-treated reaction was inactivated at 65 °C for 20 min. The DNA from the reactions were then purified by 0.5 × Ampure XP beads, and the libraries were prepared following the manufacturer’s protocol of SQK-LSK109 (Nanopore, SQK-LSK109). The library was sequenced in the ONT PromethION platform with R9.4.1 flow cell.

To test the resolution of BIND&MODIFY, lambda genome DNA was PCR amplified by a pair of primers (FW 5′-TCTTCCGATCTAAGCAGTGGTATCAACGCAGAGTACCATGCAATTACAACAT/i5MedC/AGGGTAAC-3′;RV 5′-CCCTGTATTGCTGAAATGTGATTTC-3′), and the forward primer carried one 5mC at fixed position. The 700-bp PCR product was purified and undergoes BIND&MODIFY reaction with 5mC primary antibody. The DNA from the reactions were purified by 0.8 × Ampure XP beads, and library was prepared following the manufacturer’s protocol of SQK-LSK109 (Nanopore, SQK-LSK109). The library was sequenced in the ONT PromethION platform with R9.4.1 flow cell.

### SDS treatment to increase pA-M.EcoGII labeling efficiency

Upon cell harvest, 1 × 10^6^ cells were fixed with 1% formaldehyde, after quenching with 0.125 M glycine, the cells were treated with 0.1% SDS for 65° for 5 min in Dig-wash buffer. SDS was quenched by 1% final concentration Triton X-100. After centrifuging at 2500 rpm, the cells were treated with pA-M.EcoGII for 3 h in methylation buffer (7.5 µl 32 mM SAM, 6 µl 50X proteinase inhibitor, 1.5 µl 20% BSA, 50 µl pA-M.EcoGII, NF water up to 300µ), and replenish 7.5 µl 32 mM SAM and 10 µl pA-M.EcoGII every 1 h. Afterwards, the cells were lysed and total DNA were extracted by Seramega beads. Two hundred nanograms of extracted 0.1% SDS(+ / −) pA-M.EcoGII modified DNA was treated DpnI (+ / −) digestion, and 1% agarose gel electrophoresis was performed.

### CUT&TAG library preparation

To amplify libraries, 21 μL of CUT&TAG genomic DNA was mixed with 2 μL of a universal i5 and a uniquely barcoded i7 primer, using a different barcode for each sample. A volume of 25 μL KAPA HIFI ready mix (KAPA, KK2602) was added and mixed. The sample was placed in a thermocycler with a heated lid using the following cycling conditions: 72 °C for 5 min; 98 °C for 30 s; 14 cycles of 98 °C for 10 s, 63 °C for 30 s and 72 °C for 15 s; final extension at 72 °C for 1 min and hold at 8 °C. Post-PCR clean-up was performed by adding 1.3 × volume of Ampure XP beads (Beckman Counter), and libraries were incubated with beads for 5 min at RT, washed twice gently in 80% ethanol, and eluted in 30 μL 10 mM Tris pH 8.0. the library was analyzed using Agilent 2100 (2100 Bioanalyzer Instrument, G2939BA). Then the library was sequenced in MGI2000 platform with PE100 + 100 + 10 sequencing.

### BIND&MODIFY library preparation

The BIND&MODIFY library was prepared following the manufacturer’s protocol of SQK-LSK109 (Nanopore, SQK-LSK109). The library was sequenced in the ONT PromethION platform with R9.4.1 flow cell.

### Basecalling and DNA methylation calling

Reads from the ONT data were performed using megalodon (V2.2.9), which used Guppy basecaller to basecalling and Guppy model config res_dna_r941_min_modbases-all-context_v001.cfg has been released into the Rerio repository which was used to identify DNA m^6^A methylation. megalodon_extras was used to get per read modified_bases from the megalodon basecalls and mappings results. In order to further explore the accurate threshold of methylation probability, a control sample with almost no m^6^A methylation was used as background noise, and Gaussian mixture model was used to fit the methylation probability distribution generated by megalodon.

### ChIP-seq data processing

Demultipexed fastq files were mapped to the hg19 genome using Bowtie2 (2.4.1) [53] with the following settings: bowtie2 –end-to-end –very-sensitive –no-mixed –no-discordant –phred33 -I 10 -X 700. peaks were called using MACS2 (v.2.1.0) [54] with the following settings: -g 12,000,000-f BAMPE.

### CUT&TAG data processing

Demultipexed FASTQ files were mapped to the HG19 genome using Bowtie2 with the following settings: bowtie2 –end-to-end –very-sensitive –no-mixed –no-discordant –phred33 -I 10 -X 700. Because a constant amount of pA-Tn5 was added to CUT&TAG reactions and brings along a fixed amount of *E. coli* DNA, we used bowtie2 with the parameters mentioned above to remove *E. coli* DNA and conduct normalization according to the CUT&TAG tutorial [17]. CUT&TAG peaks were called using SEACR(1.3) [55] with default parameters.

### RNA-seq analysis

RNA-seq expected counts of the MCF-7 and HeLa cell lines in all replicates were corrected to be TPM, the mean TPM of all replicates was used as the expression level of each gene for subsequent analysis.

### Histone modification score

HG19 genome and the gene elements were processed into 50-bp bins sliding 5 bp by Bedtools (v2.27.1) [56]. The histone modification score over multi-base-pair windows was calculated as methylation ratio = m^6^A bases in all covered reads under bin/ adenosine bases in all covered reads under bin. And the histone modifications score of each single molecule in the bin was also calculated.

### Single-molecule DNA heterogeneity clustering

The histone modification score of a single molecule in each gene was calculated, and the value in each gene was sorted from small to large by the gene unit. Then the hierarchical clustering was performed for the diversity of single-molecule accessibility of each gene. KOBAS3.0 [57] was used for KEGG and GO analysis for each cluster.

### Assess replicate reproducibility

To study the reproducibility between replicates, the genome was split into 50-bp bins and sliding 5 bp, then a Pearson correlation of the log2-transformed values of m^6^A methylation ratio in each bin was calculated between replicate datasets.

### SV calling

We used NGMLR (v0.2.7) [58] to compare the read of ONT to the human reference genome of hg19 to get the BAM file for comparison. Then we used samtools (v1.2) to sort the bam files. The sniffles (v1.0.12) [58] with the parameter –genotype -T 8 -S 8 were then used to call the structural variation on the bam file created in the previous step.

### Long-distance co-labeling coefficient between cis-regulators and promoters

#### Regional co-labeling coefficient assessment

To evaluate co-labeling coefficient (CC) patterns along the genome, we applied CC as follows. Each chromosome in the genome was split into windows of size *w*. For each such window (*i*, *i* + *w*), we identified another window (*j*,*j* + *w*) such that the span (*i*,*j*,*w*) was covered by ≥ *N*(*N* = 1) reads. For each single spanning molecule *k*, co-labeling coefficient scores (A) in each bin were then aggregated and binarized as described above. The local co-labeling coefficient matrix between two windows was calculated as (Eq. 1), the aggregate co-labeling coefficient matrix was calculated as (Eq. 2), the aggregate co-labeling coefficient matrix was calculated as (Eq. 2), the distance dependent co-labeling coefficient matrix was calculated as (Eq. 3):

1 $$CCi,j,w=0.5-\frac{\left|Ai,w-Aj,w\right|}{Ai,w+Aj,w}$$

2 $$CCi,j,w={\sum }_{i=1}^{n/w}\;{\sum }_{j=1}^{n/w}\left(0.5-\frac{\left|Ai,w-Aj,w\right|}{Ai,w+Aj,w}\right)$$

3 $$CCi,j,w= {\sum }_{i=1}^{n/w}\;{\sum }_{j=1}^{n/w}\left(0.5-\frac{\left|Ai,w-Aj,w\right|}{Ai,w + Aj,w}\right)*\left(\frac{\left|LAi- LAj\right|}{n}+1\right)$$

where *n* is the length of selected region, *L* is the location of region.

#### Long-distance co-labeling coefficient correlation between cis-regulators and promoters

To calculate long-distance co-labeling coefficient correlation between cis-regulators and promoters, we selected region containing cis-regulators in 2000 bp upstream of gene promoter. The calculation method of co-labeling coefficient correlation score is similar with co-labeling coefficient Assessment (B). After aggregating the co-labeling coefficient score of cis-regulators region, the upper quartile of variance of co-labeling coefficient score in per gene were selected and expression matrix of all gene was clustered by hierarchical cluster. Clusters with high co-labeling coefficient scores were selected for downstream analysis.

### Data simulation

We used ART (V2.5.8) to generate 4 Illumina HiSeq 2500 short-read sets from centromeric sequences and repeat region sequences: single end 50 bp, pair end 50 bp, 100 bp, and 150 bp read length, the average insertion size of pair end data is 100, 200, and 300 bp respectively.

### Multiple alignment

Centromere region was selected from cytoBand.txt, which was downloaded from UCSC, and bedtools (v2.27.1) [56] was used to extract centromeres sequence. Following this, ONT data was aligned to the centromere sequence using minimap2(2.22-r1101) [59], then sam files were converted to PAF Records by minimap pafTools. js and reads with coverage greater than 60% were used for downstream analysis. For second-generation sequencing data, alignment was performed by the bowtie2 with default parameter.

### Phasing

We aligned the ONT original data sequence to the hg19 genome by using minimap2 (2.22-r1101) [59]. After obtaining bam files, Medaka was used for calling variation of each chromosome, and then whatshap [60] was used for phasing reads.

### Motif analysis

MEME software (5.4.1) [61] was utilized to perform motif discovery tasks with default parameters. And motif sequence was submitted to GOMo (5.4.1) [62] to identify possible roles (Gene Ontology terms) for motifs.

### ROC evaluation methods

We used a Python package, sklearn, to calculate the ROC curves and AUC, and the statistical terms, sensitivity, and precision are defined in the following equations:

$$\mathrm{Sensitivity}= \frac{\mathrm{TP}}{\mathrm{TP}+\mathrm{FN}}$$

$$\mathrm{Precision }= \frac{\mathrm{TP}}{\mathrm{TP}+\mathrm{FP}}$$

where TP, TN, FP, and FP stand for true positive, true negative, false positive, and false negative, respectively.

### Data normalization

Each chromosome in the genome was split into windows of size *w*. For each such window (*i, i* + *w*), The formula for calculating the normalization Nmi is as follows:

$$\mathrm{Nmi}= \frac{Ci,w}{\sum_{i=1}^{n/w}Ci,w}*10^\wedge$$

For BIND&MODIFY data, *Ci,w* is m6A methylation count in each window. For ChIP-seq data, *Ci,w* is reads count number in each window.

### Comparing of CTCF targeted BIND&MODIFY Hi-C proximity region with background

The Hi-C read pair interaction sites were converted into two upstream and downstream 2.5-kb regions, these read pair regions were intersected with CTCF Peak using BedTools respectively. For the Hi-C read pairs, in which the one end with CTCF peak intersection were selected, their Hi-C interaction read genome sites without CTCF peak intersection were defined as CTCF Hi-C proximate region. We recorded the above CTCF Hi-C proximate region genome sites, applied them to the CTCF targeted BIND&MODIFY data, and calculated the mean methylation ratio + / − 2.5 kb of these geome sites accordingly. In addition, 5000-bp regions of BIND&MODIFY data with no Hi-C interaction and no CTCF peak signal were randomly sampled, and the consistency of the distribution of the two datasets was determined by the Wilcoxon test.

### Peak overlap binomial test

The significance of each histone modification/CTCF region was determined by performing a binomial test of the raw read count of BIND&MODIFY, with overall igG methylation ratio as the null probability. The probabilities were corrected for multiple testing using Benjamini–Hochberg correction, and histone modification/CTCF regions with adjusted *p*-values less than 0.01 and widths greater than 50 bps were determined to be histone modification/CTCF peaks. The intersection of Histone Modification Peaks and ChIP-seq Peak region was shown using Venn diagram. For those ChIP-seq peaks that have no overlap with histone modification region, the Wilcoxon test was used to detect the consistency of distributions of data with overlap and without overlap with histone modification region in the ChIP-seq data.

## Supplementary Information

**Additional file 1. **Supplementary Figures S1-S20.

**Additional file 2.** Data Table for all data generated from this study and external datasets  used.

**Additional file 3.** Review history.

## Data Availability

The datasets generated during and/or analyzed during the current study are available in the Sequence Read Archive (SRA) repository, under BioProject accession PRJNA924005 [63]. These data were used for all Figs. 1, 2, 3, 4, and 5 and  Fig S1-S20. The MCF-7 H3K27me3 ChIP-seq data were obtained from ENCODE Project Consortium [64] under accession ENCSR761DLU [65]. The MCF-7 CTCF ChIP-seq data were obtained from ENCODE Project Consortium [64] under accession ENCSR000DMR [66]. The MCF-7 RNA-seq data were obtained from the Gene Expression Omnibus (GEO) under accession GSE71862 [67] from Barutcu et al. [68]. The MCF-7 Hi-C data were obtained from the Gene Expression Omnibus (GEO) under accession GSE66733 [69]. The MCF-7 WGBS data were obtained from the Gene Expression Omnibus (GEO) under accession GSM3526804 [70]. The HeLa CTCF ChIP-seq data were obtained from ENCODE Project Consortium [64] under accession ENCSR000DUB [71]. The HeLa H3K4me3 ChIP-seq data were obtained from the Gene Expression Omnibus (GEO) under accession GSM733682 [72]. The HeLa RNA-seq data were obtained from the Gene Expression Omnibus (GEO) under accession GSE86661 [73]. The HeLa Hi-C data was obtained from 4D Nucleosome Project [74] under accession 4DNFIKKZD99T [75]. The detailed external datasets used in this study can be accessed in Additional file 1: Table S1. HG19 genome, short interspersed nuclear elements (SINE), and long interspersed nuclear elements (LINE) region were downloaded from NCBI. TES, TTS, and other gene elements were downloaded from the UCSC Table Browser. MCF-7 CTCF binding site was downloaded from the CTCFBSDB_v2.0 [76]. Promoter and cis-regulator region was annotated by 10X Genomics ATAC database. All custom code for BIND&MODIFY data analysis is available from the following GitHub repository [77] (https://github.com/genometube/Bind_Modify) and is released under the terms of the GNU General Public License (GPL) version 3.0 (https://opensource.org/license/gpl-3-0/). A copy of the GPL license is included in the repository and can be found at the following link: https://github.com/genometube/Bind_Modify/blob/main/LICENSE. The source code for BIND&MODIFY data analysis is also available in Zenodo [78] (10.5281/zenodo.7538704). pA-M.EcoGII expression plasmid is available from Addgene (pTXB1-pA-M.EcoGII; Addgene,192873).
