# Supplementary material for: BIND&MODIFY: a long-range method for single-molecule mapping of chromatin modifications in eukaryotes
Source: Genome Biol. 2023 Mar 29;24:61. doi: 10.1186/s13059-023-02896-y (PMC10052867; doi:10.1186/s13059-023-02896-y)
Supplement: Supplementary file 1 — Additional file 1. Supplementary Figures S1-S20. [file 13059_2023_2896_MOESM1_ESM.docx]

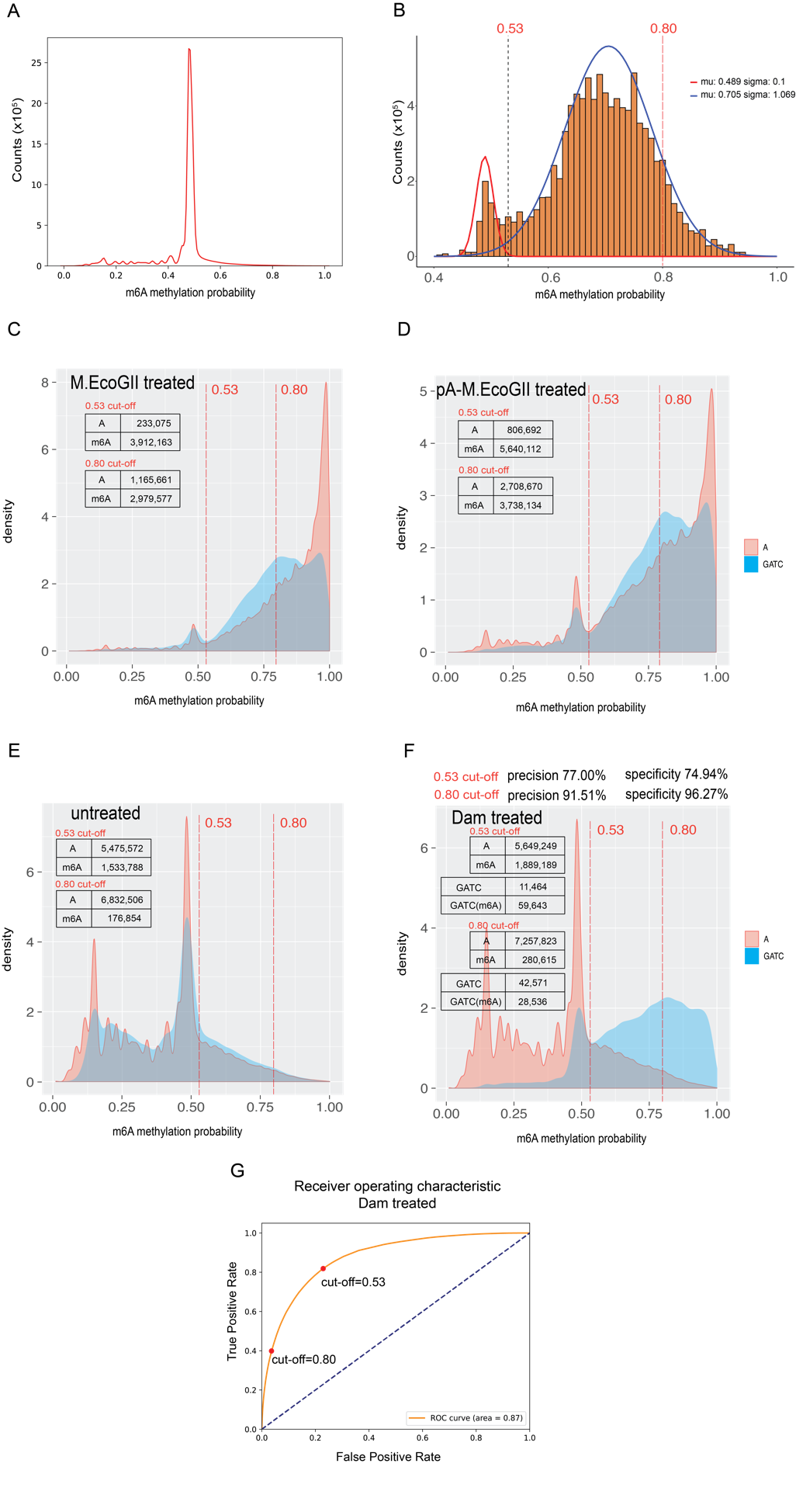


**Fig S1. The data training of the m6A calling in the BIND&MODIFY.**

(A) The density map of the m^6^A calling probability in the negative control (unmodified DNA). The unmodified MCF-7 genomic DNA set as negative control and used as the background m^6^A noise. The x-axis was the m^6^A calling probability in algorithm. (B) The density map of the m^6^A calling probability in pA-M.EcoGII treated MCF-7 genomic DNA (mostly methylated). By the mathematics modeling, we could find two peaks. The sharp peak represented the non-modified sites, and the wide peak represented the truly modified sites. The m^6^A probability 0.53 was chosen as the cut-off value. (C) The density map of the m6A calling probability in commercial M.EcoGII treated lambda DNA. The true positive rate of commercial M.EcoGII treated lambda DNA, TP_M.EcoGII_ =m^6^A/(A+m^6^A)=0.94. Our algorithm’s m^6^A base calling accuracy is similar to the M.EcoGII labeling efficiency on lambda DNA reported in literature. (D) The density map of the m^6^A calling probability in pA-M.EcoGII treated lambda DNA. The true positive rate of our pA-M.EcoGII treated lambda DNA, TP_pA_-_M.EcoGII_ =m^6^A/(A+m^6^A)=0.87. Our engineered pA-M.EcoGII showed high labeling efficiency, though slightly lower than commercial M.EcoGII. (E) The density map of the m^6^A base calling probability in untreated lambda DNA. True negative rate of untreated lambda DNA was calculated TN_untreated_=A/(A+m^6^A)=0.78, and false positive rate, FP_untreated_ was 0.22. (F) The density map of the m^6^A calling probability in Dam treated lambda DNA. As Dam methyltransferase specifically modify adenine in the GATC motif at high efficiency, GATC motif was used to calculate the precision and specificity of our m^6^A probability base calling algorithm. When m^6^A probability cut-off was set as 0.53, TN_Dam_ was 0.75, FP_Dam_ was 0.25. The true positive rate, TP_dam_, was Gm^6^ATC/(GATC+Gm^6^ATC)=0.84. The false negative rate, FN_dam_, was 0.26. Precision=TP_dam_/(TP_dam_+FP_dam_)=0.77. Specificity=TN_dam_/(TN_dam_+FP_dam_)=0.75. For precise quantification of high confident m^6^A base calling, we also set m^6^A probability cut-off value to 0.80, TN_Dam_ was 0.96, FP_Dam_ was 0.04. The true positive rate, TP_dam_, was Gm^6^ATC/(GATC+Gm^6^ATC)=0.40. The false negative rate, FN_dam_, was 0.60. Precision=TP_dam_/(TP_dam_+FP_dam_)=0.92. Specificity=TN_dam_/(TN_dam_+FP_dam_)=0.96. For (C)-(F), red dashed line showed m^6^A probability cut-off 0.53 and 0.80. (G) Receiver-Operation-Curve (ROC) for different m^6^A probability cut-off value in Dam treated lambda DNA.


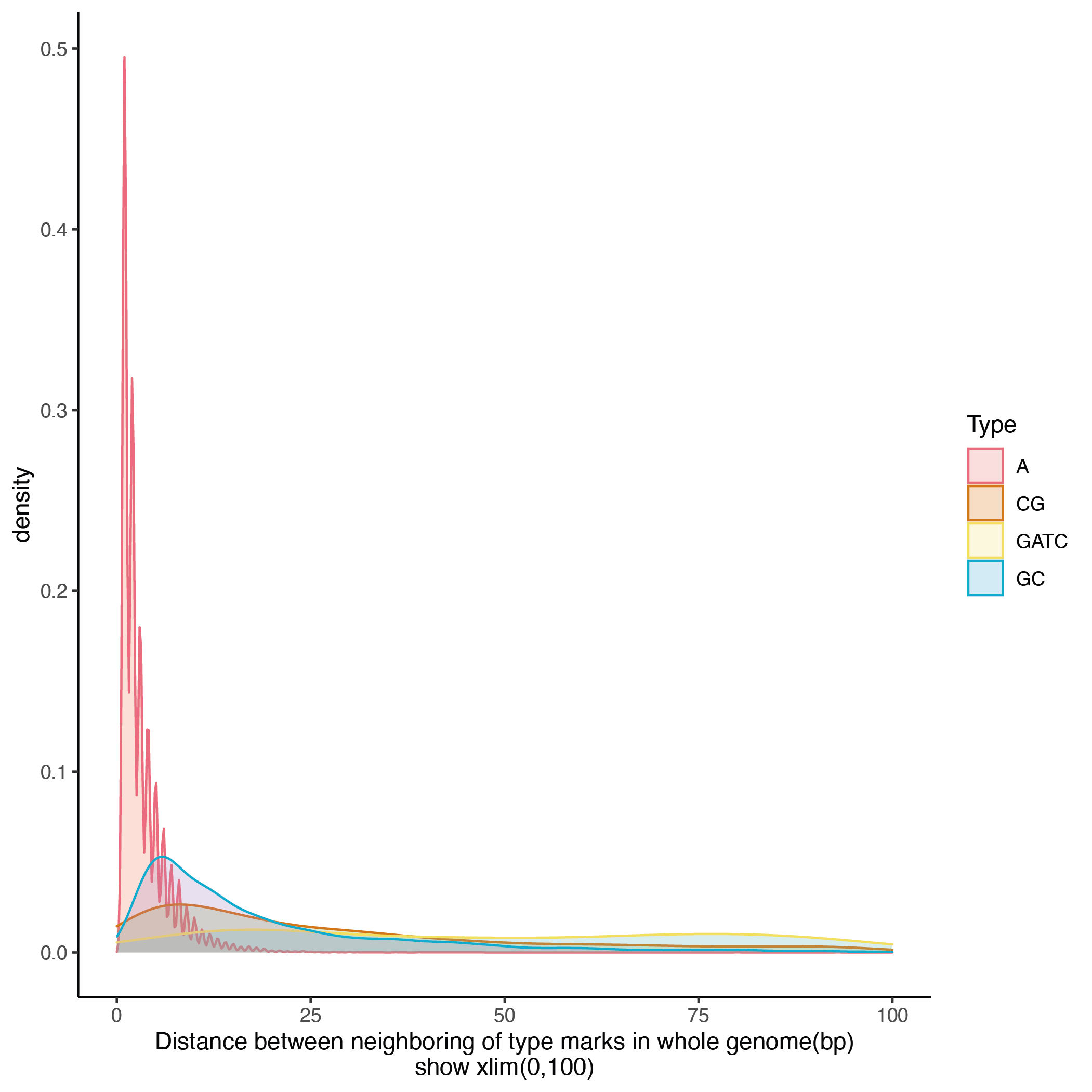


**Fig S2. Distribution of adenine motif in the genome.** The y-axis indicated the distance between the neighboring motif and the x-axis indicated the density. The shorter distance suggested the higher density of motif in the genome.


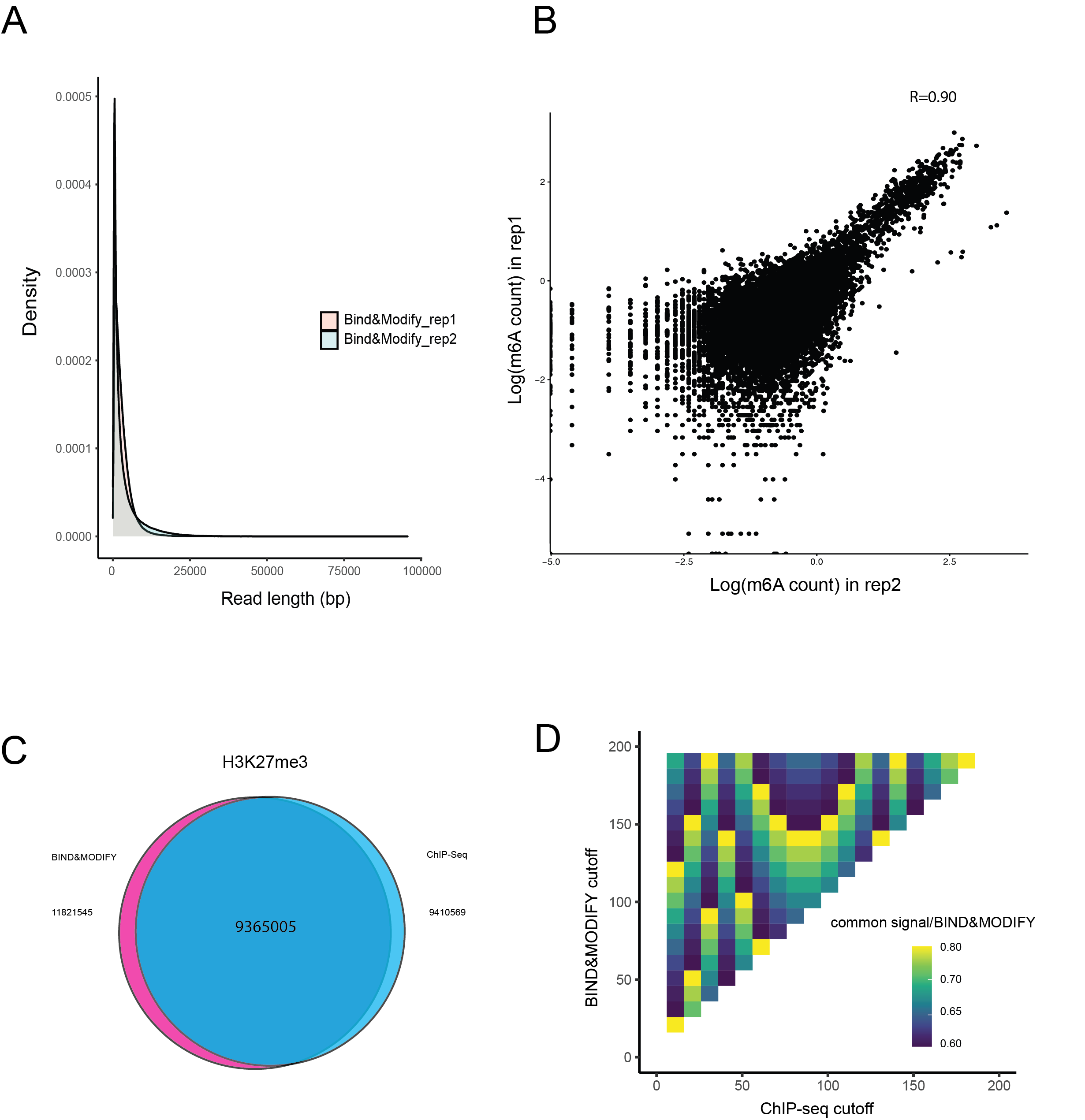


**Fig S3. General sequencing parameter of BIND&MODIFY experiments and overlap between BIND&MODIFY and ChIP-seq**

(A) The read length distribution of the nanopore sequencing in BIND&MODIFY experiments. (B) The reproducibility of the BIND&MODIFY method. The x-axis and y-axis represented the log m6A counts of genome 50bp bins in two replicates. The two replicates (rep1 and rep2) were two technological replicates, which was done in different experimental trials (including cell culture, antibody binding, pA-M.EcoGII binding, sequencing). (C) Venn diagram of signals overlap (50bp windows) in ChIP-seq and BIND&MODIFY. The genome was segmentate to 50bp bins. The signal region in BIND&MODIFY was identified by m6A counts>1. The signal region in ChIP-seq was identified by reads number>0. (D) Plot of the various cut-off values. We selected the different cut-off combinations between BIND&MODIFY and ChIP-seq (refer to C). The color indicated the percentage of the signal regions could be detected in ChIP-seq under corresponding cut-off sets.

**
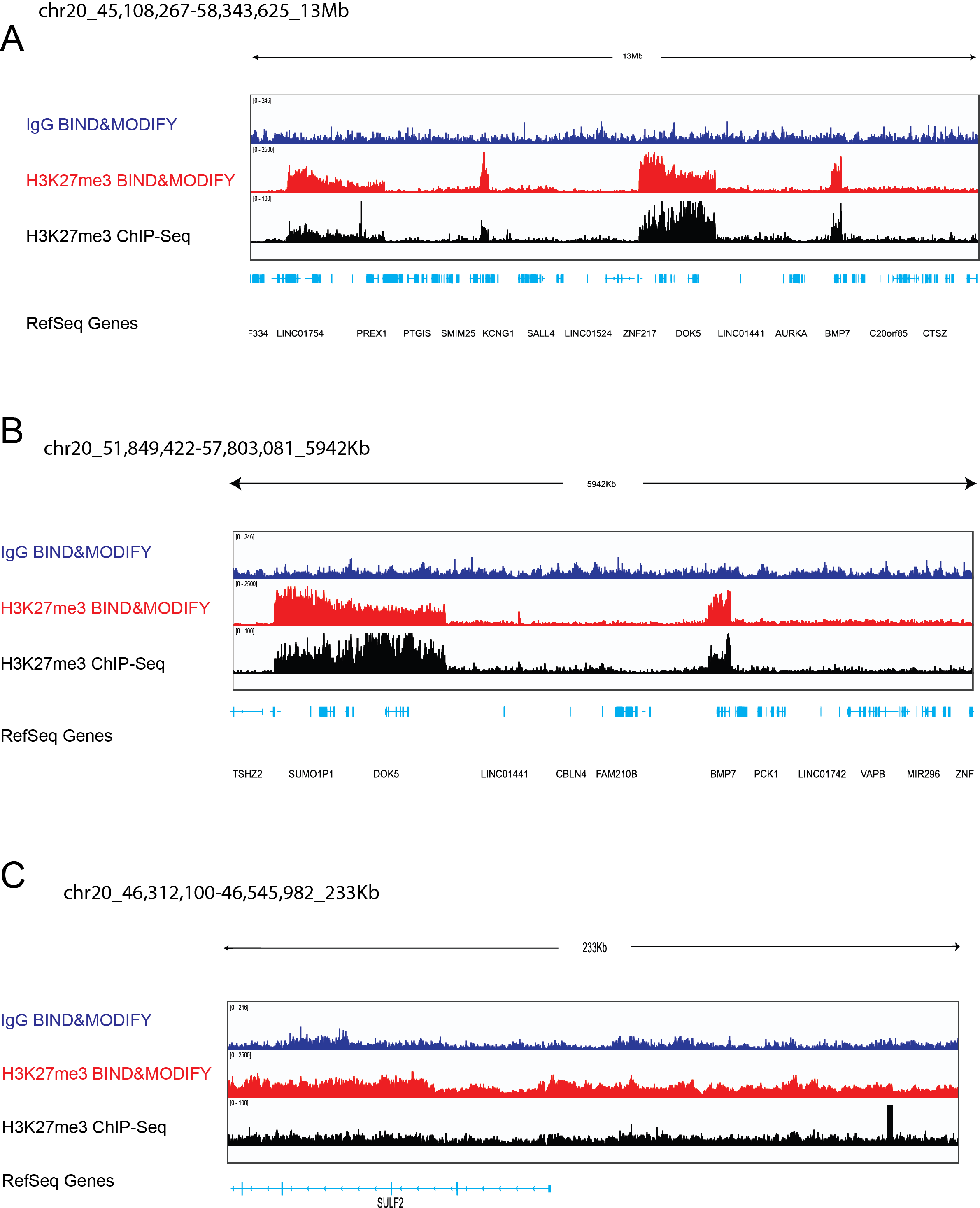
**

**Fig S4. The H3K27me3 signal, by BIND&MODIFY and ChIP-seq, in genome scale view.**


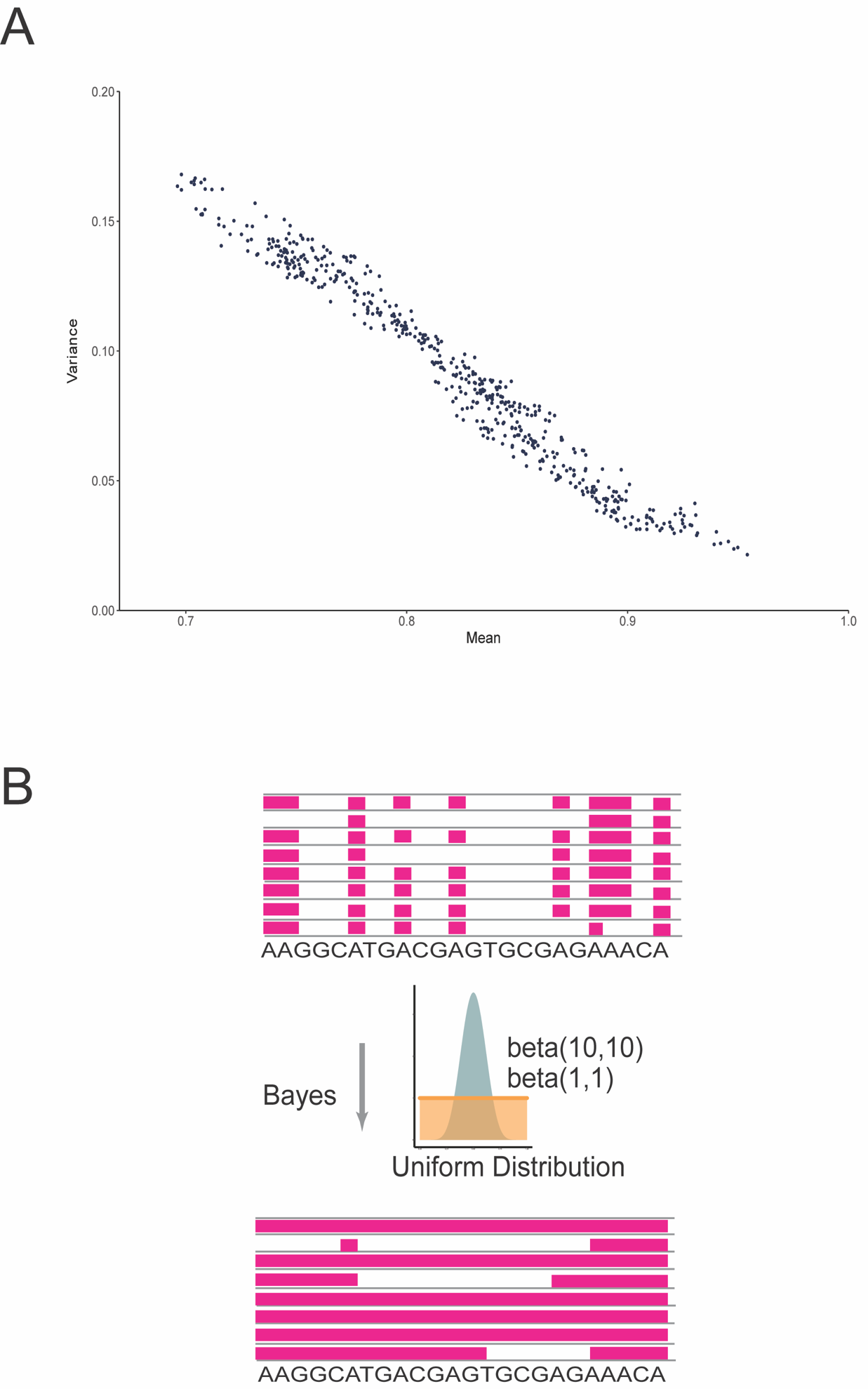


**Fig S5. The m6A base calling algorithm calculations.** The correlation between methylation means and variance. In the high methylation region (possibly pA-EcoGII bound region), the variance is small.


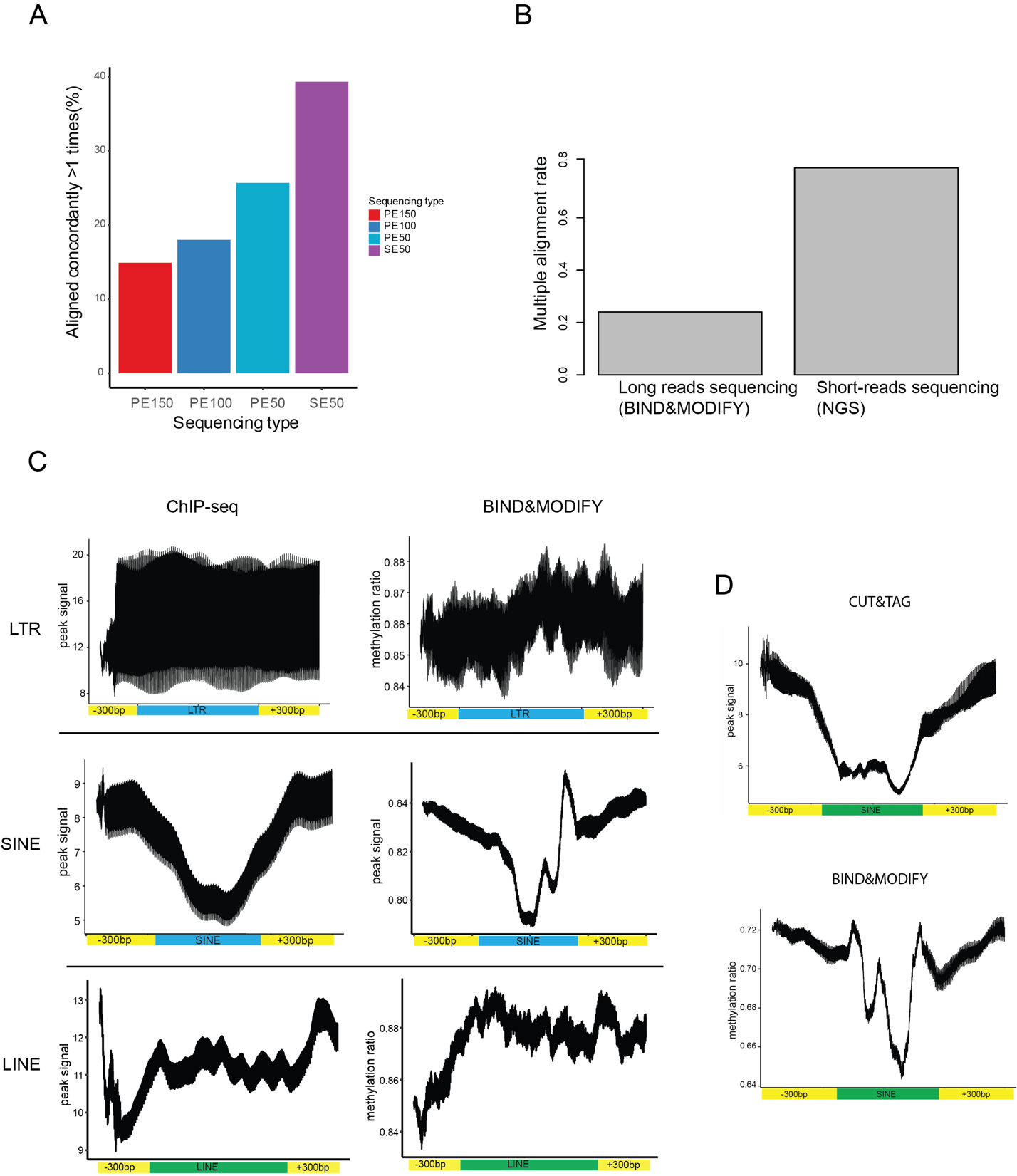


**Fig S6. The BIND&MODIFY improved the signal on the retrotransposon regions.** (A) The simulation of the NGS alignment in retrotransposon regions. We simulated the reads with different length based on the retrotransposon regions (PE50-paired end sequencing 50bp, SE50-single end sequencing 50bp) (Method). The multiple alignment rates decreased with the increasing read length. (B) In the real sequencing data, the SE50 demonstrated higher multiple alignment rates than the simulated SE50, due to the randomness size distribution. The BIND&MODIFY have the lower multiple alignment rate, which may offer the more accurate epigenomic location. (C) The H3K27me3 signal comparison between ChIP-seq and BIND&MODIFY on LTR, SINE, LINE. The LTRs with size 350~450bp were selected and centered. The moving average H3K27me3 signals on the upstream/downstream 300bp of these LTRs, including LTRs, were plotted with corresponding genomic sites. The y-axis (ChIP-seq) indicated the normalized read counts of ChIP-seq. The y-axis (BIND&MODIFY) indicated the normalized methylation ratio of m6A in BIND&MODIFY with nanopore sequencing. The SINEs with size 250~350bp and the LINE with size 900-1100bp were selected and centered. The moving average H3K27me3 signals on the upstream/downstream 300bp of these SINE/LINEs, including SINE/LINEs, were plotted with corresponding genomic sites. (D) The BIND&MODIFY/ CUT&TAG showed CTCF pattern in SINEs. The SINEs with size 350~450bp were selected and centered. The moving average CTCF signals on the upstream/downstream 300bp of these SINEs, including SINEs, were plotted with corresponding genomic sites. They y-axis (CUT&TAG) indicated the normalized read counts of CUT&TAG. The y-axis (BIND&MODIFY) indicated the normalized the methylation ratio of m6A in BIND&MODIFY with nanopore sequencing.


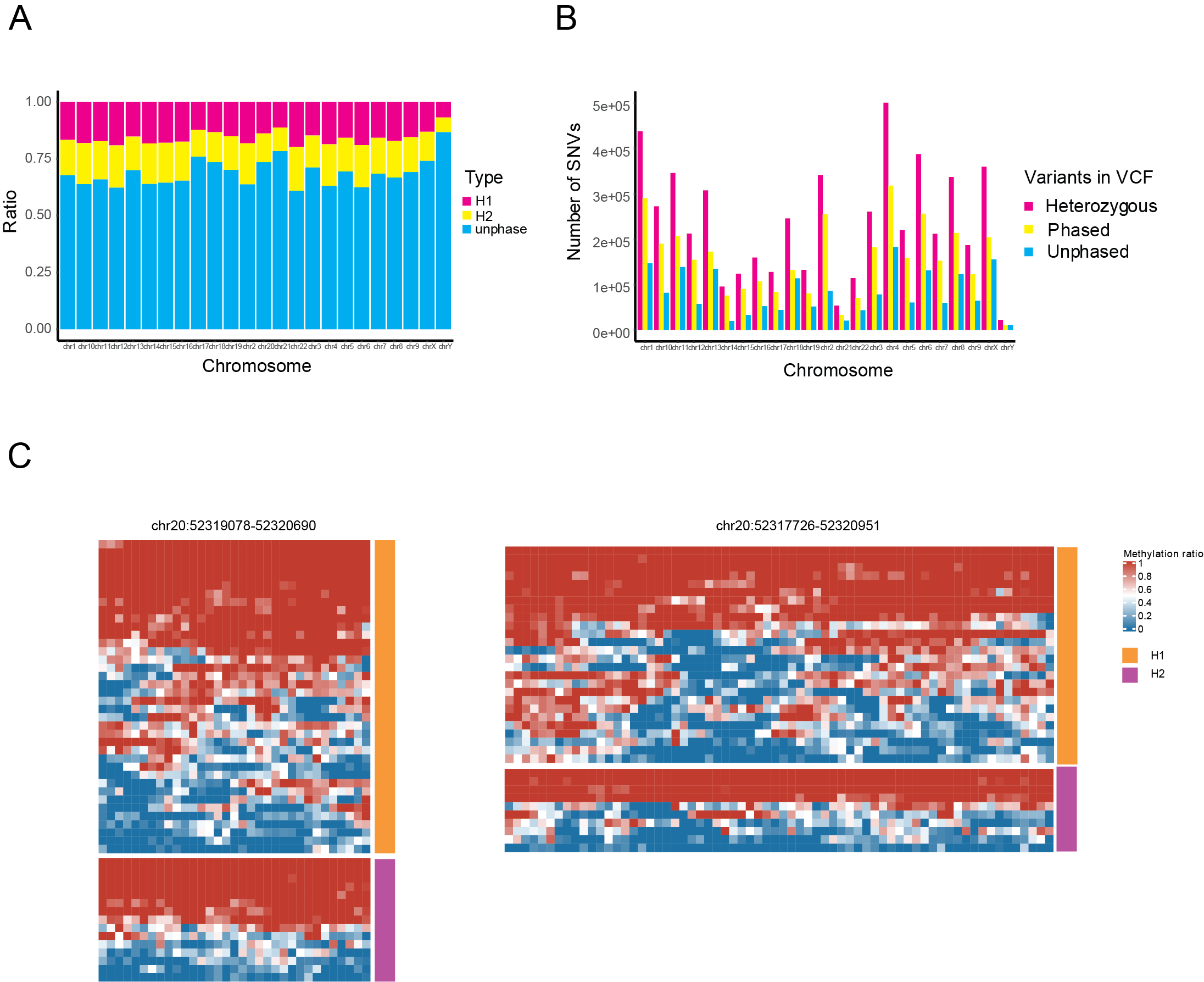


**Fig S7. The long-range sequencing phases the genome to paternal and maternal chromosomes.** (A) We showed the portion of the reads could be phased on each chromosome (method). Around 30% reads could be phased to paternal/maternal (H1/H2) genome. (B) 70% of the SNVs (single-nucleotide variants) found genome could be phased. Red bar means the total number of the single-nucleotide variants found on genome as heterozygous by using nanopore sequencing. The y-axis indicated the sequenced SNV number on each chromosome. The heterozygous SNVs could be phased by meeting the criteria of software. (C) We shared two genomic segments with H3K27me3 signal as examples (Chr20:52319078-52320690, Chr20:52317726-52320951). The H3K27me3 demonstrate the different distribution pattern between H1/H2 chromosomes.


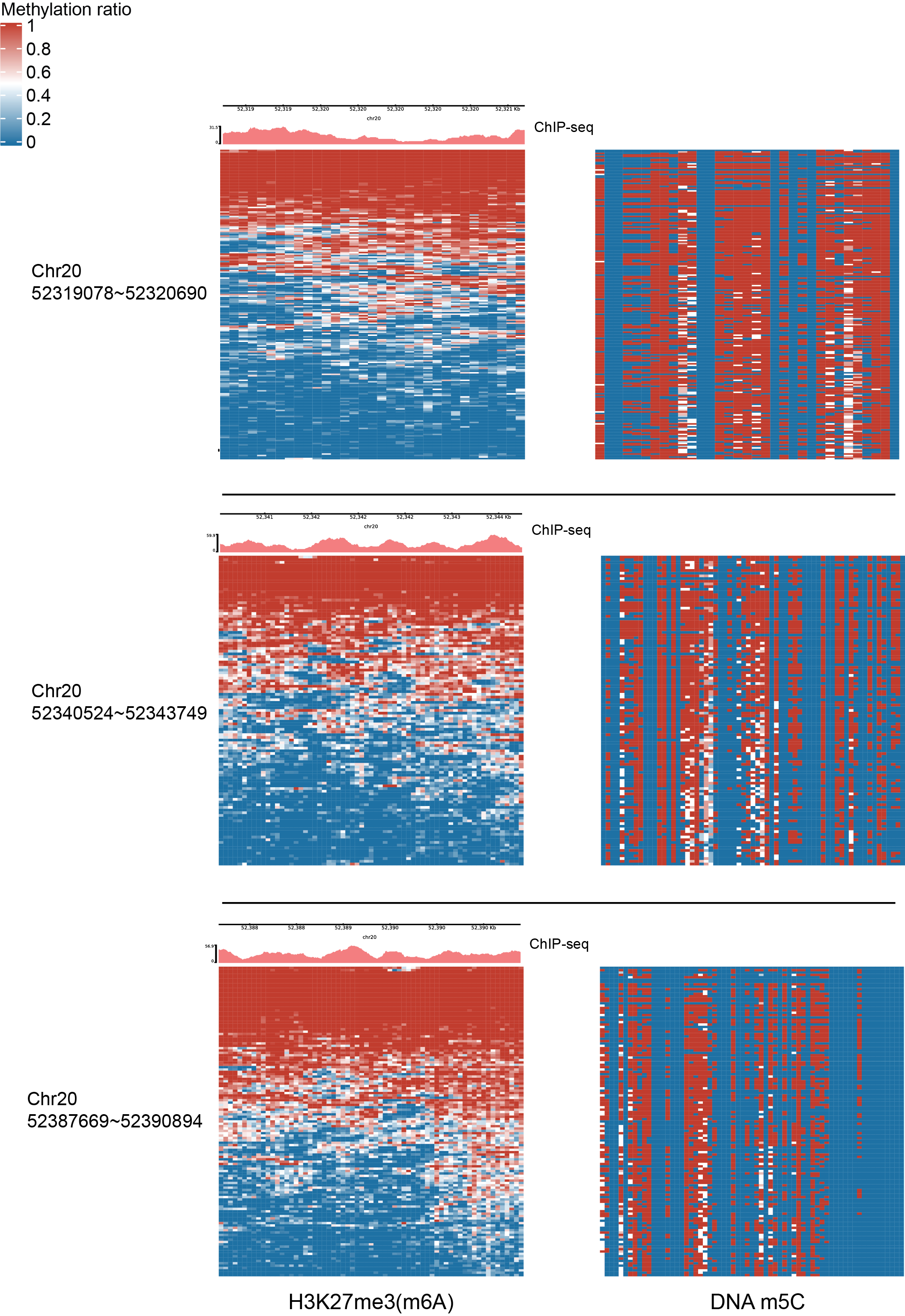


**Fig S8. The BIND&MODIFY showed the heterogeneity of H3K27me3 regulation.** The peak signal of H3K27me3 in ChIP-seq was shown in upper slides. The single molecular resolution visualized each molecular methylation statues of H3K27me3 on three genomic regions (Chr20:52319078~52320690, Chr20:52340524~52343749, Chr20:52387669~52390894). The rows in heatmap represented the different DNA molecules covered this region. The color indicated the methylation ratio, which represented the H3K27me3 signal. The right heatmap showed the CpG methylation distribution on the corresponding DNA molecules in the left H3K27me3 heatmap (one-to-one matching). The H3K27me3 signal decreased with the decreasing CpG methylation (the comparison between red and white signals in the CpG heatmap).


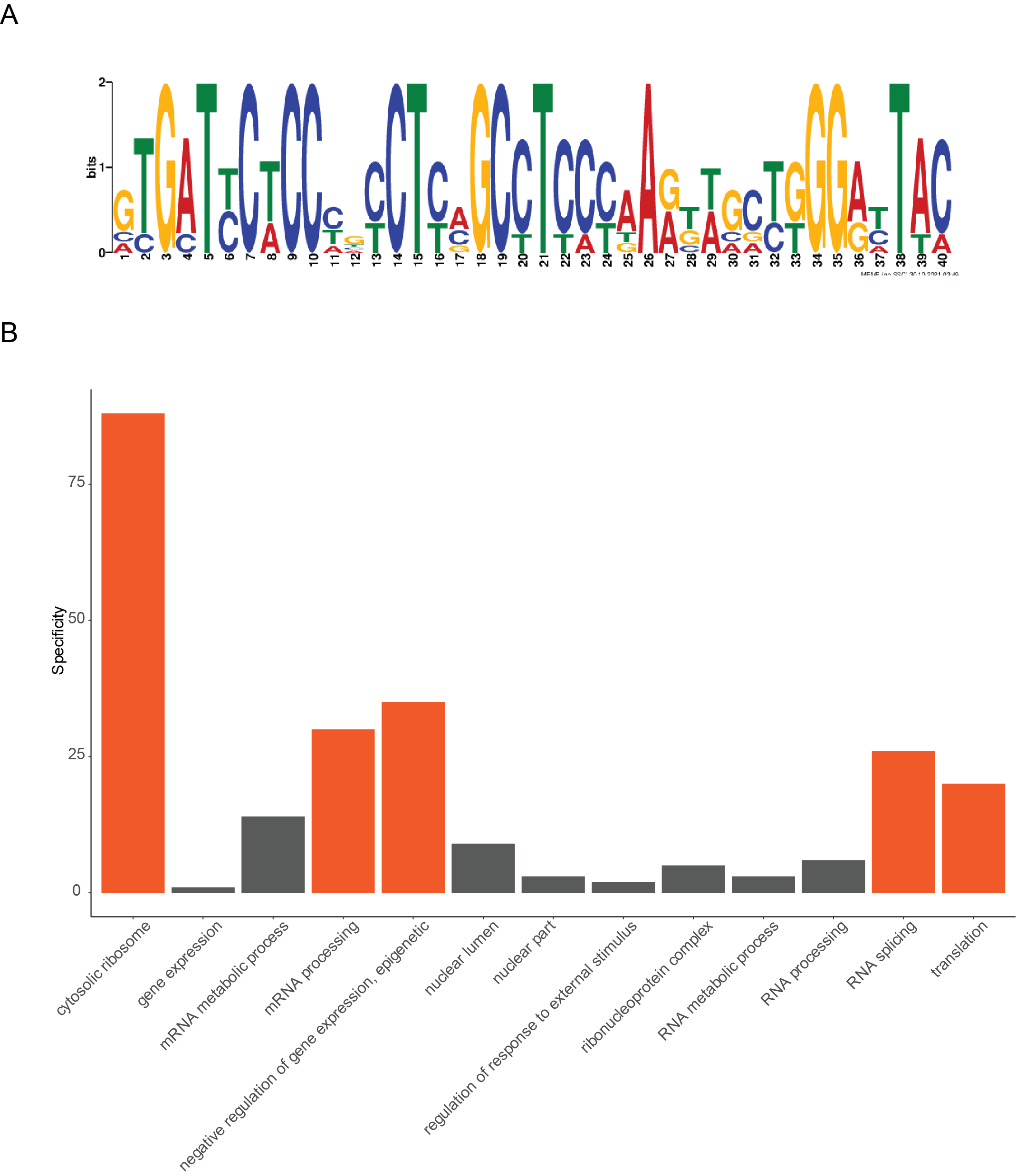


**Fig S9**. In the Figure 5-1 C, there were some strongly correlated regions on the 2kb upstream of the promoters. By the further motif analysis of these distant regions (A), we found that these regions may bind the RNA splicing and processing factors (B).


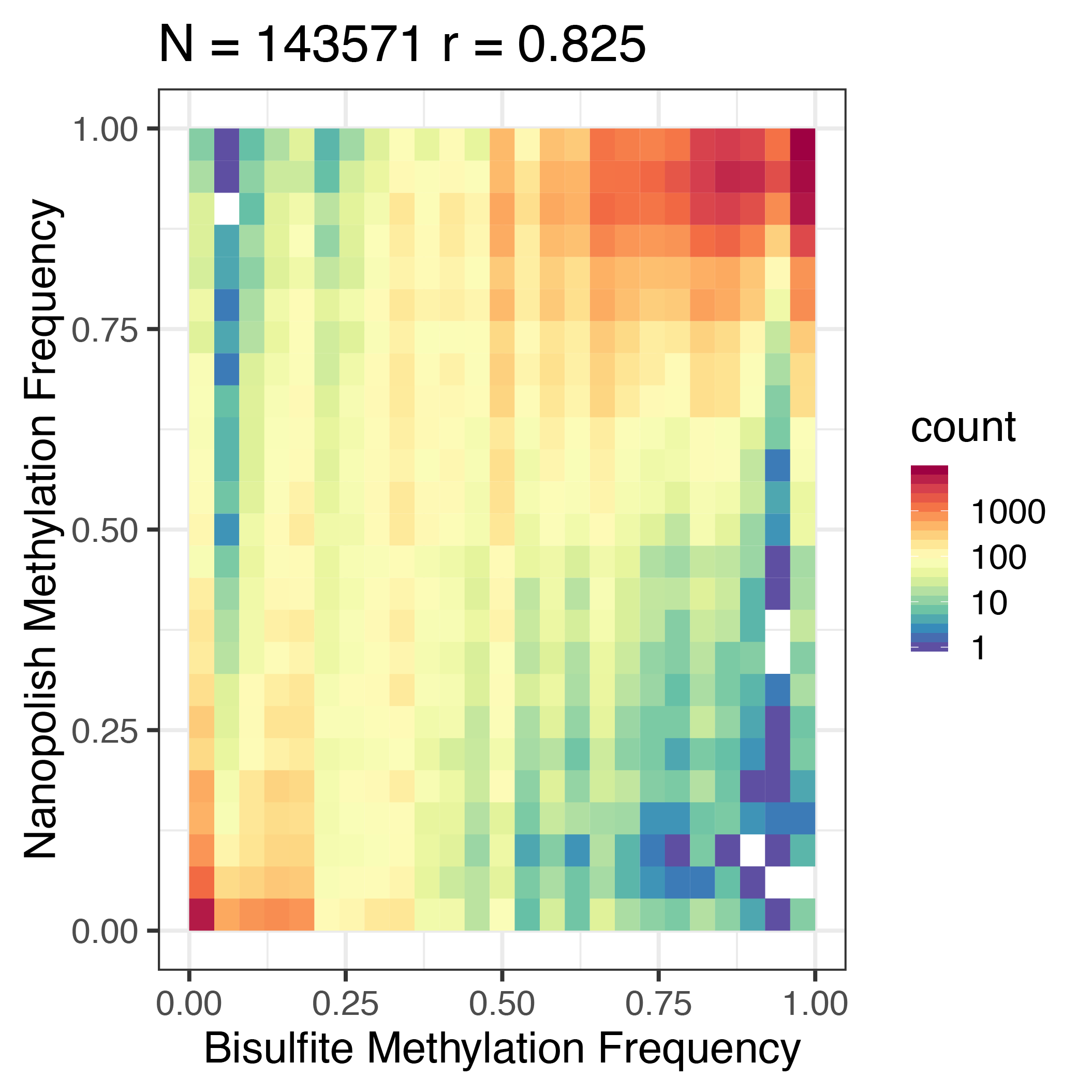


**Fig S10. The 5mC base calling correlation of MCF-7 H3K27me3 BIND&MODIFY samples with MCF-7 bisulfite sequencing.**


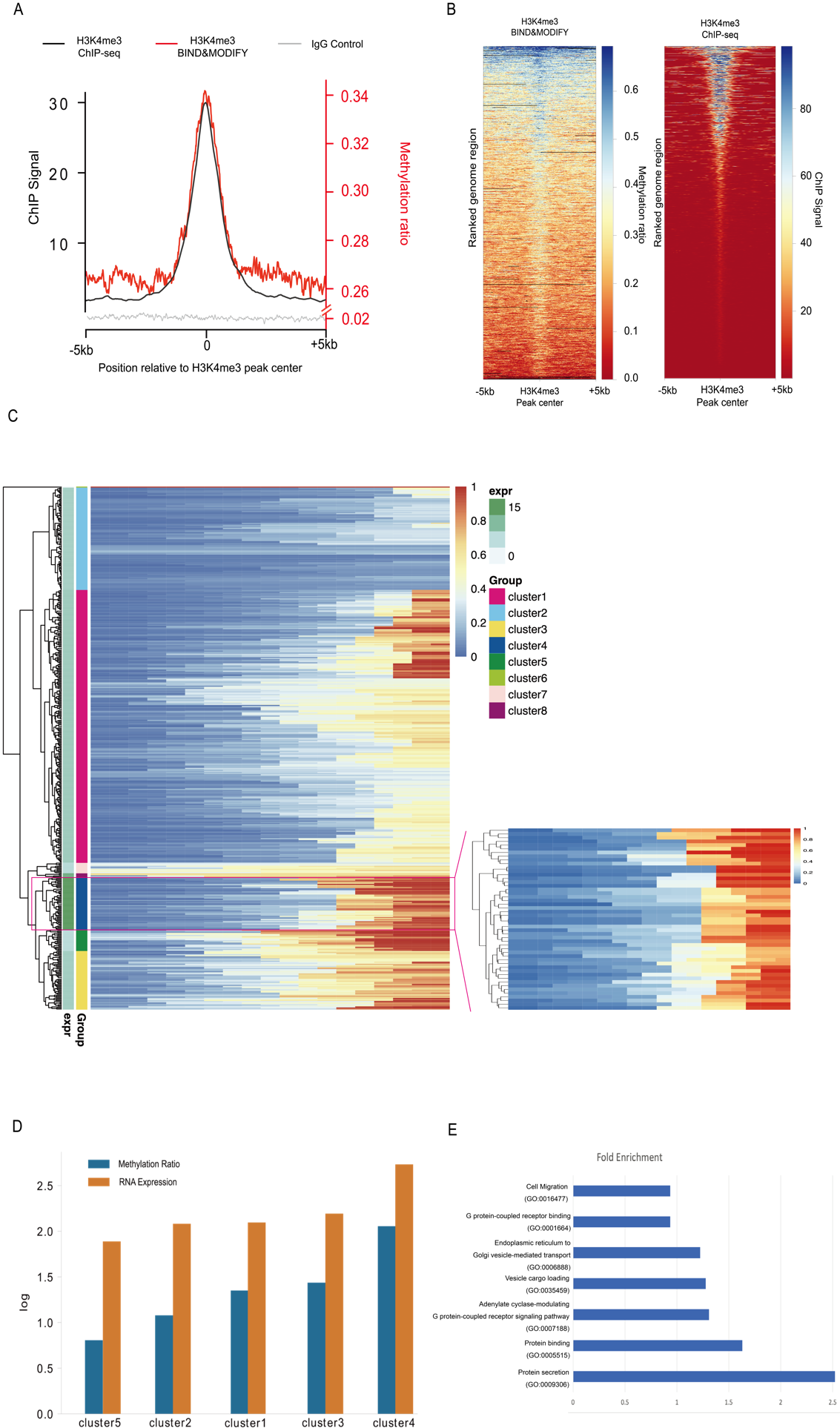


**Fig S11. The consistency of H3K4me3 pattern between ChIP-seq and BIND&MODIFY *in* *situ*.**

We carried out BIND&MODIFY experiments with HeLa cell line on histone modification marker, H3K4me3, which associates with active gene expression. (A) Comparing the peak distribution of H3K4me3 targeted BIND&MODIFY and ChIP-seq. The H3K4me3 peak regions were called by MACS in ChIP-seq. Then the middles of all peak regions were centered at 0. The upstream/downstream 5kb were plotted around the peak center 0 with sliding 100bp bins. BIND&MODIFY y-axis indicated the mean methylation ratio in 100bp bins, and ChIP-seq signal indicated the mean read counts. The signal of IgG control was low. (B) Mean m6A methylation ratios and ChIP-seq signal (sliding 100bp bins) were plotted around +/-5kb of H3K4me3 center for each gene of BIND&MODIFY and ChIP-seq. Color bar indicated mean methylation ratio. Each row indicated one genome region with H3K4me3 peak center of one certain gene. (C) H3K4me3 heterogeneity pattern of genetic promoters in Chr7 (method in Fig S18). Each pixel on each row corresponds to mean methylation ratio of each individual DNA molecule, and each row corresponds to each genetic promoter (2kb upstream of the transcription start site included most of the promoter regions). The left green bar (expr) showed the average expression value of the corresponding gene clusters. Cluster 4 with high RNA expression level were highlighted by purple box. (D) The bar plot showed positive correlation between RNA expression and H3K4me3 clustering. (E) The bar plot showed the GO enrichment analysis of the Cluster 4. Details of H3K4me3 heterogeneity calculation methods can be found in Fig S18.

**
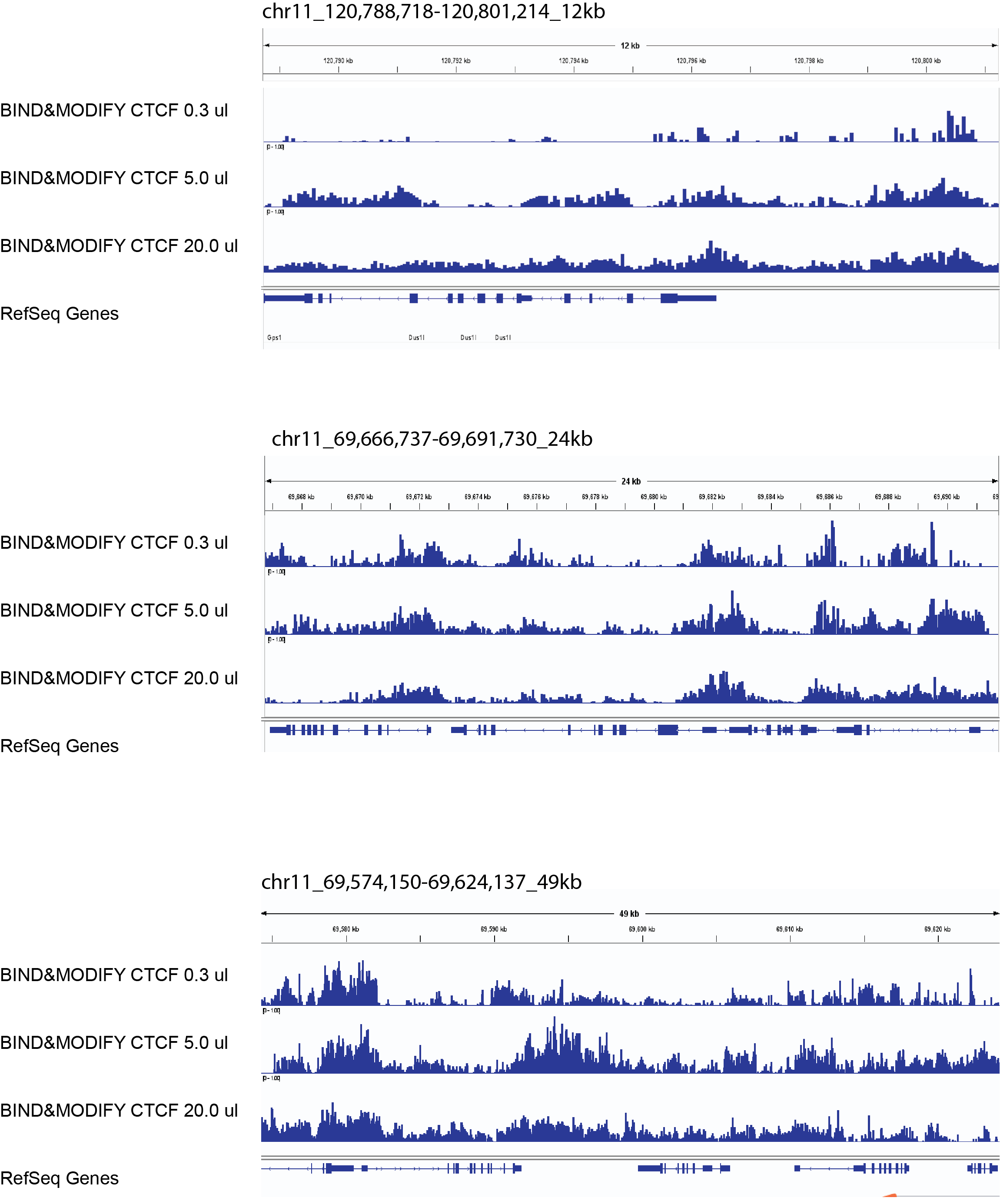
**

**Fig S12. The CTCF signal of different doses of pA-M.EcoGII by BIND&MODIFY in genome scale view.** 0.3ul, 5ul, 20ul of pA-M.EcoGII was applied on 4T1 cell line. CTCF signal was plotted on different genome scales. The y-axis indicate the m6A counts in bins.


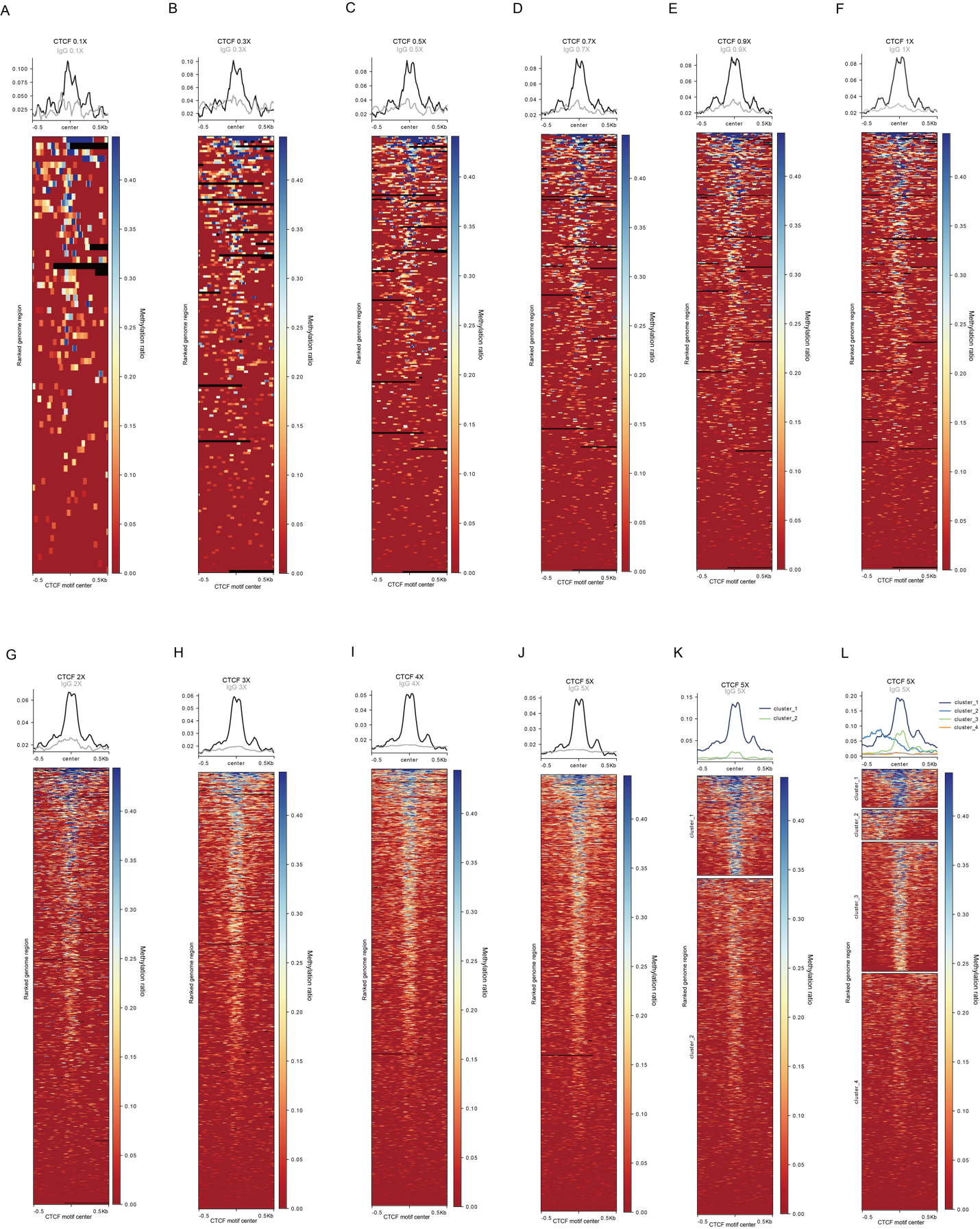


**Fig S13. Increase the sequencing depth of BIND&MODIFY targeting CTCF improves signal-to-noise ratio.**

(A-J) We increased the sequencing depth of BIND&MODIFY targeting CTCF, from 0.1X to 5X, and the CTCF motif centered peak distribution showed better signal-to-noise ratio while sequencing depth increased, The signal-to-noise ratio was calculated from peak:flanking region. For 0.1X, peak:flanking=0.08:0.03~2.5 folds; For 5X, peak:flanking=0.05:0.01~5 folds. (K-L) We further classified the 5X CTCF targeted BIND&MODIFY signal into 2 clusters (K) and 4 clusters (L), based on the methylation pattern over a 50bp bin sliding window of ranked gene body regions flanking +/-500bp of CTCF motif center.


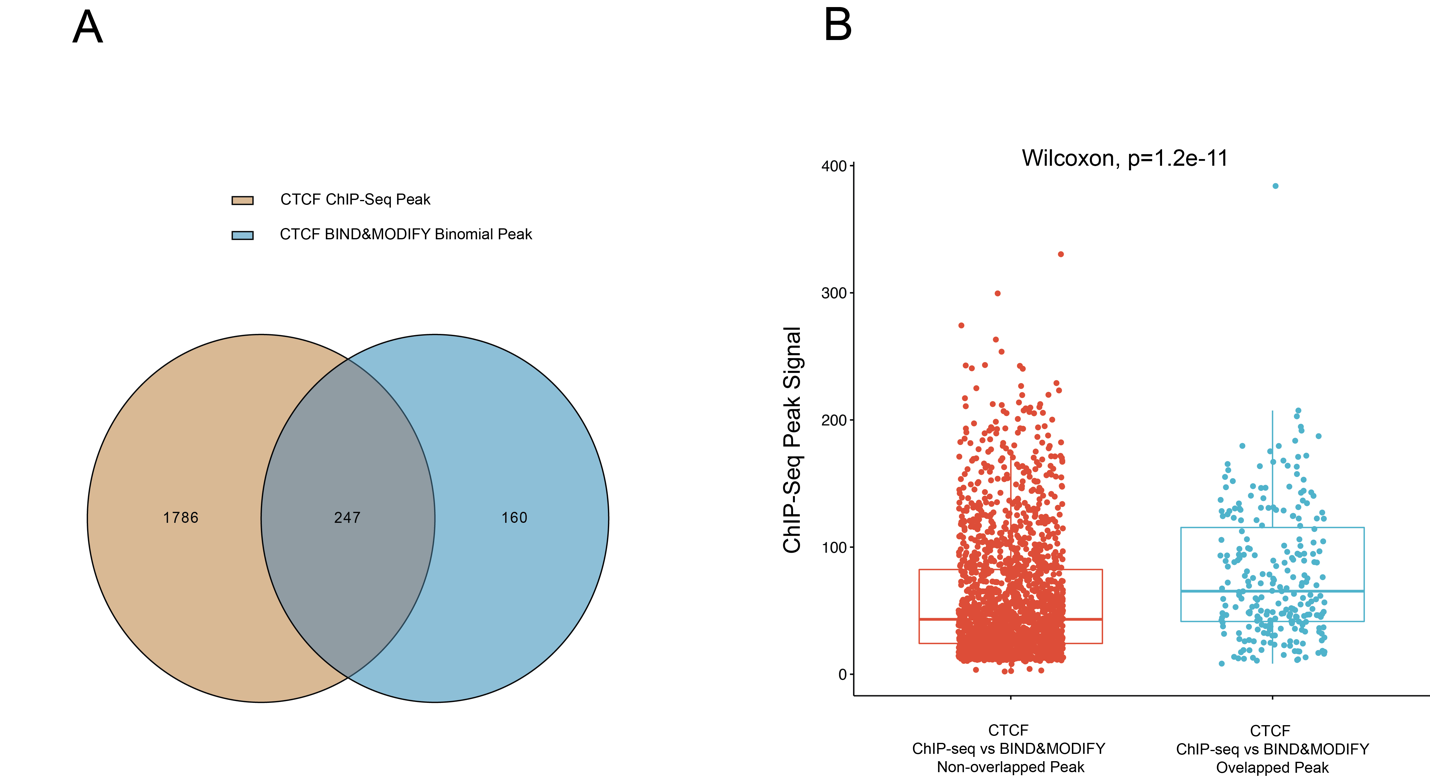


**Fig S14. The CTCF BIND&MODIFY overlaps with ChIP-seq signal.**

(A) Venn diagram of signals overlap (50bp windows) in CTCF ChIP-seq and BIND&MODIFY. The genome was segmentate to 50bp bins. The signal region in BIND&MODIFY was identified by m6A counts>1. The signal region in ChIP-seq was identified by reads number>0. Wynn map of CTCF BIND&MODIFY and ChIP-Seq showed overlap. The CTCF BIND&MOFIDY overlap was 60.7% (247/407), and CTCF ChIP-Seq overlap was 12.1% (247/2033)

(B) Wilcoxon signed ranks test of overlapped vs non-overlapped peak signal in CTCF ChIP-Seq. The overlapped peak had significant higher ChIP-Seq signal than non-overlapped peak (p=1.2e-11). (**Methods**, Peak overlap binomial test)


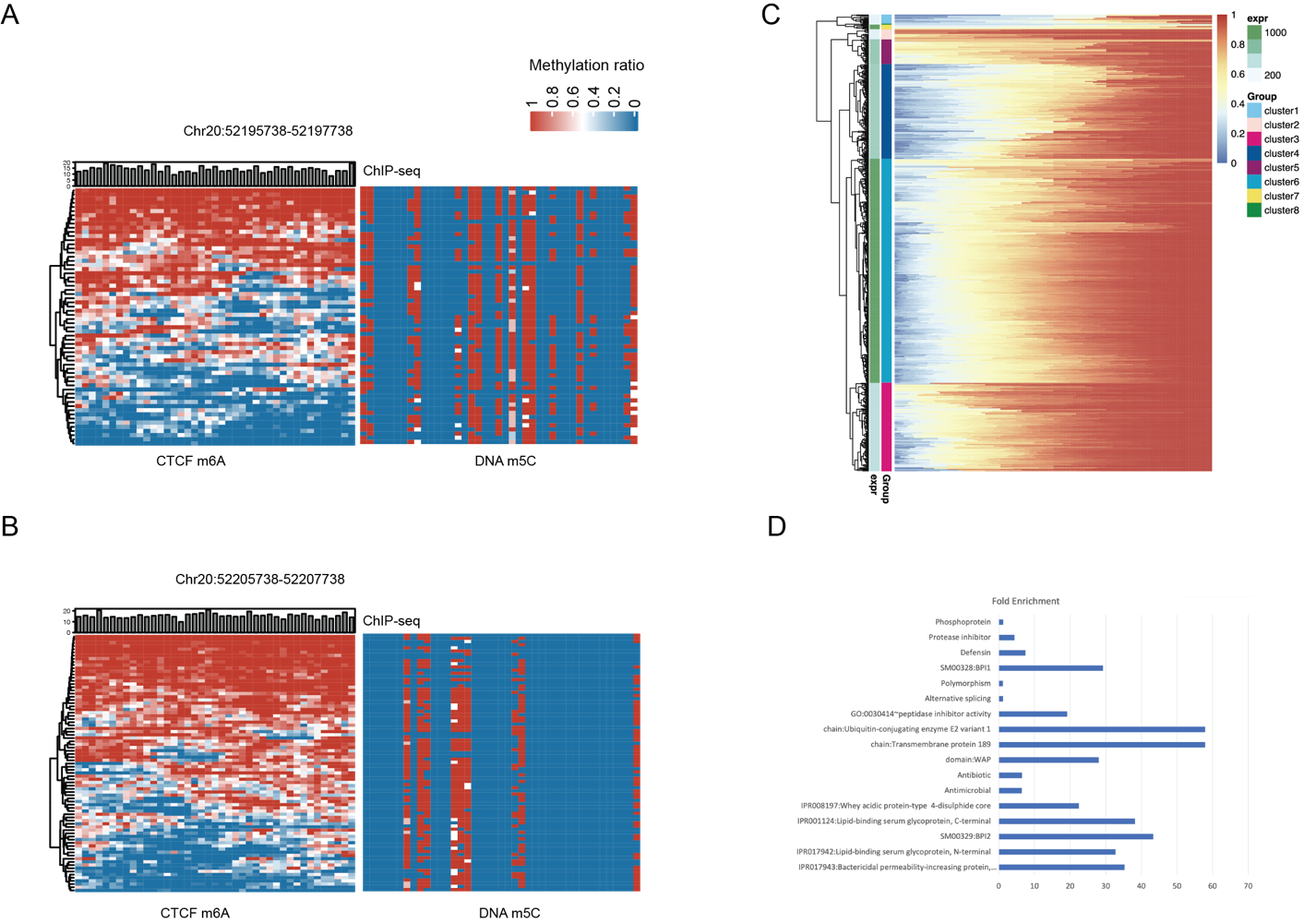


**Fig S15. The BIND&MODIFY showed the heterogeneity of CTCF regulation.** (A-B) The peak signal of CTCF in ChIP-seq was shown in upper panel. The single molecular resolution of the corresponding genomic region visualize each molecular methylation statues of CTCF. The rows in heatmap represented the different DNA molecules. The color indicated the methylation bin density, which represented the CTCF signal. The DNA molecules could be classified into three states: heavy state, medium state, and light state based on their methylation bin density. The right heatmap showed the CpG methylation distribution on the corresponding DNA molecules in the left CTCF heatmap (one-to-one matching). (C) CTCF heterogeneity pattern of genetic promoters in Chr20. Each pixel on each row corresponds to mean methylation ratio of each individual DNA molecule, and each row corresponds to each genetic promoter (2kb upstream of the transcription start site included most of the promoter regions). The methylation bin density was ranked from low to high (left to right). The left green bar (expr) showed the average expression value of the corresponding gene clusters. (D) The bar plot showed the GO enrichment analysis of the Cluster 1. Details of CTCF heterogeneity calculation methods can be found in Fig S18.


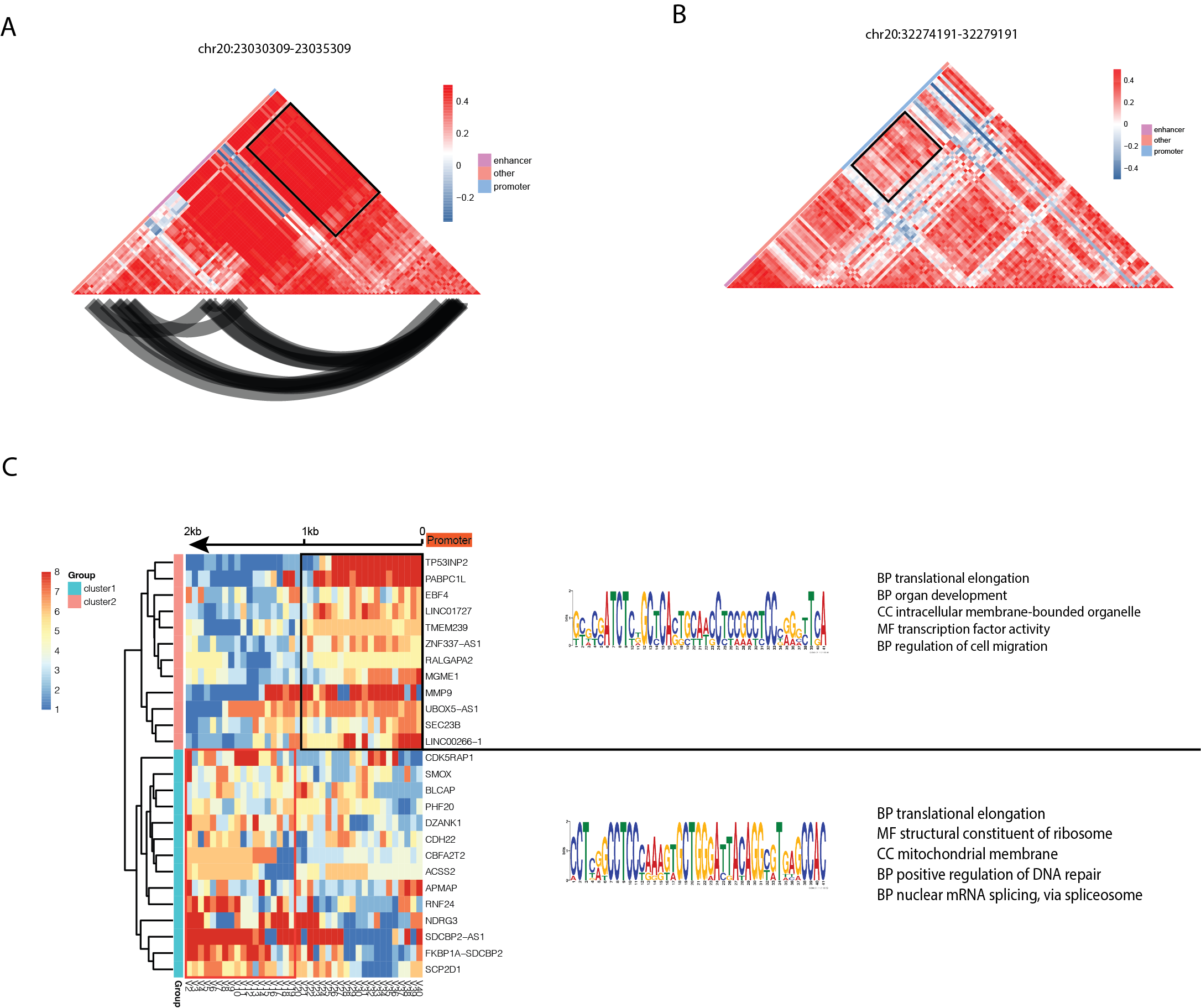


**Fig S16. The long-distance CTCF signal correlation between cis-regulators and promoters**. (A-B) We selected two genomic regions (chr20:23,030,309-23,035,309 gene *RP4-753D10.5*, chr20:32,274,191-32,279,191 gene *KIF3B*), which included the documented promoters and enhancers. Each pixel in the heatmap indicated the 50bp length. The correlation index calculated based on method. The higher correlation index (red) between two regions suggested the synchronization changes. We could observe the high correlation (black square) between the enhancers and promoters and other interaction regions. The lower black curves indicated the interaction signals in Hi-C data. The physically interacted regions (Hi-C) also had the synchronized regulation and the high CTCF correlation. (C) We further analyzed the promoter upstream 2kb regions to see whether there were some strongly correlated genomic areas, the potential cis-regulators for the promoters. The calculation details could be found on the method. Each pixel in the heatmap was 50bp length. Some prompters strongly correlated with their proximal regions (black box), suggesting that these proximal regions synchronized with the corresponding promoters in the CTCF regulation. By further analyzing the binding motif of these proximal regions, we found these regions have the binding motifs for the transcription regulators, modulating the transcription activity.


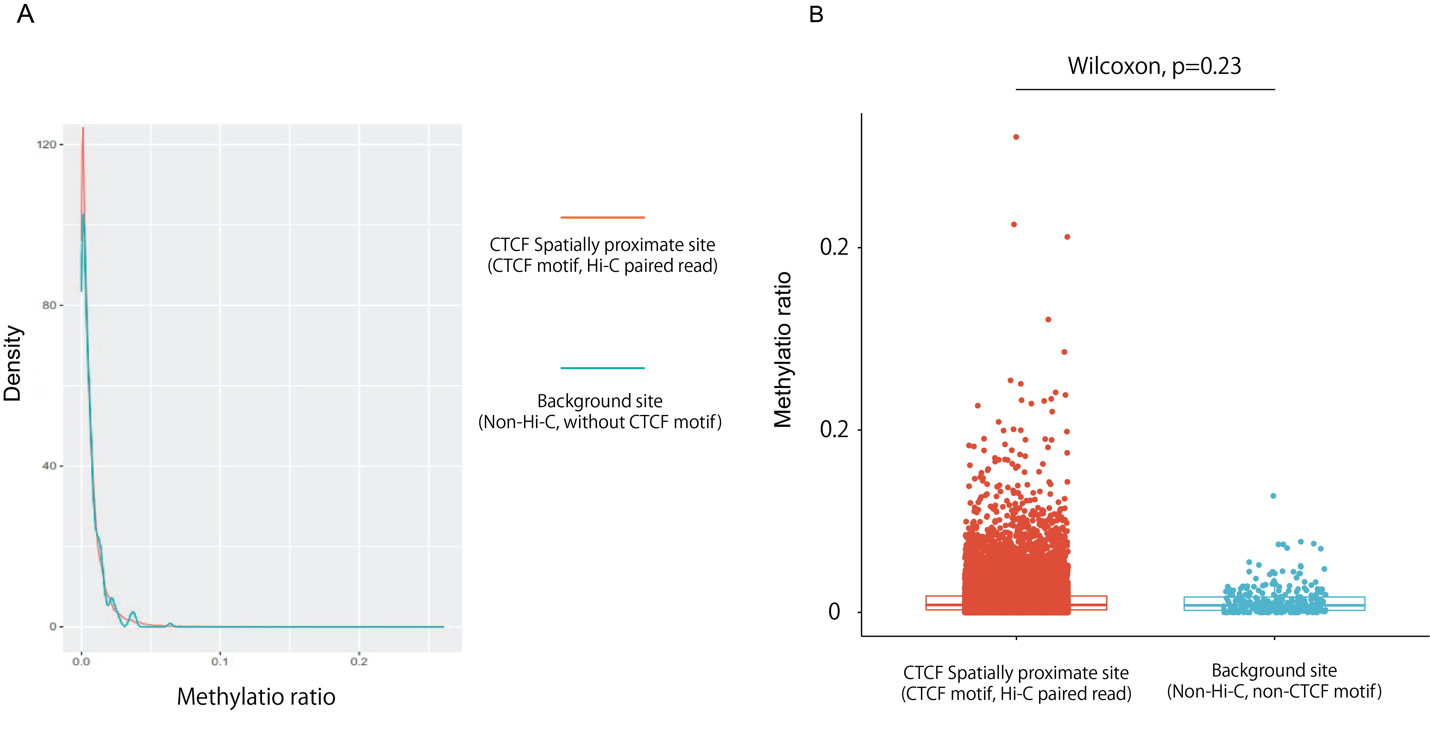


**Fig S17. The BIND&MODIFY could not efficiently label Hi-C spatially proximate region in *cis.***

We downloaded high confident Hi-C data (4DNFIKKZD99T). Based on the Hi-C data, we isolated the CTCF spatially proximate sites without CTCF motifs. These sites were spatially close to CTCF sites, detected by the Hi-C paired reads (one reads contain CTCF motifs, and the paired reads contain no CFCF motifs). We also randomly selected other background sites that neither contain CTCF motifs nor close to them (Group2, Background sites). We mapped the genome sites of the two groups to the CTCF targeted BIND&MODIFY data, measured the mean methylation ratio +/- 2.5kb of these two groups. (**Methods**, Comparing of CTCF targeted BIND&MODIFY Hi-C proximity region with background) (A) Methylation ratio distribution of these two groups. (B) Wilcoxon signed ranks test of these two groups. The CTCF spatially proximate sites does not showed statistically significant higher signal than background sites (p=0.23).


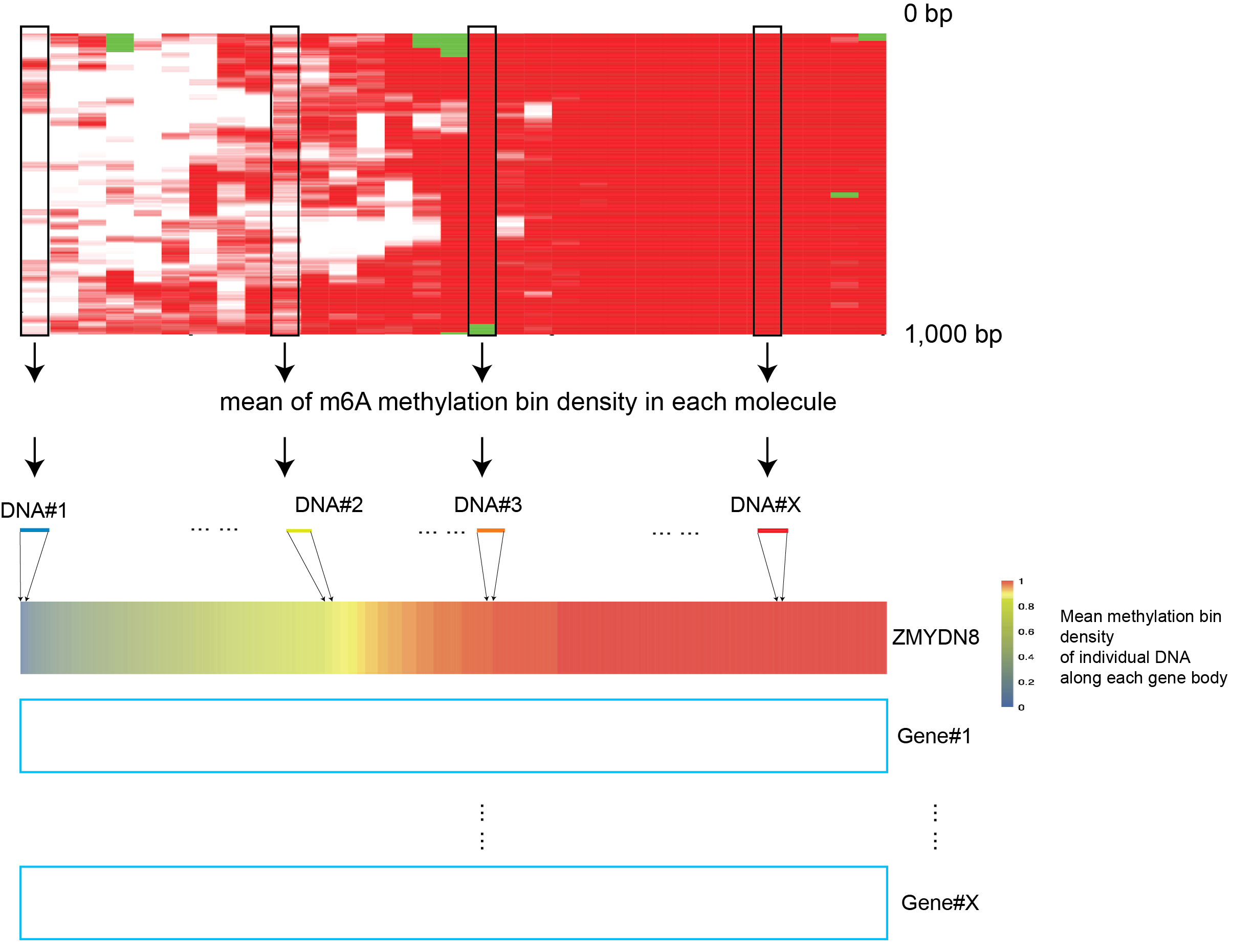


**Fig S18. Illustration of single molecule DNA heterogeneity clustering method. (Figure 4C).**

The upper heatmap indicated the molecular heterogeneity for the genome region (90 degree of Figure 5B). Each black square box indicated one molecule. The methylation density was calculated as the methylated bin (methylation ratio>0.5 with methylation probability cut-off>0.80 in bins) density (methylated bins/all bins) for all the single molecule DNAs that covered along each gene body, and subsequently all DNA molecules were ranked based on their methylation bin density for each gene. Hierarchical clustering of genes based on single molecular DNA heterogeneity was performed for the diversity of single molecule accessibility of each gene. (Methods, Single molecule DNA heterogeneity clustering)


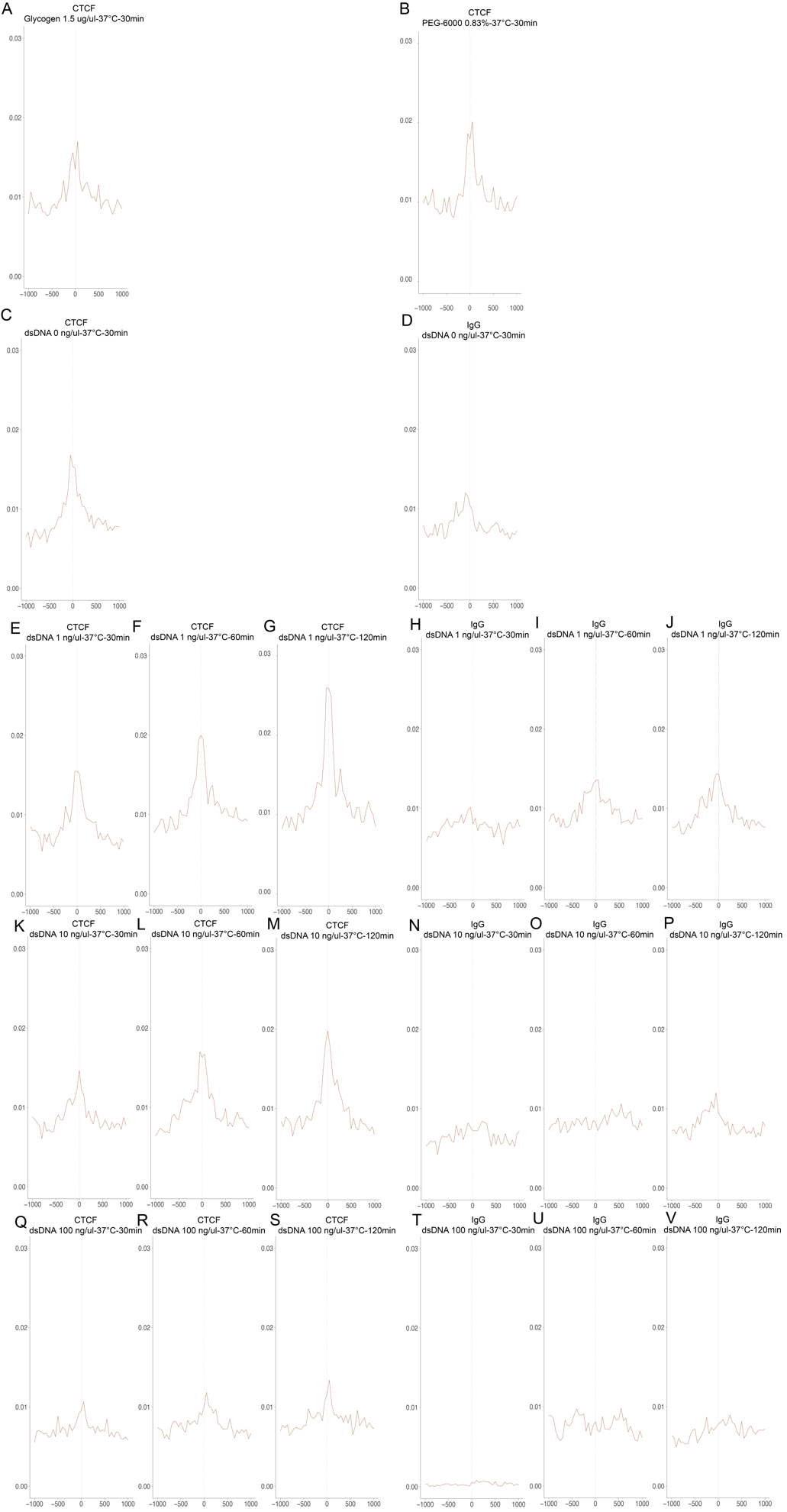


**Fig S19. The experiment protocol optimization for CTCF targeted BIND&MODIFY method.**

To systematic evaluate multiple experimental variables to find the best condition, we tested the following conditions, including:

1) add DNA crowding reagent, Glycogen, PEG-6000 to increase the specificity.

2) add dsDNA to bound the free floating pA-M.EcoGII to reduce the background noise.

3) increase reaction time from 30min to 120min to increase the signal-noise ratio.

For all the reactions, spermidine was introduced to 0.05mM in the methylation buffer.

(A) Addition of 1.5ug/ul Glycogen in the activation buffer, 37 degree 30min.

(B) Addition of 0.83% PEG-6000, 37 degree 30min.

(C) No additives, 37 degree 30min.

(D) Same condition as (C) for IgG.

(E-G) Addition of 1ng/ul dsDNA, 37 degree 30min, 60min, 120min.

(H-I) Same condition as (E-G) for IgG.

(K-M) Addition of 10ng/ul dsDNA, 37 degree 30min, 60min, 120min.

(N-P) Same condition as (K-M) for IgG.

(K-M) Addition of 10ng/ul dsDNA, 37 degree 30min, 60min, 120min.

(O-Q) Same condition as (K-M) for IgG.

(Q-S) Addition of 100ng/ul dsDNA, 37 degree 30min, 60min, 120min.

(T-V) Same condition as (Q-S) for IgG.


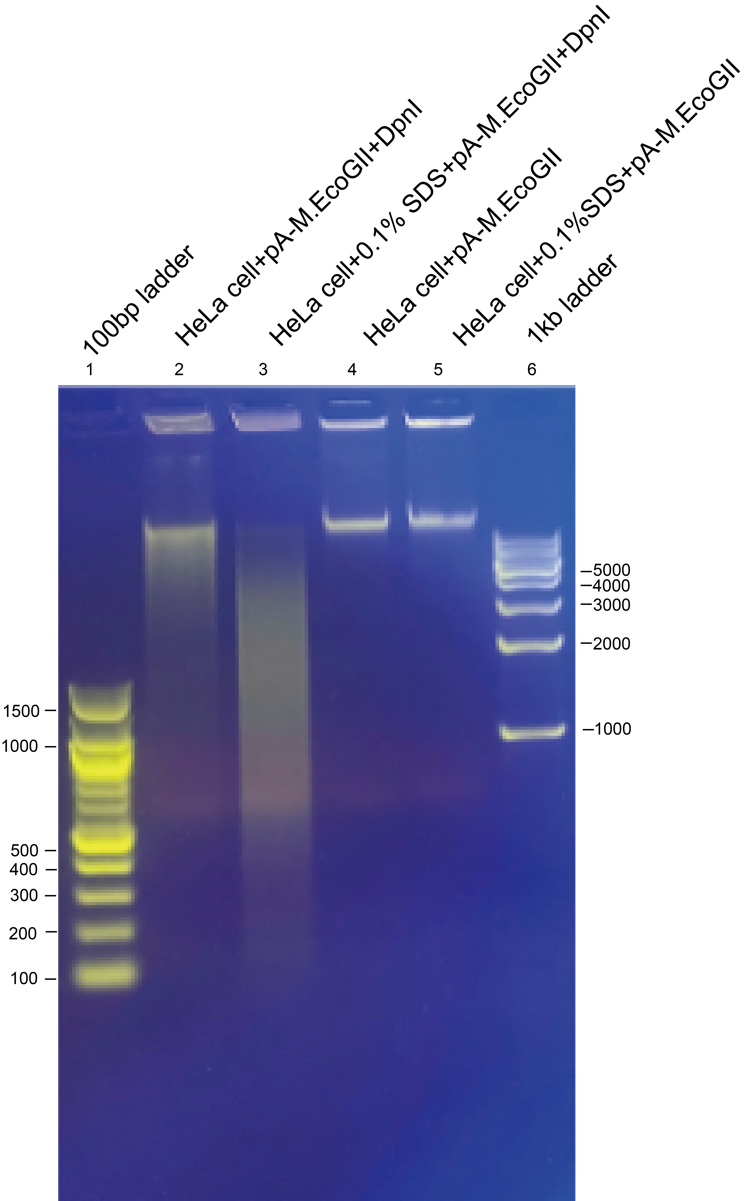


**Fig S20. Addition of SDS opens increases pA-M.EcoGII labeling efficiency on HeLa cell line.**

We tested the effect of addition of 0.1% SDS prior to antibody free labeling of pA-M.EcoGII labeling of HeLa cell line. Upon cell harvest, 1x10^6^ cells were fixed with 1% formaldehyde, after quenching with 0.125M glycine, the cells were treated with 0.1% SDS for 65 degree for 5min in Dig-wash buffer. SDS was quenched by 1% final concentration Triton X-100. After centrifuging at 2500rpm, the cells were treated with pA-M.EcoGII for 3h in methylation buffer (7.5ul 32mM SAM, 6ul 50X proteinase inhibitor, 1.5ul 20% BSA, 50ul pA-M.EcoGII, NF water up to 300ul), and replenish 7.5ul 32mM SAM and 10ul pA-M.EcoGII every 1 hour. Afterwards, the cells were lysed and total DNA were extracted by Seramega beads. 200ng of extracted 0.1% SDS(+/-) pA-M.EcoGII modified DNA was treated DpnI (+/-) digestion, and 1% agarose gel electrophoresis was performed.
