# Supplementary material for: BIND&MODIFY: a long-range method for single-molecule mapping of chromatin modifications in eukaryotes
Source: Genome Biol. 2023 Mar 29;24:61. doi: 10.1186/s13059-023-02896-y (PMC10052867; doi:10.1186/s13059-023-02896-y)
Supplement: Supplementary file 3 — Additional file 3. Review history. [file 13059_2023_2896_MOESM3_ESM.docx]

Review History

**First round of review**

**Reviewer 1**

**Were you able to assess all statistics in the manuscript, including the appropriateness of statistical tests used?**

No, I do not feel adequately qualified to assess the statistics.

**Comments to author:**

Wang et.al., utilized a non-sequence-specific adenosine methyltransferase EcoGII fused with targeted binding followed by Nanopore sequencing to detect chromatin protein binding together with DNA CpG methylation at single-molecule resolution. They first carried out systematic comparisons of BIND&MODIFY datasets with existing ChIP-seq datasets from the same samples to demonstrate the reliability of this method. They then showed the utility of using long-read sequencing in mapping epigenetic states in genomic regions with abundant repetitive sequences and exploring the possibility of identifying gene-CRE pairs by long-range epigenetic states correlative analysis.

I have a few major concerns on parts of this study:

1. From the current demonstration, the signal-to-noise ratio seems low to me.

For H3K27me3 BIND&MODIFY, the signal intensity (fig. 2C) of the peak center to flanking region is only 0.86-to-0.85. The methylation ratio difference between the flanking region and Ab-free control (0.85-to-0.77) is much higher than in the peak center-vs-flanking region. At the same time, I would expect them to have a comparable level if there are no confounding technical factors (i.e., concentration & activities of enzymes in different groups). Besides, the 4.2-to-3.9 ChIP-seq signal is also unreasonable low to me: H3K27me3 are typically broad peaks, and careful parameter tuning should be considered when using MACS software.

For CTCF, the ChIP-seq signal looks better (Fig. 2D, >15 fold enrichment). While for BIND&MODIFY, the s-n ratio is still not optimal, ~0.14/0.11 (~1.27 fold) for 1.25/0.3ul and ~0.17/0.13 (~1.30 fold) for higher concentration. And the methylation of flanking regions for 20ul&5ul is higher compared to lower concentration, again suggesting the concentration of enzyme may be a confounding technical factor contributing to background noise.

A systematic evaluation of enzyme concentrations will be beneficial for finding the optimal condition. In addition, an optimized bioinformatic algorithm (by identifying all mA bases across a region instead of calling individual mA) may also help improve the signal.

2. The comparison of BIND&MODIFY with H3K27me3 ChIP-seq is not stringent enough.

For Fig. 2B, the y-axis is not starting from 0. Thus, the data points for ChIP-seq with a lower signal are missing.

In Fig. S3C, what is the rationale for setting the cutoff to >1 m6A counts and >0 ChIP-seq reads? Under this cutoff, the combined regions cover>15% of the genome; for ENCODE H3K27me3 ChIP-seq, peak calling of the same cell line only covers ~1% of the genome.

3. The BIND&MODIFY is carried out in cross-linked cells. How do you distinguish if epigenomic states between correlated long-distance regulators are due to intrinsic concordancy or if it was methylated by EcoGII to spatially proximate regions in cis in cross-linked cells?

4. H3K27me3 is not a good target for correlating regulators. It repressed genes that are not expressed but repressed do not necessarily mean to activate. A mark associated with active gene expression, i.e., H3K27ac or H3K4me1, would be preferred to carry out such correlative analysis.


Minor points:

1. The size for each band in the ladder should be indicated in the figure panel (1D).

Besides, DpnI recognizes a GATC motif and is expected to distribute every ~256bp randomly; thus a smear would be expected after digestion. But several clear bands were observed in Fig. 1D; what are their sizes? Are they due to incomplete digestion or sequence biases?

2. Page11, line 7, is "experimental replicates" a technical replicate from the same cell culturing or a biological replicate with all materials independently prepared?

3. Page12, line 20, I didn't see "longer reads (PE150) generated 1% multiple alignments" from Fig. 3A.

4. The authors mentioned "co-accessibility assessment" in the methods section, but I didn't see any accessibility-related data in the main text.

**Reviewer 2**

**Were you able to assess all statistics in the manuscript, including the appropriateness of statistical tests used?**

Yes, and I have assessed the statistics in my report.

**Comments to author:**

In this manuscript, the authors Weng et al. demonstrated an exciting technique, BIND&MODIFY, to profile histone modifications and transcription factors together with CpG 5mC methylation, by tethering methyltransferase M.EcoGII to the protein binding sites followed by long length Nanopore sequencing. Compared with other techniques (e.g., Chip-seq and Cut&Run), this method has several unique advantages and especially fit for analysis of long range interactions of histone modifications. It is a novel technique that could be broadly helpful for labs working on epigenetic profiling. While I am generally satisfied with the analysis and data quality of the manuscript, there are several comments that should be fixed before publication:

1. In the background control (Supp Fig. 1A), there are some regions that show high m6A calling probability. What are these false positive sequences?

2. Similarly, in the treatment experiment (Supp Fig. 1B), there is a peak of the non-modified sites. Does this indicate any sequence bias of the methyltransferase M.EcoGII?

3. It would be helpful if the authors can evaluate if there is any bias of the methyltransferase M.EcoGII for tagging accessible vs. non-accessible regions.

4. For comparing Chip-Seq and the new methods, some details are missing: how many peaks are called from each method? How many peaks overlap with each methods? What is the overall multi-alignment rate between the two methods?

5. In Figure 2E, the CTCF signal from BIND&MODIFY is not so specific compared with the Chip-seq signal (right). Any reason about this? The authors should also check the signal correlation between Chip-seq and the new method (similar to Fig. 2B).

6. It is highly appreciated that the authors put the detailed protocol on protocol.io. For broad application of the method, it would be helpful if the authors can distribute the plasmid through public repositories (e.g., Addgene).

7. Page 10, Line 40, the longer reads (PE150) generated 1% multiple alignments - here it should be 25% based on Fig. 3A. Also the font size of several figures (e.g., Fig. 3AB) is too small and should be corrected.

**Authors Response**

**Point-by-point responses to the reviewers’ comments:**

*Reviewer #1: Wang et.al., utilized a non-sequence-specific adenosine methyltransferase EcoGII fused with targeted binding followed by Nanopore sequencing to detect chromatin protein binding together with DNA CpG methylation at single-molecule resolution. They first carried out systematic comparisons of BIND&MODIFY datasets with existing ChIP-seq datasets from the same samples to demonstrate the reliability of this method. They then showed the utility of using long-read sequencing in mapping epigenetic states in genomic regions with abundant repetitive sequences and exploring the possibility of identifying gene-CRE pairs by long-range epigenetic states correlative analysis.*

We thank the reviewer for the careful reading of our manuscript and their constructive feedback. We do appreciate their insightful suggestions. We carried out additional experiments and analyses to increase the coverage, improve the sensitivity, apply to active gene expression markers, and provide additional benchmarking of BIND&MODIFY method.

*I have a few major concerns on parts of this study:*

*1. From the current demonstration, the signal-to-noise ratio seems low to me.*

*For H3K27me3 BIND&MODIFY, the signal intensity (fig. 2C) of the peak center to flanking region is only 0.86-to-0.85. The methylation ratio difference between the flanking region and Ab-free control (0.85-to-0.77) is much higher than in the peak center-vs-flanking region. At the same time, I would expect them to have a comparable level if there are no confounding technical factors (i.e., concentration & activities of enzymes in different groups). Besides, the 4.2-to-3.9 ChIP-seq signal is also unreasonable low to me: H3K27me3 are typically broad peaks, and careful parameter tuning should be considered when using MACS software.*

A: We thank the reviewer for raising up this valuable suggestion about the broad peak nature of H3K27me3. We tried using broad peak configuration on MACS software following the reviewer’s suggestion, fine tuning the parameters, but it did not significantly improve the results. Through more investigation, we found that the H3K27me3 formed relatively continuous broad blocks, not sharp peaks, which was also kindly suggested by the reviewer. We also found that the H3K27me3 block sizes were not very uniform, ranging from 1kb to 10kb. Therefore, it is relatively difficult to reasonably normalize the H3K27me3 broad peaks into one single peak region (**Figure 2c**). Due to these reasons, the flanking region might carry the H3K27me3 signal from some adjacent ultra-wide blocks (**Reviewers Response Figure 1**). That caused the H3K27me3 flanking region to be much higher than the ab-free control. In this case, the suppressive marker H3K27me3 has intrinsic property of wide signal blocks, which hinders the calculation of signal-to-noise ratio, especially in the flanking region.

As the reviewer also suggested below in **Question 4**, we performed the additional experiment of BIND&MODIFY targeting H3K4me3, which are associated with actively expressed genes. We demonstrated that BIND&MODIFY targeting H3K4me3 had much better signa-to-noise ratio with a sharp peak distribution compared to the ChIP-seq (**Supplementary Figure 11**). Our data suggest that the histone modifications and DNA binding protein footprints with a sharp peak, such as H3K4me3 and CTCF, might be good candidates for signal-to-noise ratio calculation for BIND&MODIFY method.

**Reviewers Response Figure 1. Illustration of broad peak (H3K27me3) v.s. sharp peak.**

*For CTCF, the ChIP-seq signal looks better (Fig. 2D, >15 fold enrichment). While for BIND&MODIFY, the s-n ratio is still not optimal, ~0.14/0.11 (~1.27 fold) for 1.25/0.3ul and ~0.17/0.13 (~1.30 fold) for higher concentration. And the methylation of flanking regions for 20ul&5ul is higher compared to lower concentration, again suggesting the concentration of enzyme may be a confounding technical factor contributing to background noise.*

*A systematic evaluation of enzyme concentrations will be beneficial for finding the optimal condition. In addition, an optimized bioinformatic algorithm (by identifying all mA bases across a region instead of calling individual mA) may also help improve the signal.*

A: The reviewer raised good question about the CTCF signal-to-noise ratio and suggested methods how to improve the sensitivity of the BIND&MODIFY method. We believe it is important to tackle this question with two approaches.

Firstly, we investigated the effects of sequencing depth on the CTCF signal-to-noise ratio. We ran an additional sequence of CTCF-targeted BIND&MODIFY libraries and yielded a coverage from 0.1X to 5X to better distinguish the signal from noise. As shown in **Reviewers Response Figure 2**, we obtained a better signal-to-noise ratio of CTCF peak vs. flanking region when increased sequencing depth from 0.1X (CTCF peak: flanking=0.08:0.03,~2.5 folds) to 5X (CTCF peak: flanking=0.05:0.01, ~5 folds).

Secondly, the unsatisfied signal-to-noise ratio was caused by the non-enrichment method. Both Cut&Tag and ChIP-seq were PCR enrichment based, which only amplified the targeted fragments to enhance the signal-to-noise ratio. Compared with that, we used the non-enrichment direct sequencing of the methylated or unmethylated genome. That means we could get unbiased signals, which gave us the opportunity to see the truth of the target sites. To further demonstrate the non-enrichment effect, we identified several clusters which represent different CTCF binding frequencies and different signal-to-noise ratio (**Reviewers Response Figure 2**), representing the heterogeneity of CTCF at the single-molecule level. As we mentioned, if the sites have more targeted signals, the S-N ratio could be much higher. We also could optimize the S-N ratio of BIND&MODIFY by removing the non-labeled reads and converting them to the in-silico enrichment method. As the reviewer mentioned, we found that the enzyme amount and activity were confounding factors to background noise. However, the S-N ratio is not significantly changed with the different enzyme amounts. Therefore, we referred to the binomial algorithm to adjust the peak calling with the background methylation ratio.

Thirdly, we would need to further optimize the experimental protocol to improve the sensitivity of the BIND&MODIFY method. We thank the reviewer for this important comment about a systematic evaluation of enzyme concentrations for finding the optimal condition. We’ve carried out additional experiments to systematically evaluate multiple experimental variables to achieve better m6A methylation efficiency. The details of all tested experimental conditions are described in **Methods** section (Protocol V2, Manuscript, page 29, line 647-655) and **Reviewers Response Figure 3.** For the bioinformatics algorithms, we tried two popular algorithms Nanopolish and Megalodon. However, only Megalondon produced the best sensitivity and specificity.

In this revision, we also tested multiple experimental variables to optimize the best protocol of CTCF targeting BIND&MODIFY (**Reviewers Response Figure 3**). Firstly, we introduced the spermidine to 0.05mM in the activation buffer for all the conditions. Then we tested adding DNA crowing reagents Glycogen 1.5ug/ul or PEG-6000 0.83% in the activation buffer, and they showed not significantly better CTCF peak distribution. Further, we increased the incubation time from 30min to 60min and 120min and found 120min showed the best signal-noise ratio for CTCF peak distribution. Also, we added the primer annealed dsDNA (1ng/ul, 10ng/ul and 100ng/ul) to absorb the free-floating pA-M.EcoGII, and found dsDNA 1ng/ul was sufficient to give the best signal-noise ratio while maintaining minimal influence on the IgG background peak elevation. The sequencing depth for this experiment was about 1.0X for all the conditions tested. In this case, we found the best condition for the BIND&MODIFY reaction targeting CTCF, referred as Protocol V2. Some of them helped improve the methylation efficiency, but none of them fundamentally changed the results and conclusions from previous experiments by Protocol V1. We’ve published the most up-to-date version of the detailed experimental protocol, as Protocol V2, and can be accessed at Protocol.io with the following doi: ([dx.doi.org/10.17504/protocols.io.3byl4j7m8lo5/v1](http://dx.doi.org/10.17504/protocols.io.3byl4j7m8lo5/v1))

Further characterization of additional experimental conditions is under development, and we will keep the protocol.io online platform updated once we find better conditions to improve the methylation efficiency of the BIND&MODIFY method.

**Reviewers Response Figure 2. Increasing of BIND&MODIFY sequencing depth improves signal-to-noise ratio (A-G, Selected from Supplementary Figure 13).**

(A-E) We increased the sequencing depth of BIND&MODIFY targeting CTCF, from 0.1X to 5X, and the CTCF motif centered peak distribution showed better signal-to-noise ratio. (F-G) We further classified the 5X CTCF targeted BIND&MODIFY signal into 2 clusters (F) and 4 clusters (G), based on the methylation pattern over a 50bp bin sliding window of ranked gene body regions flanking +/-500bp of CTCF motif center.

**Reviewers Response Figure 3. The experiment protocol optimization for CTCF targeted BIND&MODIFY method.**

To systematic evaluate multiple experimental variables to find the best condition, we tested the following conditions, including:

1) add DNA crowding reagent, Glycogen, PEG-6000 to increase the specificity.

2) add dsDNA to bound the free floating pA-M.EcoGII to reduce the background noise.

3) increase reaction time from 30min to 120min to increase the signal-noise ratio.

For all the reactions, spermidine was introduced to 0.05mM in the methylation buffer.

(A) Addition of 1.5ug/ul Glycogen in the activation buffer, 37 degree 30min.

(B) Addition of 0.83% PEG-6000, 37 degree 30min.

(C) No additives, 37 degree 30min.

(D) Same condition as (C) for IgG.

(E-G) Addition of 1ng/ul dsDNA, 37 degree 30min, 60min, 120min.

(H-I) Same condition as (E-G) for IgG.

(K-M) Addition of 10ng/ul dsDNA, 37 degree 30min, 60min, 120min.

(N-P) Same condition as (K-M) for IgG.

(K-M) Addition of 10ng/ul dsDNA, 37 degree 30min, 60min, 120min.

(O-Q) Same condition as (K-M) for IgG.

(Q-S) Addition of 100ng/ul dsDNA, 37 degree 30min, 60min, 120min.

(T-V) Same condition as (Q-S) for IgG.

*2. The comparison of BIND&MODIFY with H3K27me3 ChIP-seq is not stringent enough.*

*For Fig. 2B, the y-axis is not starting from 0. Thus, the data points for ChIP-seq with a lower signal are missing.*

A: We thank the reviewer for bringing this up. In **Figure 2B**, we only compared the identified peak regions (which are not 0) from ChIP-seq to minimize the computation. We also carried out the global comparison of H3K27me3 targeted BIND&MODIFY vs ChIP-seq in **Supplementary Figure 3C**, also discussed below.

*In Fig. S3C, what is the rationale for setting the cutoff to >1 m6A counts and >0 ChIP-seq reads? Under this cutoff, the combined regions cover>15% of the genome; for ENCODE H3K27me3 ChIP-seq, peak calling of the same cell line only covers ~1% of the genome.*

A: We thank the reviewer for discussing the point of cut-off selection. As discussed in **Question1**, H3K27me3 might not be good candidate for calculating signal-noise ratio, as they have relatively broad peaks. In this case, most of the algorithms available are not suitable for the BIND&MODIFY H3K27me3 peak calling. We agree that the cut-off selection is important to identify the common features between BIND&MODIFY and ChIP-seq, though they also have substantial differences resulting from basic mechanisms, which leads to unique characteristics and applications.

Firstly, we used cut-off to >1 m6A counts and >0 ChIP-seq reads in **Supplementary Figure 3c** was originally meant to demonstrate the global picture of the overall signal overlap between BIND&MODIFY and ChIP-seq. Further, we tested different cut-off values for H3K27me3 ChIP-seq and BIND&MODIFY and plotted the signal overlap in **Supplementary Figure 3d**, and we found the cut-off values leads to different overlap between BIND&MODIFY and ChIP-seq. Generally, the overlap were 60%~80%.

To emphasize this point, we added the peak calling for CTCF targeted BIND&MODIFY in this revised manuscript with our newly developed binomial peak calling algorithm, which leads to a much higher signal-noise ratio for CTCF (Method section, page 40, line 865). We prepared Venn diagram based on CTCF targeted BIND&MODIFY and ChIP-seq peak calling data. As shown in **Supplementary Figure 14 a**, the CTCF BIND&MODIFY overlap is about 60.7% (247/407). Whereas the CTCF ChIP-seq overlap is about 12.1% (247/2033). This result is similar to other recently published soft labeling of chromatin accessibility methods using M.EcoGII methyltransferases like SOMASA(1), SMAC(2), and nanoNOME(2).

We further tested the overlapped vs non-overlapped CTCF ChIP-seq peak signal and Wilcoxon signed ranks test results in p=1.2e-11, which implies that there is a significant difference between overlapped vs non-overlapped ChIP-seq peak signal (**Supplementary Figure 14 b**). The CTCF ChIP-seq overlapped peaks showed significantly higher ChIP-seq signal than non-overlapped peaks, which means the high confident ChIP-seq peak mainly overlapped with the BIND&MODIFY peak signal.

*3. The BIND&MODIFY is carried out in cross-linked cells. How do you distinguish if epigenomic states between correlated long-distance regulators are due to intrinsic concordancy or if it was methylated by EcoGII to spatially proximate regions in cis in cross-linked cells?*

Newly prepared. Done.

A: We thank the reviewer for raising this important point about the artifact of pA-M.EcoGII labeling spatially proximate regions *in cis* in cross-linked cells. We agree with the reviewer that identification between the spatially proximate region in the cis and distant concordant region is important in the interpretation of long-distance co-labeling interactions. To answer this question, we downloaded the high confident Hi-C data (4DNFIKKZD99T), selected CTCF motif sites and their paired Hi-C contact sites (CTCF spatially proximate sites). If the BIND&MODIFY could efficiently label the proximate site, the CTCF paired contacted sites should demonstrate a higher signal than the background signals. However, the CTCF-paired Hi-C contact sites did not show a significantly higher signal than the background signal (**Supplementary Figure 17**). This data suggested that the BIND&MODIFY could not efficiently label the spatial proximate region, and the correlated long-distance regulators are due to intrinsic concordance without the artifact of pA-M.EcoGII’s spatially proximate labeling in *cis*.

*4. H3K27me3 is not a good target for correlating regulators. It repressed genes that are not expressed but repressed do not necessarily mean to activate. A mark associated with active gene expression, i.e., H3K27ac or H3K4me1, would be preferred to carry out such correlative analysis.* Newly prepared, partially done.

A: We thank the reviewer for the important comment about a marker associated with active gene expression. In the revision, we chose the histone modification marker, H3K4me3, which correlates with active gene expression(3), to further validate the general applications of BIND&MODIFY method.

To demonstrate the feasibility of H3K4me3 probing, we carried out additional experiments of BIND&MODIFY targeting H3K4me3 and plotted the benchmarking of ChIP-seq and BIND&MODIFY (**Reviewers Response Figure 4**) with our Protocol V2. The H3K4me3 ChIP-seq and BIND&MODIFY peaks showed very similar distribution patterns around the H3K4me3 peak center. Also of note, based on our newly developed experimental Protocol V2 discussed earlier in **Question 1**, the IgG background control signal was very low (~0.02) when compared with H3K4me3 (~0.34), which gives the about 17-fold signal-to-noise ratio on H3K4me4 based on current BIND&MODIFY Protocol V2 (**Reviewers Response Figure 4 a-b**).

To illustrate the power of probing single-molecule heterogeneity of H3K4me3 targeted BIND&MODIFY method, we plotted the single molecule clustering of all the promotor regions of genes on Chr7 based on the mean H3K4me3 methylation ratio (**Supplementary Figure 11 c**). Also, we benchmarked the RNA-seq data alongside the H3K4me3 single molecule clustering. We found that Cluster 4, with the highest RNA expression, also has the highest H3K4me3 methylation ratio on the promotors (**Supplementary Figure 11 c**). We plotted the RNA expression level against the H3K4me3 methylation ratio and found that they have a relatively good positive correlation (**Supplementary Figure 11 d**). Finally, we characterized the GO enrichment analysis on Cluster 4, and found they are associated with of protein secretion, G-protein coupled pathways, vesicle transport, and cell migration (**Supplementary Figure 11 e**).

Overall, to better benchmark the power of the BIND&MODIFY method in addressing single molecule heterogeneity, we applied BIND&MODIFY on H3K4me3, a histone marker associated with active gene expression(3) as the reviewer kindly suggested. And we found that H3K4me3 targeted BIND&MODIFY has similar peak distribution when compared to ChIP-seq and achieves a better signal-to-noise ratio with our updated experimental Protocol V2. And a good correlation of BIND&MODIFY single molecule heterogeneity is observed when compared with RNA-seq data.

**Reviewers Response Figure 4. The H3K4me3 targeted BIND&MODIFY H3K4me3 showed sharp peaks with Protocol V2**. **(Zoomed in from Supplementary Figure 11)** H3K4me3 targeted BIND&MODIFY data showed similar peak signal compared to ChIP-seq. (A) Comparing the peak distribution of H3K4me3 targeted BIND&MODIFY and ChIP-seq. The H3K4me3 peak regions were called by MACS in ChIP-seq. Then the middles of all peak regions were centered at 0. The upstream/downstream 5kb were plotted around the peak center 0 with sliding 100bp bins. BIND&MODIFY y-axis indicated the mean methylation ratio in 100bp bins, and ChIP-seq signal indicated the mean read counts. The signal of IgG control was low. (B) Mean m6A methylation ratios and ChIP-seq signal (sliding 100bp bins) were plotted around +/-5kb of H3K4me3 center for each gene of BIND&MODIFY and ChIP-seq. Color bar indicated mean methylation ratio. Each row indicated one genome region with H3K4me3 peak center of one certain gene.

Minor points:

*1. The size for each band in the ladder should be indicated in the figure panel (1D).*

*Besides, DpnI recognizes a GATC motif and is expected to distribute every ~256bp randomly; thus a smear would be expected after digestion. But several clear bands were observed in Fig. 1D; what are their sizes? Are they due to incomplete digestion or sequence biases?*

A: We thank the reviewer for raising up this point. We’ve added the 1kb ladder band marker to the figure panel (**Figure 1d**), as demonstrated below. To compare the m6A methylation-dependent DpnI restriction enzyme digestion performance of pA-M.EcoGII and commercial M.EcoGII, the experiment was designed using a 7k fragments of lambda DNA as substrate, which was PCR amplified from an original lambda DNA template. An equal amount of 7k lambda DNA was first methylated with M.EcoGII and pA-M.EcoGII accordingly. The methylated 7k lambda DNA was subsequently digested with DpnI, whose restriction digestion activity was dependent on the GATC motif with m6A methylation. Based on the GATC motif distribution on the 7k lambda DNA fragments, the GATC motif appeared at lambda DNA locations of 842, 1507, 2089, 2390, 2486, 2792, 2845, 4003, 4740, 4926, 5476, 6525, 6871, 6883 accordingly. Upon DpnI digestion, most of the bands are smeared, a few clear bands still exist. The sizes of the clear band are about 4200bp, 2100bp, 1800bp, 800bp. As the size of the clear bands is similar between commercial M.EcoGII and pA-M.EcoGII, which suggests they have similar m6A methyltransferase activity. The clear bands may be incomplete digestion from DpnI treatment as the input amount of 7kb lambda DNA was excess. Also of note, we carried out additional HeLa cell line pA-M.EoGII antibody free methylation experiment followed by DpnI digestion, the DNA gel was quite smear **(Reviewers Response Figure 5, lane2)**, which further supports our pA-M.EcoGII did have DNA m6A methylation activity. From the data above, we believe our expressed pA-M.EcoGII has similar m6A methylation activity compared to commercial M.EcoGII, as demonstrated by the DpnI cutting assay of lambda DNA.

*2. Page11, line 7, is "experimental replicates" a technical replicate from the same cell culturing or a biological replicate with all materials independently prepared?*

A: We thank the reviewer for mentioning this for clarification. The “experimental replicates” were from two different biological replicates with all materials independently prepared, which included preparing cells from two batches of cell culture with similar cell passage numbers, using the same batch of expressed pA-M.EcoGII enzymes, all other buffers were freshly prepared, and all other experimental procedures were followed by the BIND&MODIFY experiment protocol.

*3. Page12, line 20, I didn't see "longer reads (PE150) generated 1% multiple alignments" from Fig. 3A.*

A: We thank the reviewer for bringing up this point. We are sincerely sorry for the mistake. The correct sentence should be “the longer reads (PE150) generated 25% multiple alignments” (**Figure 3 a-b**). This figure has now been updated. We’ve corrected the description in the main text.

*4. The authors mentioned "co-accessibility assessment" in the methods section, but I didn't see any accessibility-related data in the main text.*

A: We thank the reviewer for raising up this issue. We apologize for the misuse of the term “co-accessibility assessment” in the method section. This term should be “co-labeling”, which describes the co-labeling of histone modifications (e.g. H3K27me3, H3K4me3, etc.) and DNA binding protein (CTCF) over long distances. This is one of the key advantages of the BIND&MODIFY method, which demonstrates the long-distance co-labeling of key histone modifications and DNA binding proteins, and their relationship with promotor-enhancer interactions. Data for this co-labeling analysis were discussed in detail in **Figure 4** and **Figure 5**.

*Reviewer #2: In this manuscript, the authors Weng et al. demonstrated an exciting technique, BIND&MODIFY, to profile histone modifications and transcription factors together with CpG 5mC methylation, by tethering methyltransferase M.EcoGII to the protein binding sites followed by long length Nanopore sequencing. Compared with other techniques (e.g., Chip-seq and Cut&Run), this method has several unique advantages and especially fit for analysis of long range interactions of histone modifications. It is a novel technique that could be broadly helpful for labs working on epigenetic profiling. While I am generally satisfied with the analysis and data quality of the manuscript, there are several comments that should be fixed before publication:*

We thank the reviewer for the careful reading of the manuscript and their enthusiasm for our technique. We do appreciate their valuable feedback. We carried out additional experiments and analyses to optimize the experiment protocol, increase the coverage, improve the sensitivity, and provide additional benchmarking of BIND&MODIFY method.

*1. In the background control (Supp Fig. 1A), there are some regions that show high m6A calling probability. What are these false positive sequences?*

A: We thank the reviewer for discussing about the negative control sample m6A base calling. There are various ways that may cause background m6A base calling signal from various m6A methylation probability. Firstly, we cannot completely rule out the existence of in vivo m6A modification from the samples. As suggested by a couple of literatures there are minimal genomic DNA m6A traces in mammalian cell lines, which may influence m6A base calling (4, 5). Secondly, in **Supplementary Figure 1 a-b**, the x-axis indicates the methylation probability (not methylation ratio). The methylation probability is generated from megalodon algorithm, which inform the probability of the true methylation in this site. Then we need to select the cut-off value to identify the methylated sites. Different methylation probability cut-off would generate a different false positive ratio (**Figure 1 g**). To balance the sensitivity and specificity, we chose the 0.53 cut-off with around 0.2 false positive ratio in our study. The higher cut-off value could significantly improve the false positive ratio.

*2. Similarly, in the treatment experiment (Supp Fig. 1B), there is a peak of the non-modified sites. Does this indicate any sequence bias of the methyltransferase M.EcoGII?*

A: We thank the reviewer for discussing about the full methylation control sample m6A base calling. As we discussed earlier in the previous **Question 1**, most of the sites in negative control with methylation probability do not indicate the methylation status without a cut-off value. The general cut-off value suggested by software and us is around 0.53. As we can observe in **Supplementary Figure 1 a-b**, a small fraction in negative control beyond the cutoff value 0.53. The selection of m6A methylation probability higher cut-off value would be the key to minimizing the false positive m6A base calling. In the fully methylated treatment experiment (**Supplementary Figure 1 b**), we found most of the methylation probability in positive control were beyond 0.53. We further increased the m6A methylation cut-off to 0.80 to calculate the receiver operation curve (ROC) in **Supplementary Figure 1 f-g**.

*3. It would be helpful if the authors can evaluate if there is any bias of the methyltransferase M.EcoGII for tagging accessible vs. non-accessible regions.*

A: We thank the reviewer for bringing up the important point of methyltransferase M.EcoGII labeling preferences between accessible and non-accessible regions. Originally, M.EcoGII was discovered to be a novel method of soft labeling chromatin accessibility at single molecule level(1, 2, 6). The ability to label accessible chromatin is the key feature of M.EcoGII. We believe that the M.EcoGII and other enzymes, such as Tn5, have more chances in attacking the accessible region, which is biochemically reasonable. Similar bias towards accessible chromatin regions was found in DiMeLo-seq(7) and Cut&Tag(8).

Indeed, we spent a lot of time optimizing our experiment protocol to resolve the question raised by the reviewers regarding accessible vs non-accessible regions. We carried out additional experiments to access the pA-M.EcoGII’s preferences for tagging DNA binding protein CTCF at open chromatin and histone modification H3K9me3/H3K27me3 at heterochromatin non-accessible regions. We found that the SDS could denature the heterochromatin and make them accessible for M.EcoGII (**Reviewers Response Figure 5**). Then we added the 0.01% SDS to the BIND&MODIFY experiment protocol prior to antibody binding. We found the SDS did not affect the CTCF labeling efficiency (data not shown). However, the SDS increased the methylation peak value of heterochromatin labeling by about 10% (data not shown). Both results supported that the SDS denaturing process could decondense heterochromatin.

Further characterization of experimental conditions is under development, and we will keep protocol.io online platform updated once we further better conditions to improve the methylation efficiency and signal-to-noise ratio of the BIND&MODIFY method.

**Reviewers Response Figure 5. Addition of SDS opens increases pA-M.EcoGII labeling efficiency on HeLa cell line.**

We tested the effect of addition of 0.1% SDS prior to antibody free labeling of pA-M.EcoGII labeling of HeLa cell line. Upon cell harvest, 1x10^6 cells were fixed with 1% formaldehyde, after quenching with 0.125M glycine, the cells were treated with 0.1% SDS for 65 degree for 5min in Dig-wash buffer. SDS was quenched by 1% final concentration Triton X-100. After centrifuging at 2500rpm, the cells were treated with pA-M.EcoGII for 3h in methylation buffer (7.5ul 32mM SAM, 6ul 50X proteinase inhibitor, 1.5ul 20% BSA, 50ul pA-M.EcoGII, NF water up to 300ul), and replenish 7.5ul 32mM SAM and 10ul pA-M.EcoGII every 1 hour. Afterwards, the cells were lysed and total DNA were extracted by Seramega beads. 200ng of extracted 0.1% SDS(+/-) pA-M.EcoGII modified DNA was treated DpnI (+/-) digestion, and 1% agarose gel electrophoresis was performed.

*4. For comparing Chip-Seq and the new methods, some details are missing: how many peaks are called from each method? How many peaks overlap with each methods? What is the overall multi-alignment rate between the two methods?*

A: We thank the reviewer for bringing up the details of comparing ChIP-seq and BIND&MODIFY. We developed a new peak calling algorithm for BIND&MODIFY to further benchmark the two methods. We stratified the BIND&MODIFY data over a 200bp bin sliding window, tested the binomial distribution of the resulting mean methylation ratio over IgG control, and decided whether a fragment can be called a BIND&MODIFY peak (details in Method section, page 40, line 865-875)

We prepared Venn diagram based on CTCF targeted ChIP-Seq and BIND&MODIFY and ChIP-seq peak calling data. As shown in **Supplementary Figure 14 a**, the CTCF BIND&MODIFY overlap is about 60.7% (247/407). Whereas the CTCF ChIP-Seq overlap is about 12.1% (247/2033). As ChIP-seq depends on PCR to amplify and enrich the targeted fragments, which leads to better sensitivity. The BIND&MODIFY unbiasedly sequenced all the labeled and unlabeled genomic DNA, which is the key feature of BIND&MODIFY directly labeling histone modifications and DNA binding proteins at a single molecule level without the bias introduced by PCR amplification and peak calling enrichment. A recent paper discussing soft labeling of DNA binding protein with methyltransferase also demonstrates a similar 30-40% on-target rate of CTCF probing ability (7). The deeper sequencing depth may produce more peaks to overlap with ChIP-seq. We are also working on a target enrichment method to further increase the coverage to achieve better sensitivity and specificity of DNA binding protein probing.

We further tested the overlapped vs the non-overlapped CTCF ChIP-Seq peak signal and Wilcoxon signed ranks test results in p=1.2e-11, which implies that there are significant differences between overlapped vs non-overlapped ChIP-Seq peak signal (**Supplementary Figure 14 b**). The CTCF ChIP-seq overlapped peaks showed significantly higher ChIP-seq signal than non-overlapped peaks, which means the high confident ChIP-Seq peak overlapped with the BIND&MODIFY peak signal. The multi-alignment rate for CTCF ChIP-seq was 25.91%, and for CTCF targeted BIND&MODIFY was 4.18% respectively.

*5. In Figure 2E, the CTCF signal from BIND&MODIFY is not so specific compared with the Chip-seq signal (right). Any reason about this? The authors should also check the signal correlation between Chip-seq and the new method (similar to Fig. 2B).*

A: We appreciate the reviewer for the suggestion to bring this issue on how to improve CTCF signal specificity as compared with the ChIP-seq signal. As we mentioned above, ChIP-seq is a PCR enrichment method, which is intrinsically different from the BIND&MODIFY. BIND&MODIFY unbiasedly sequenced all the labeled and unlabeled genomic DNAs. However, the sequencing depth should significantly enhance the specificity. We ran additional CTCF-targeted BIND&MODIFY experiments with additional ONT PromethION flowcells to increase our overall coverage to 5X for all CTCF peak calling. As shown in **Supplementary Figure 13**, we obtained a better signal-to-noise ratio of CTCF, and the heatmap showed a better peak signal around the CTCF motif center when sequencing depth increases to 5X.

To further explore this point that the unlabeled genomic DNA without the DNA binding CTCF proteins (cellular heterogeneity) could significantly affect the peak specificity, we classified the CTCF labeling into 5 clusters, from high to low labeling frequency, which represents the heterogeneity among the CTCF targeted BIND&MODIFY experiments. Obviously, the high labeling frequency sites have a significantly improved specific signal (**Supplementary Figure 13 k-l**). Another recently published m^6^A methyltransferase-based single molecule sequencing method also demonstrated similar CTCF binding heterogeneity, implies that about only 30% CTCF accessible sites has true CTCF protein binding events (9).

Due to the CTCF peaks being sharp (fewer reads in peak region), the correlation of ChIP-seq and CTCF targeted BIND&MODIFY needs to be established on the very deep sequencing data. For 5x data, the correlation is around 0.25. However, we expected the correlation should be higher with deeper sequencing depth as shown in DiMeLo-seq(7).

*6. It is highly appreciated that the authors put the detailed protocol on protocol.io. For broad application of the method, it would be helpful if the authors can distribute the plasmid through public repositories (e.g., Addgene).* done

A: We thank the reviewer for bringing up this point. We’ve published the most up-to-date version of the detailed BIND&MODIFY experimental Protocol V2. on protocol.io. ([dx.doi.org/10.17504/protocols.io.3byl4j7m8lo5/v1](http://dx.doi.org/10.17504/protocols.io.3byl4j7m8lo5/v1)).

We’ve also updated the Addgene page with our pA-M.EcoGII expression plasmid information (pTXB1-pA-M.EcoGII; [Addgene,192873](https://www.addgene.org/192873/)). We are in the process of plasmid deposition, and it’s estimated to be completed in less than a month or so. Once deposited, we will update the Genome Biology and the protocols.io page accordingly before finalizing the publication of this manuscript on Genome Biology.

*7. Page 10, Line 40, the longer reads (PE150) generated 1% multiple alignments - here it should be 25% based on Fig. 3A. Also the font size of several figures (e.g., Fig. 3AB) is too small and should be corrected.* done

A: We fully agree with the reviewer for kindly mentioning this point. We are sincerely sorry for the mistake. The correct sentence should be “the longer reads (PE150) generated 25% multiple alignments”. We thank the reviewer for this suggestion to improve the clarity of the figures. We’ve adjusted the font size accordingly. We believe the reviewer’s suggestion benefits from clarifying the original **Figure 3 a-b**. This figure has now been updated.

**References**

1.	Abdulhay NJ, McNally CP, Hsieh LJ, Kasinathan S, Keith A, Estes LS, et al. Massively multiplex single-molecule oligonucleosome footprinting. Elife. 2020;9.

2.	Shipony Z, Marinov GK, Swaffer MP, Sinnott-Armstrong NA, Skotheim JM, Kundaje A, et al. Long-range single-molecule mapping of chromatin accessibility in eukaryotes. Nat Methods. 2020;17(3):319-27.

3.	Barski A, Cuddapah S, Cui K, Roh TY, Schones DE, Wang Z, et al. High-resolution profiling of histone methylations in the human genome. Cell. 2007;129(4):823-37.

4.	Wu TP, Wang T, Seetin MG, Lai Y, Zhu S, Lin K, et al. DNA methylation on N(6)-adenine in mammalian embryonic stem cells. Nature. 2016;532(7599):329-33.

5.	O'Brown ZK, Boulias K, Wang J, Wang SY, O'Brown NM, Hao Z, et al. Sources of artifact in measurements of 6mA and 4mC abundance in eukaryotic genomic DNA. BMC Genomics. 2019;20(1):445.

6.	Chen W, Weng Z, Xie Z, Xie Y, Zhang C, Chen Z, et al. Sequencing of methylase-accessible regions in integral circular extrachromosomal DNA reveals differences in chromatin structure. Epigenetics Chromatin. 2021;14(1):40.

7.	Altemose N, Maslan A, Smith OK, Sundararajan K, Brown RR, Mishra R, et al. DiMeLo-seq: a long-read, single-molecule method for mapping protein-DNA interactions genome wide. Nat Methods. 2022;19(6):711-23.

8.	Wang M, Zhang Y. Tn5 transposase-based epigenomic profiling methods are prone to open chromatin bias. bioRxiv. 2021.

9.	Stergachis AB, Debo BM, Haugen E, Stirling CLS, Stamatoyannopoulos JA. Single-molecule regulatory architectures captured by chromatin fiber sequencing. Science. 2020;368(6498):1149-454.

**Second round of review**

**Reviewer 1**

The authors have adequately addressed most of my concerns. The remaining one will be minor and not affecting the main selling point of this manuscript.

For my previous major point 3: the current Supplementary Fig. 17b looks like there are differences; however, this may due to a large # of dots were crowded to 0 as S17a looks more comparable with each other. How many data points (and are the # of data points in CTCF site and background site the same) did the authors used for Wilcoxon test? I would suggested the authors to look into that again as the Wilcoxon test can be affected by sample size; and carefully discuss this part if it cannot be be robustly reproduced by other statistical methods.

**Reviewer 2**

The authors have satisfactorily resolved most of my comments. It is highly recommended to include the limitations of the methods (e.g., bias to accessible sites, relatively low correlation with the ChiP-Seq dataset) in the discussion section to increase the clarity of the manuscript. In addition, the new result about SDS treatment (i.e., Reviewers Response Figure 5. Addition of SDS opens increases pA-M.EcoGII labeling efficiency on Hela cell line) should be included as a key result in the manuscript. In addition, the authors should make sure the plasmid is accessible on Addgene before publication of the method.

**Authors Response**

**Point-by-point responses to the reviewers’ comments:**

Reviewer #1: The authors have adequately addressed most of my concerns. The remaining one will be minor and not affecting the main selling point of this manuscript.

For my previous major point 3: the current Supplementary Fig. 17b looks like there are differences; however, this may due to a large # of dots were crowded to 0 as S17a looks more comparable with each other. How many data points (and are the # of data points in CTCF site and background site the same) did the authors used for Wilcoxon test? I would suggested the authors to look into that again as the Wilcoxon test can be affected by sample size; and carefully discuss this part if it cannot be be robustly reproduced by other statistical methods.

A: We sincerely thank the reviewer for the comment about current Fig. S17b. Indeed, most data points are around zero. The data points for the two groups are CTCF Spatially proximate site (17960), and Background site (292) in the Wilcoxon test. As discussed in Fig. S17b, the background sites were selected that neither contain CTCF motifs nor were spatially close to them, which were not too many.

To further demonstrate the statistical robustness between the CTCF Spatially proximate site and the background site, we tested our data in two approaches.

Firstly, we carried out student t-test for the two groups. We added 0.00001 value to each of the data points and transformed the data points by log transformation to ensure normal distribution. As shown below (**Reviewer Response Figure** **1 B**), the student t-test resulted p value=0.11, no significance was found between the two groups.

Secondly, we down sampled the CTCF Spatially proximate site group, from 17960 to 292, which made the two groups had equal data points number. No significant difference was found between the two groups for both Wilcoxon test and student t test (**Reviewer Response Figure 1 C-D**). We further carried out 20 times down sampling, tested both Wilcoxon test and student t-test, and none returned significant difference between the two groups (**Reviewer Response Table 1**).

In this case, two statistical methods (Wilcoxon test and Student t test) resulted the same conclusion that there is no significant difference between CTCF Spatially proximate site and the Background site. However, notably, the CTCF spatially close sites still have some sites own the high signal above the noise. Therefore, we doubted some sites were proximately modified, but not significant enough to affect the results.

**Reviewer Response Figure 1 Demonstration of BIND&MODIFY could not efficiently label Hi-C spatially proximate region in *cis*, demonstrated by both Wilcoxon test and Student t test.**

We downloaded high confident Hi-C data (4DNFIKKZD99T). Based on the Hi-C data, we isolated the CTCF spatially close sites without CTCF motifs. These sites were spatially close to CTCF sites, detected by the Hi-C paired reads (one reads contain CTCF motifs, and the paired reads contained no CFCF motifs). We also randomly selected other background sites that neither contain CTCF motifs nor close to them (Group2, Background sites). We mapped the genome sites of the two groups to the CTCF targeted BIND&MODIFY data, measured the mean methylation ratio +/- 2.5kb of these two groups. (Methods, Comparing of CTCF targeted BIND&MODIFY Hi-C proximity region with background) (A)Wilcoxon test of the two groups (The original Supplementary Figure 17b) Data points in the two groups CTCF sites (17960), Background sites (292). (B) Student t test of the two groups. The data points were added 0.00001 and log transformed to ensure normal distribution for student t test. (C) Wilcoxon test of the down sampled equal CTCF sites (292) and background sites (292). (D) Student t test of the down sampled equal CTCF sites (292) and background sites (292).

**Reviewer Response Table 1**

|  | Wilcoxon Test | | Student t Test | |
| --- | --- | --- | --- | --- |
| Down Sampling | p value | p value adjust | p value | p value adjust |
| 1 | 0.701911111 | 1 | 0.41899795 | 1 |
| 2 | 0.408715159 | 1 | 0.37559143 | 1 |
| 3 | 0.783082631 | 1 | 0.52075454 | 1 |
| 4 | 0.237268798 | 1 | 0.20974676 | 1 |
| 5 | 0.086698370 | 1 | 0.11410295 | 1 |
| 6 | 0.293166976 | 1 | 0.08922257 | 1 |
| 7 | 0.678434453 | 1 | 0.25754464 | 1 |
| 8 | 0.278215653 | 1 | 0.07071744 | 1 |
| 9 | 0.270784512 | 1 | 0.09450238 | 1 |
| 10 | 0.768798938 | 1 | 0.52556696 | 1 |
| 11 | 0.191085202 | 1 | 0.12930284 | 1 |
| 12 | 0.607387947 | 1 | 0.55787360 | 1 |
| 13 | 0.655912391 | 1 | 0.32972548 | 1 |
| 14 | 0.601963776 | 1 | 0.21941645 | 1 |
| 15 | 0.231923788 | 1 | 0.23886062 | 1 |
| 16 | 0.062553376 | 1 | 0.04492695 | 1 |
| 17 | 0.147065989 | 1 | 0.10256386 | 1 |
| 18 | 0.286695150 | 1 | 0.07093219 | 1 |
| 19 | 0.208798725 | 1 | 0.36443275 | 1 |
| 20 | 0.310920369 | 1 | 0.63899912 | 1 |

Reviewer #2: The authors have satisfactorily resolved most of my comments. It is highly recommended to include the limitations of the methods (e.g., bias to accessible sites, relatively low correlation with the ChiP-Seq dataset) in the discussion section to increase the clarity of the manuscript. In addition, the new result about SDS treatment (i.e., Reviewers Response Figure 5. Addition of SDS opens increases pA-M.EcoGII labeling efficiency on Hela cell line) should be included as a key result in the manuscript. In addition, the authors should make sure the plasmid is accessible on Addgene before publication of the method.

A: We highly appreciate the reviewer’s suggestion to include the limitation of BIND&MODIFY methods in the discussion section. We’ve added the above-mentioned limitation points (bias towards accessible sites, relatively low correlation with the ChIP-seq dataset, low signal-to-noise ratio, etc.) in the discussion section. Further, as suggested by the reviewer, the CTCF targeting BIND&MODIFY experiment was added as **Fig S19**, and the SDS treatment experiment was also added as a key result in the manuscript as **Fig S20**. Finally, we’ve already submitted the pA-M.EcoGII plasmid to Addgene (<https://www.addgene.org/192873/>) and it passed Addgene QC. The plasmid will be released available before publication of the method.
